# The role of digital teaching methods in supporting practical skills training in the academic training of health professions – a scoping review

**DOI:** 10.1186/s12909-026-08785-9

**Published:** 2026-02-24

**Authors:** Tjorven Stamer, Nora Machinek, Jost Steinhäuser, Mathilde Goujard, Kristina Flägel

**Affiliations:** Institute of Family Medicine, University Hospital Schleswig-Holstein, Campus Luebeck, Ratzeburger Allee 160, Luebeck, 23562 Germany

**Keywords:** Digital teaching methods, Practical skills, Academic training, Undergraduate, Health education, Scoping review

## Abstract

**Background:**

Digital teaching methods are established for imparting theoretical knowledge in the training of health professions. While digital formats are increasingly used in education, evidence on their capacity to teach practical and hands-on skills remains limited. It is unclear to what extent digital teaching methods can support the training of practical skills. This scoping review aimed to identify the digital teaching methods used to teach practical skills in the academic training of health professions and to offer an initial evaluation of their effectiveness.

**Methods:**

A comprehensive literature search was performed in June 2023 using the PubMed, Scopus, Cochrane Library, Web of Science Core Collection and CINAHL databases. An update of this initial search was performed in April 2025. Original studies on the digital teaching of practical skills in undergraduate academic training for health professions, published in German or English, were included. The studies were analyzed with regard to teaching methods, areas of application, authors and year, intervention used, outcomes and results.

**Results:**

Out of 2,544 records, 292 studies were included. Interventions comprised simulators (*n* = 65), blended/hybrid teaching methods (*n* = 55), virtual reality (*n* = 48), e-learning platforms (*n* = 36), video-based teaching formats (*n* = 19), telecommunication tools (*n* = 18), mobile applications (*n* = 13), robotic simulators (*n* = 10), serious games or gamified learning (*n* = 10), specialized tools (*n* = 6), interactive learning systems (*n* = 6) and augmented reality (*n* = 6).

75% of the studies focused on medical education, followed by student training in nursing (18%) and physiotherapy (3%). The most common study designs were randomized controlled trials (42%), quasi-experimental designs (34%) and quantitative-descriptive designs (12%). Learning outcomes were predominantly assessed through knowledge tests, practical examinations (e.g., OSCEs) and participants' self-assessments. Across the included studies, most digital teaching methods demonstrated positive effects on the acquisition of practical skills, particularly simulation-based, VR-supported or otherwise interactive formats. The magnitude and consistency of these effects varied substantially across disciplines and study designs.

**Conclusions:**

Digital teaching methods offer promising approaches to support the training of practical skills, with interactive, simulation-based, and VR-supported formats showing particularly positive effects. Nevertheless, longitudinal studies examining the retention of practical skills over time remain scarce. Digital teaching methods should be specifically tailored to the practical skills being taught, making use of available resources.

**Supplementary Information:**

The online version contains supplementary material available at 10.1186/s12909-026-08785-9.

## Background

In any health care profession, practical skills are crucial for successful practice [1]. These relate to core competencies such as patient-centered care, working in interdisciplinary teams, using evidence-based practice, applying quality management and using new technologies and equipment [2]. Teaching and training methods used to train practical skills are diverse and range from the teaching of singular practical skills on site to high-fidelity simulations [3]. Since hands-on practice in health professions is often associated with at least some degree of risk, stress or uncertainty, e.g. in a surgical context, suitable methods to support the development of appropriate skills are of great importance [4]. To ensure efficiency and effectiveness, teaching methods are supposed to be directly related to the needs of the learners [5, 6]. In addition to traditional teaching methods such as lectures, seminars and labs, the blended learning format, i.e. the combination of face-to-face classroom instruction with online learning activities, has grown significantly in recent years [7]. The same goes for e-learning teaching formats, i.e. the use of digital technologies and the internet to deliver educational content, enabling learning anytime and anywhere [8]. Especially the latter concept allowed for continuity of teaching and learning during the COVID-19 pandemic as of 2019 [9]. COVID-19 led to an accelerated adoption of digital tools for knowledge transfer and skill practice [10]. Although many digital methods adopted during the COVID-19 pandemic were not retained thereafter, blended and e-learning formats continued to be used beyond the pandemic [11, 12].

### Digital teaching and learning

Digital teaching allows learning to be independent of time and place, which is beneficial for the individual needs of students [13]. It also gives learning a continuous character, which benefits the continuous growth of knowledge. In addition, digital teaching, such as online courses, makes it easier to scale courses to larger numbers of students [14]. New learning methods and technologies, such as gamification, virtual reality (VR), augmented reality (AR) and tools based on artificial intelligence (AI) and machine learning (ML) make it possible to visualize learning processes in an effective and impressive way, for example through simulations [13–15]. Digital learning management systems and tracking tools can be used to comprehensively record learners’ progress and to evaluate it in detail using technical analysis methods [13, 14]. Furthermore, digital teaching methods promote independent and collaborative learning and help to develop and expand digital skills [13, 14]. Previous studies show that digital teaching can promote the understanding and long-term retention of knowledge [15, 16].

Transitioning to and maintaining digital teaching presents challenges and concerns for every profession in the healthcare sector [17]. Due to the diversity of the type of practical work in the individual health professions, these challenges and concerns are strongly individual and are based on the procedures and instruments specified by the specialist field [18]. While simulators are used for surgical procedures in surgery, for instance, video-based teaching is often used in nursing [19, 20]. Interdisciplinary challenges include infrastructural and technical barriers, psychological and personnel-related hurdles and concerns about increased workloads and working hours [18]. Also, digital teaching is demonstrably less effective for teaching social skills [13, 14].

In summary, digital teaching in health professions education should be an integral part of the curriculum to teach digital skills such as the use of AI, telemedicine and electronic health records in a practical way [13, 14]. The use of innovative methods such as blended learning, inverted classroom, gamification and VR/AR is recommended to make learning processes interactive and viable [13–16]. Hence, continuous didactic and technical development of digital teaching is essential to ensure the quality of education in the long term [21].

In this review, we intentionally focused our search on academic training settings because digital teaching methods are most systematically developed, implemented, and evaluated within higher-education programs [13, 15]. Academic curricula offer standardized competency frameworks and structured assessment formats, which enable more consistent identification, comparison, and synthesis of practical-skills training across studies [13–15]. Furthermore, the majority of existing research on digital instruction in the health professions is conducted in university-based settings [21]. For these reasons, non-academic training pathways were not included in the scope of this review.

### Definitions of practical skills

In this article, we use “practical skills” to mean patient-oriented, manual, and technical procedures - that is, concrete hands-on tasks performed directly on or with patients [22]. In healthcare, they complement emergency and communication skills [22]. The 2002 amendment of the German Medical Licensing Regulations highlighted the importance of teaching practical competencies, including procedural skills, in medical curricula [23]. Subsequently, the DACH (German, Austrian, Swiss) Association for Medical Education (GMA) published a consensus statement defining 289 essential practical skills, 92 of which were integrated into a dedicated chapter of the National Competence-Based Learning Objectives Catalogue for Medicine [22, 23].

### Research questions

Various digital teaching and learning methods, such as simulations and virtual patients, have been studied in several health professions, including medicine, nursing and physiotherapy [24]. Despite this, there is no clear evidence on the explicit effect of the use of digital methods on the acquisition and maintenance of practical skills in academic health professions. In addition, there is a lack of a more holistic view of how both, educators and learners, can benefit from the use of digital methods in their respective health professions.

To address this need for a comprehensive, general overview of the heterogeneous evidence base, a scoping review was found to be best suited, as the evidence base is highly heterogeneous and spans many intervention types, professions and outcomes [25].

Therefore, in order to provide this perspective, this scoping review aimed to identify the areas of application, types of digital interventions and outcomes associated with the use of digital technology for teaching practical skills in the academic health professions. Based on this aim, the following research questions were formulated:

1. In what ways are digital teaching methods used to support the teaching of practical skills in the academic health professions?
2. What domains, types of digital interventions and reported outcomes characterize the studies examining the use of digital technology for teaching practical skills and what do these studies report regarding the effectiveness of the digital interventions used?

## Methods

### Databases used and search strategy

This scoping review used the PRIMSA-ScR (Preferred Reporting Items for Systematic Reviews and Meta-Analysis extension for Scoping Reviews) guidelines as well as the corresponding checklist (supplementary file 1) [26]. The protocol for this scoping review has not been registered or published.

The search strategy was developed within the research team by using the PICO scheme with terms for Population and Intervention being included in the search string [27]. We constructed a structured search string that was adapted to the indexing requirements of each database and applied consistently across all sources.

A comprehensive literature search was performed in June 2023 using the PubMed, Scopus, Cochrane Library, Web of Science Core Collection and CINAHL databases. The complete search strategy is presented in supplementary file 2. An update of this initial search was performed in April 2025.

### Ethical considerations

Because this scoping review focused on previously published literature and did not involve human participants, institutional review board approval was not required.

### Inclusion and exclusion criteria

Based on the research questions and the scope of the review, the following inclusion and exclusion criteria were developed:

Inclusion criteria comprised original research articles investigating digital teaching in undergraduate practical skills training for academic health care professions, published in English or German. No specific time frame was set for the search.

Exclusion criteria included articles that focused on digital teaching in anything other than undergraduate academic health education, did not target practical skills training, were book chapters, reviews, theses, proposals, abstracts, web content, news articles, social media content, reports or protocols, were not relevant to the research questions or were in languages other than English or German. Studies focusing only on clinical decision making or clinical communication skills were excluded. This is because, according to the National Competence-Based Learning Objectives Catalogue for Medicine (NKLM) [22], they represent a distinct competency domain and are not classified as patient-oriented, manual or technical procedures, which form the basis of our aforementioned definition of practical skills.

### Screening process

The screening process consisted of two steps: (1) title and abstract screening and (2) full-text screening based on the above inclusion and exclusion criteria. In addition, we performed a hand-search of the reference lists of the retrieved full texts, especially review articles, to identify further eligible studies that might not have been captured through the database searches.

Screening was performed by two independent investigators (TS and NM for the initial search; TS and MG for the update), who reviewed the studies in the first and second screening steps. Any conflicts were resolved through discussion, involving a third researcher (KF) as an independent supervisor when necessary. After consensus was reached, the respective publications were either included or excluded. Duplicate articles were removed by matching criteria such as title, author and publication date. The screening process was facilitated by the use of the Covidence web tool (Veritas Health Innovation Ltd., Melbourne, Victoria, Australia, 2022) [28].

### Data extraction process

Data extraction was conducted by two reviewers and any disagreements were resolved through discussion, involving the independent supervisor (KF) when necessary. The extracted data included author(s), year, country, aim(s), study population, study design, information about the intervention and control group procedure, a description of the digital intervention used, the practical skill(s) involved, the study outcome(s), the results and the main conclusion(s). In the summary tables, the extracted information was reduced to the core elements - area of application, author and year, intervention, outcome and key results. This reduction was necessary to ensure that the tables remained concise and readable, as including all extracted categories would have resulted in excessively long and difficult-to-interpret tables. More detailed information is provided in the narrative synthesis.

An inductive approach was used to organize the included studies into different categories, i.e. study designs utilized and health care professions addressed. Furthermore, categories were derived from the digital interventions addressed in the included articles. If several types of digital interventions were used in the same article, the article was assigned to the category of the intervention that was used predominantly. The same applies to the classification of articles according to the professions addressed. Articles were assigned to the corresponding profession that was addressed predominantly. The classification of articles was based on a consensus decision between the two researchers (TS and NM for the initial search; TS and MG for the update) and the independent supervisor (KF).

## Results

### Overview of the included studies

**Fig. 1:**
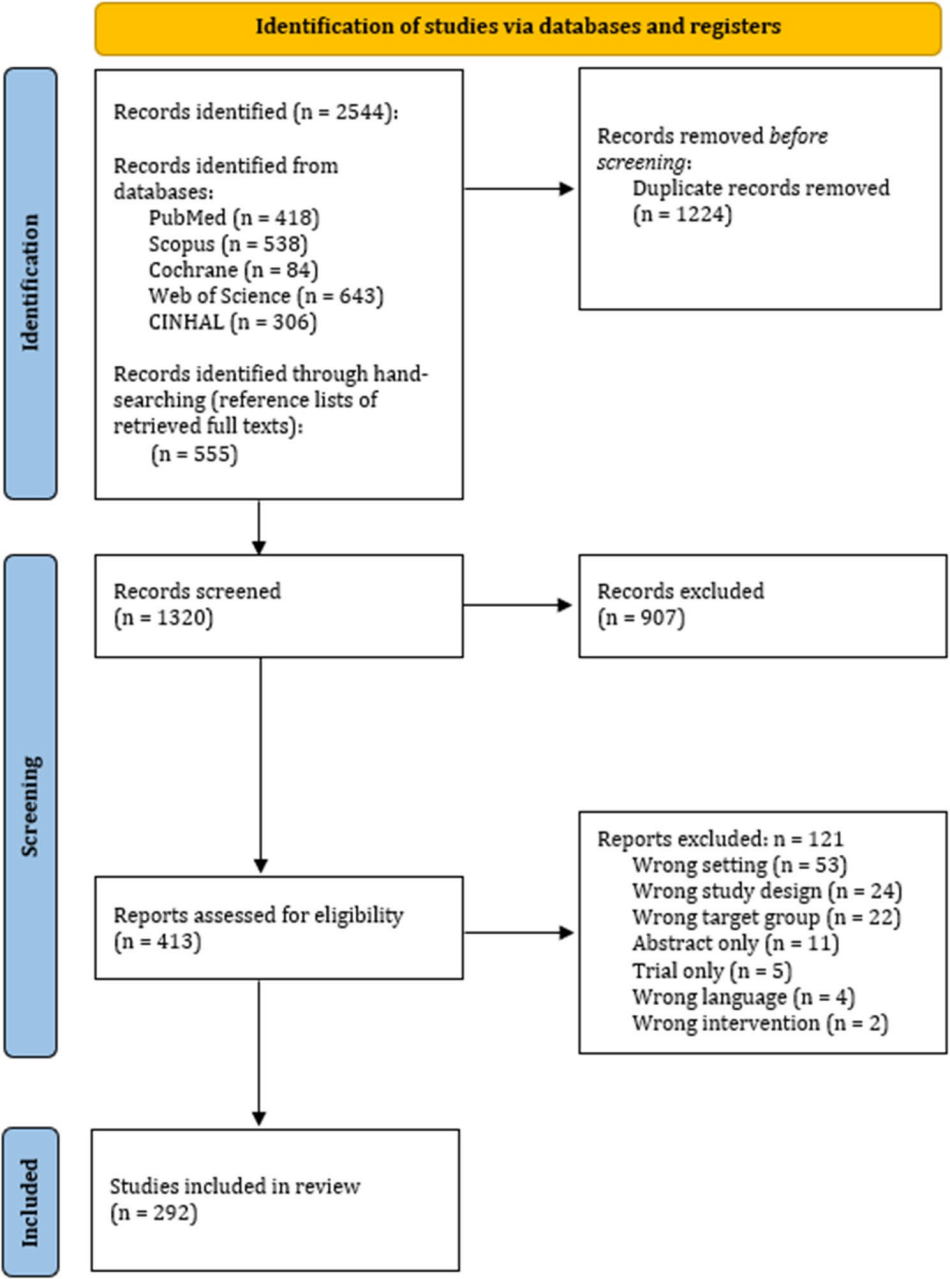
PRISMA flow diagram

The database searches identified a combined total of 2,544 records. After removing 48.1% (1,224/2,544) duplicates, the titles and abstracts of 51.9% (1,320/2,544) of these records were screened. Based on the inclusion and exclusion criteria, the full texts of 16.2% (413/2,544) of the records were reviewed. In total, 11.5% (292/2,544) of the records were included in this scoping review (Fig. 1).

**Fig. 2:**
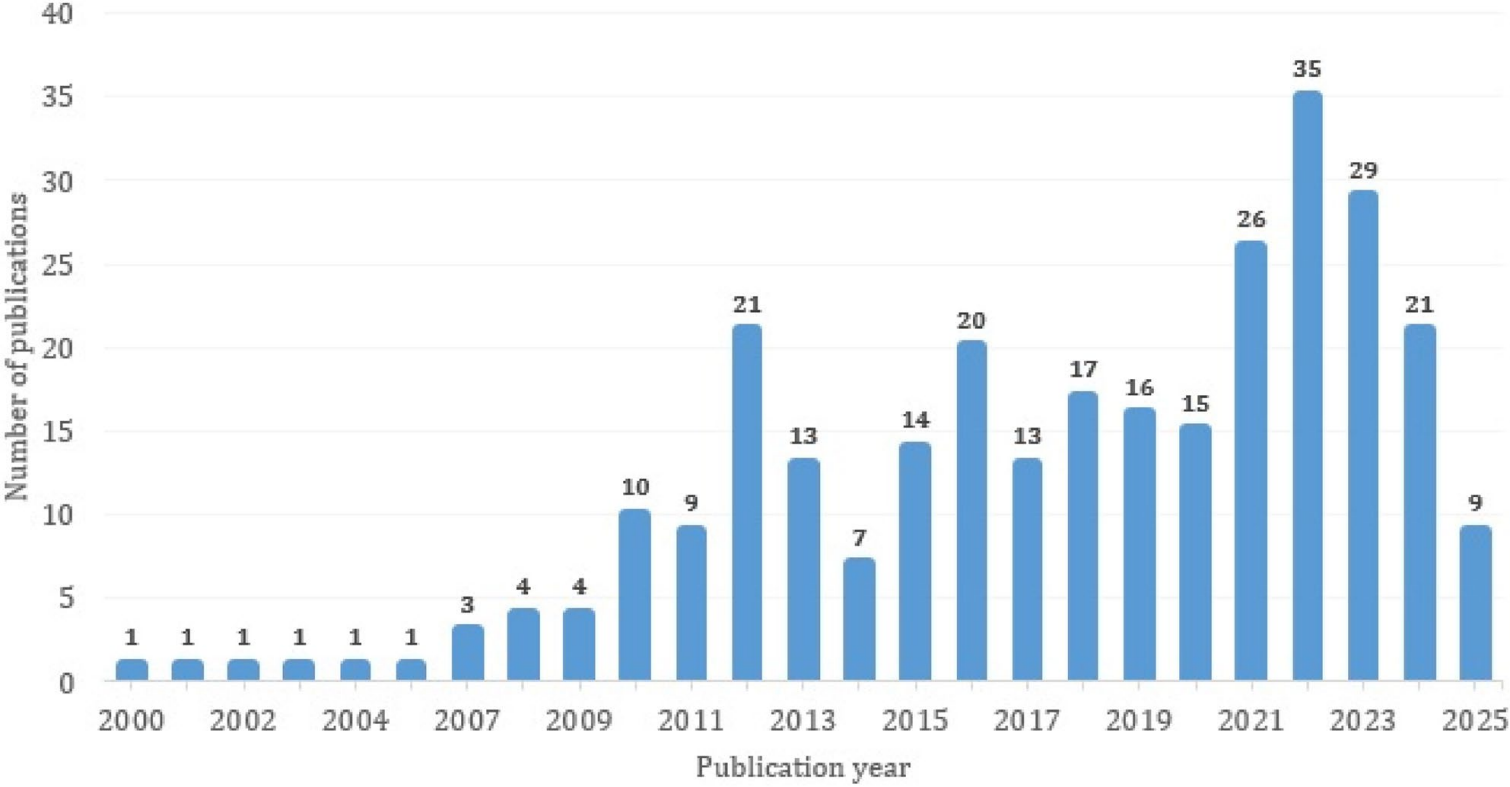
Number of publications per year

Study publication dates spanned from 2000 to 2023, with 41.1% (120/292) published within the last five years (see Fig. 2), aligning with the COVID-19 pandemic.

**Fig. 3:**
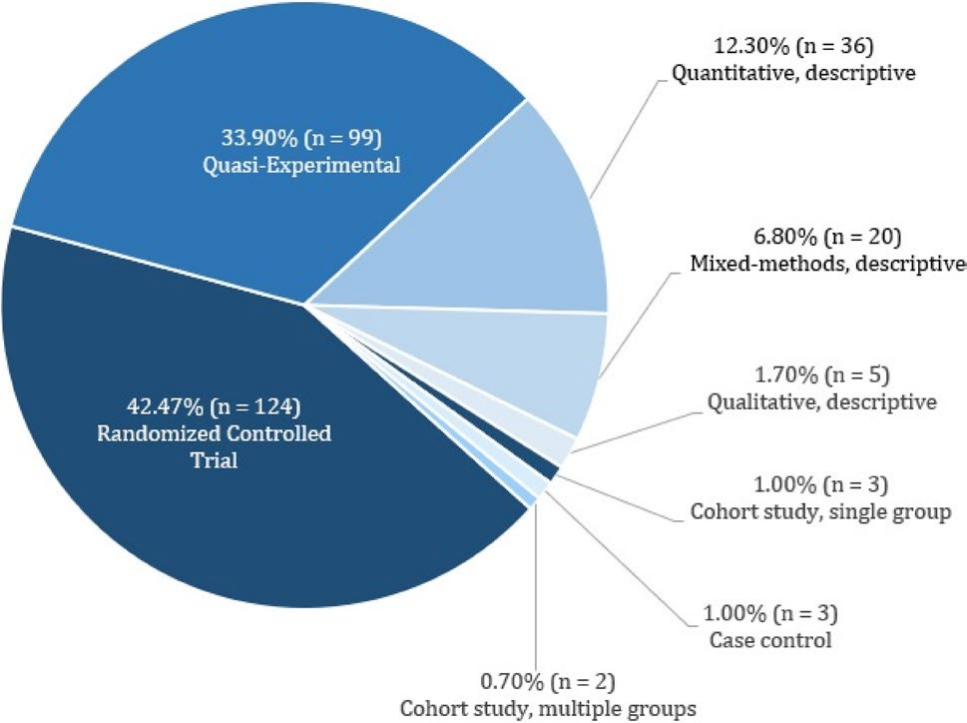
Study designs of the included studies

The most frequently used study design of the included studies was the randomized controlled trial with 42.5% (124/292), followed by the quasi-experimental design with 33.9% (99/292) and the quantitative-descriptive design with 12.3% (36/292). An overview of the study designs used is shown in Fig. 3.

**Fig. 4:**
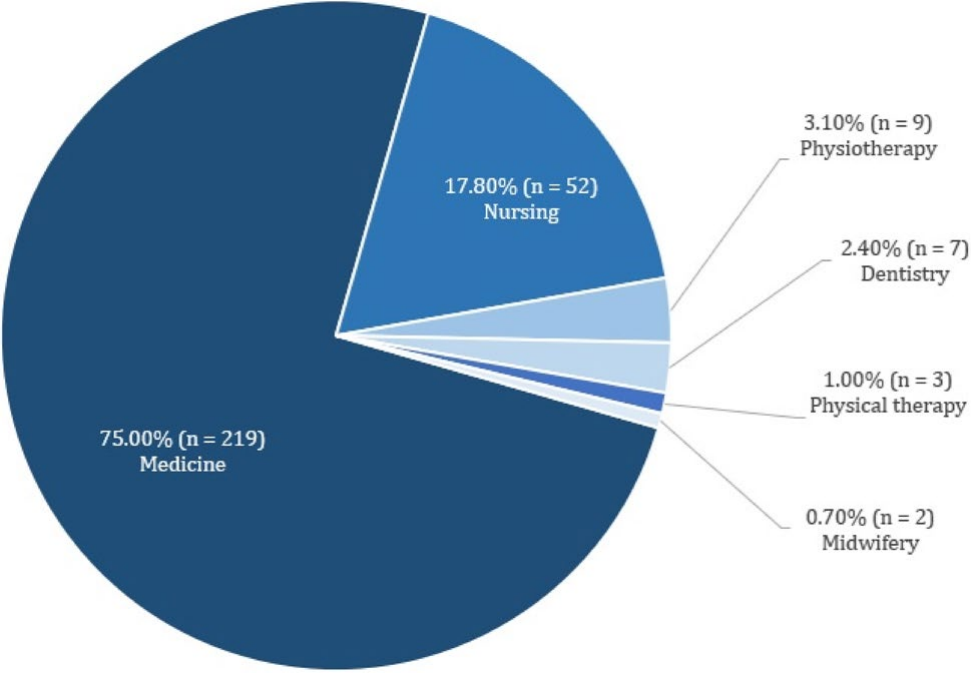
Health professions addressed in the included studies

The most frequently addressed health care profession of the included studies was medicine with 75% (219/292), followed by nursing with 17.80% (52/292) and physiotherapy with 3.10% (9/292). An overview of the health care professions addressed is shown in Fig. 4.

**Fig. 5:**
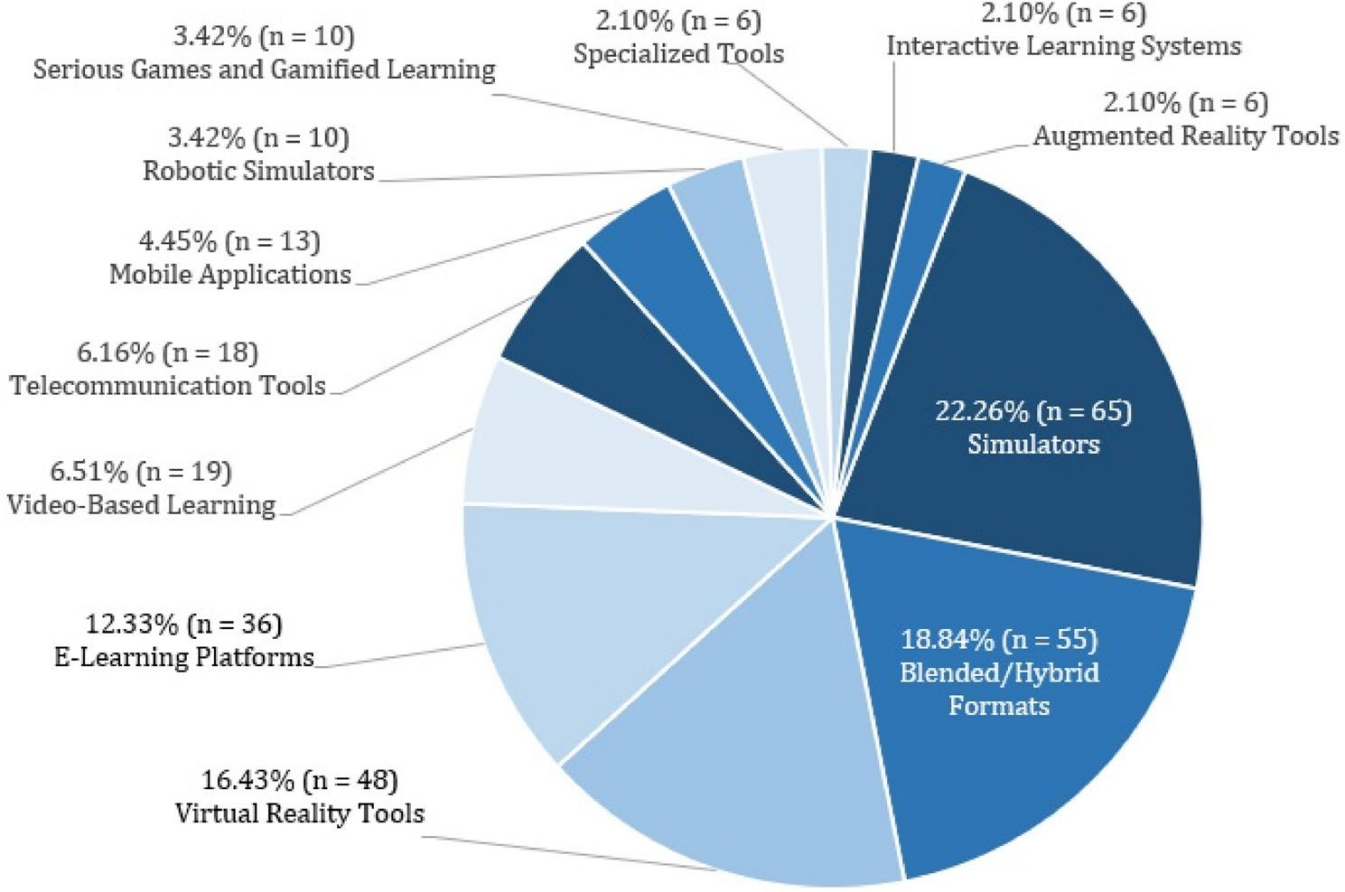
Digital interventions used in the included studies

The most frequently used digital intervention within the included studies was simulators with 22.26% (65/292), followed by blended/hybrid formats with 18.84% (55/292) and VR tools with 16.43% (48/292). An overview of the digital interventions utilized is shown in Fig. 5.

Below, the extracted information from the included studies is presented, categorized according to the digital interventions they employed.

### Virtual reality tools

This category includes virtual reality surgical simulators, VR-based laparoscopic trainers, VR simulators (with and without haptic feedback) and immersive VR environments.

Researchers in the included studies have used VR to support medical training across various procedures, including hands-on surgical skills, diagnostic assessments and infection control. Studies compared VR-based training with traditional methods such as peer-led training or hands-on skills lab sessions or other digital interventions, evaluating outcomes like task completion time, accuracy, procedural knowledge retention, skill transfer and user satisfaction. VR simulators were frequently employed for tasks such as laparoscopy, arthroscopy, bronchoscopy, endoscopy and cardiopulmonary resuscitation. In the identified research, VR tools enabled learners to realistically simulate processes directly related to the corresponding practical skill. Devices used for the virtual reality environment included VR glasses such as the Oculus Rift (Irvine, CA) and Unity-based (San Francisco, CA) platforms.

Studies have shown that VR can significantly enhance the acquisition and retention of clinical and procedural skills. Evidence demonstrates improvements in knowledge, procedural accuracy, error reduction and learner confidence, with many participants reporting greater engagement, realism, and satisfaction compared to conventional training methods. VR has also been shown to accelerate performance, support independent practice, and provide safe, repeatable environments for high-stakes skills. Barriers include reports of simulator or cybersickness, limited tactile feedback compared to real procedures, technical constraints such as object interaction or haptic realism and variability in long-term skill transfer.

Fields of application included arterial blood gas collection, arthroscopy, laparoscopy, basic surgical skills, biopsy needle insertion, bronchoscopy, colonoscopy, echocardiography, cardiopulmonary resuscitation, endoscopy, endovascular surgery, infection control and respiratory care, intramedullary nailing, cholecystectomy, mastoidectomy, nasogastric intubation, ophthalmoscopy, pediatric respiratory assessment and trabeculoplasty. An overview is provided in Table 1.

**Table 1:** Virtual Reality Tools

Area of application	Author(s); year	Intervention	Outcome(s)	Results
Airway management	Botha BS, de Wet L, Botma Y; 2021	Mixed-methods, descriptive; The study explored South African nursing students’ experiences with an immersive VR simulation for managing a virtual patient with an airway obstruction. Using the Oculus Rift, participants practiced history taking, physical and respiratory assessment, interpretation of diagnostic results, and interventions such as oxygen therapy and patient positioning, as well as communication with a physician using SBAR. The study evaluated usability and the potential of VR as a teaching method.	Usability (SUS score), overall satisfaction, task completion rates, Net Promoter Score, time on task, and qualitative feedback on experience.	Overall satisfaction was very high (97.61% in final test), SUS score exceeded 70%, no task failures occurred, and participants completed tasks more quickly than allocated time. Positive feedback focused on realism, engagement, and learning value, with minor issues like occasional dizziness and limited object interaction.
Arterial blood gas collection	Kennedy GAL, Pedram S, Sanzone S; 2023	Quasi-experimental; This study explores the impact of virtual reality-based clinical skills training on reducing medical errors in arterial blood gas collection. The participants in the intervention group received additional virtual reality training, while the control group only received traditional training.	Reduction in human error during the arterial blood gas collection including incorrect positioning, task omissions and procedural deviations.	The virtual reality-trained group had a significantly lower overall error rate compared to the control group.
Arterial blood gas collection	Pedram S, Kennedy G, Sanzone S; 2024	RCT; The study assessed whether VR-based training improves skill acquisition in arterial blood gas (ABG) collection. Participants in the VR group received standard study materials, theory, and a guided VR training session before practical assessment, while the control group received the same preparation but no VR. Both groups completed a knowledge test, observed a live ABG demonstration, and performed the procedure on a simulator, with blinded raters evaluating performance. The VR intervention used a head-mounted display to guide participants through interactive ABG collection tasks, covering hygiene, needle insertion, and blood draw.	Outcomes were knowledge gain, procedural performance, time, errors, assistance needed, and self-reports on motivation, stress, and system acceptance.	The VR group outperformed controls in knowledge, performance, safety, error rates, and independence; the system met 89% of validation criteria.
Arthroscopy	Cychosz CC, Tofte JN, Johnson A, Carender C, Gao Y, Phisitkul P; 2019	Quantitative, descriptive; The study aimed to identify learners’ characteristics that influence skill acquisition, performance and improvement when using an arthroscopy simulator. The training utilized an anatomic virtual reality simulator to teach diagnostic knee arthroscopy.	Information about each participant was collected, including various demographics, sports involvement, hand dominance, specialty interest, 3D video game use and experience assisting in the operating room. Performance on the arthroscopy simulator was measured as time, camera path length and an overall score.	Simulator performance was correlated with how often a trainee played video games. Participants pursuing a non-surgical residency outperformed those interested in a surgical specialty in all measurements, thought this was insignificant. Participants with surgical experience outperformed those without surgical experience, though this difference was not significant. The time factor was significantly influenced by the year of medical school in which the participants were enrolled, with those in higher years performing better than those in lower years. Performance improved significantly for participants who regularly participated in sports requiring hand-eye coordination in comparison to trainees who were not involved in sports.
Arthroscopy	Cychosz CC, Tofte JN, Johnson A, Gao Y, Phisitkul P; 2018	RCT; The study evaluated the effectiveness of self-directed training models using a virtual reality arthroscopy simulator to develop basic arthroscopy skills. Participants engaged with self-guided training modules and then performed arthroscopy on a high-fidelity virtual reality simulator.	Camera path length, time, tibia and femur cartilage damage as well as an overall score were measured.	The intervention group performed superiorly regarding the overall score, camera path length and a greater improvement between baseline and second arthroscopy. Femoral and tibial cartilage damage did not significantly improve between both arthroscopies.
Arthroscopy	Rahm S, Wieser K, Wicki I, Holenstein L, Fucentese SF, Gerber C; 2016	Quasi-experimental; Medical students participated in a standardized four-week training program using a virtual reality knee arthroscopy simulator, practicing tasks such as triangulation, partial meniscectomy and foreign body removal. The simulator provided real-time feedback to assess skill development and learning curves over time.	Time taken to complete tasks, the path lengths of the camera and instruments and the number of foreign bodies removed during the simulation sessions.	Participants showed significant improvement in their overall performance. Right-handedness predicted performance. No other factors, including manual skill or spatial sense, predicted performance.
Arthroscopy and laparoscopy	Akhtar K, Sugand K, Wijendra A, Sarvesvaran M, Sperrin M, Standfield N, Cobb J, Gupte C; 2016	RCT; Intervention groups alternated training programs between laparoscopic and arthroscopic procedures, while control groups focused on a single procedure (laparoscopic cholecystectomy or knee arthroscopy). All groups used virtual reality simulators and repeated their assigned procedures one week later.	Time required, instrument path length, speed	Time required: the 2 intervention groups performed better than the controls. Path length: no difference was found between the groups. Left hand speed: the 2 training groups improved while the control groups did not. Right hand speed: no differences were found between the groups.
Basic life support	Gong Y, Hao Y, Yu Y, Zhang L, Luo N; 2020	RCT; The study assessed whether VR technology enhances learning outcomes, skill retention, and satisfaction in BLS training for medical students compared to traditional instruction. The VR group trained with immersive simulations using head-mounted displays and interactive feedback, while the control group received standard classroom and mannequin-based training. Both groups followed the 2015 AHA BLS guidelines with identical training time. Performance was assessed immediately and at 6 months. Skills included recognizing cardiac arrest, performing chest compressions and artificial ventilation, AED use, and completing the BLS sequence.	BLS knowledge test scores, practical skills assessment scores, skill retention after 6 months, and participant satisfaction.	Immediately post-training, the VR group achieved significantly higher skills assessment scores and satisfaction ratings than the control group, while knowledge scores were similar. At 6 months, the VR group retained skills better than the control group.
Basic life support	Moll-Khosrawi P, Falb A, Pinnschmidt H, Zöllner C, Issleib M; 2022	RCT; The study compared VR-enhanced BLS training with purely web-based instruction during the COVID-19 pandemic. All participants attended a 60-minute online seminar and 120-minute online demonstration, but the intervention group also completed a 35-minute VR BLS session with mannequin-integrated chest compression feedback and virtual modules for ventilation and AED use. Both groups were assessed within three days via a structured clinical examination using the same mannequin. Skills covered included CPR technique, airway management, breathing assessment, AED operation, and adherence to the BLS sequence.	Primary outcome: no-flow time during BLS. Secondary outcome: overall BLS performance via checklist. Tertiary outcome: self-reported learning gain using a comparative self-assessment.	Intervention group had significantly lower no-flow times, fewer penalty points (better performance), and higher subjective learning gains than control, except for the sequence-of-treatment item.
Basic practical dental skills	Ewais W, Obeid A; 2022	RCT; The study compared VR dental simulation training with conventional mannequin-based training for teaching crown preparation skills to dental students. The intervention group practiced with the Simodont^®^ Dental Trainer, while the control group trained with phantom heads under equivalent instruction. Both groups performed baseline and post-training crown preparations on typodont models, assessed with standardized criteria. Skills taught included tooth reduction, finish line placement, taper, and surface smoothness in crown preparation.	Outcomes measured included improvement in preparation scores, time taken to complete the preparation, and student satisfaction with the training method.	Results showed significant improvement in both groups from baseline. The VR group achieved higher improvement in preparation quality scores compared to the control group, while completion times did not differ significantly. Students in the VR group reported greater satisfaction and perceived the training as more engaging and effective.
Basic practical medical skills	Whallett M, Mahesh S, Whittaker J, Crichton A, Ahmed U; 2024	RCT; The study assessed the impact of VR-based training on medical students’ performance during high-fidelity simulations of acutely unwell patient scenarios. The intervention group completed a 20-minute Oxford Medical Simulation VR session using Meta Quest 2 headsets before the high-fidelity simulation, with automated feedback and debriefing. The control group received standard teaching without VR. Skills addressed included clinical management of acutely ill patients and non-technical competencies such as teamwork, communication, and decision-making.	No significant difference between intervention and control groups in non-technical skills (BMS scores) or percentage of critical actions completed; participants reported positive user experience and perceived educational value; cybersickness was reported by 47% of participants.	Non-technical skills: *p* = 0.526, no significant differences; Technical skills: *p* = 0.66, no significant differences; OMS score not significantly correlated with BMS or critical actions; most participants rated VR as immersive, easy to use, and educationally valuable, despite some technical interface limitations and cybersickness symptoms.
Basic practical nursing skills	Chang YM, Lai CL; 2021	Qualitative, descriptive; The study explored nursing students’ experiences using an immersive VR skill learning system for nasogastric tube care. Students practiced with both traditional equipment and the VR system before participating in focus groups. The VR system integrated image design, motion sensors, 3D modeling, and interactive scenarios to train nasogastric tube placement, care techniques, and patient interaction skills.	Thematic perceptions of students regarding VR learning, including ease of practice, learning speed, stress level, environmental impact, and realism.	Five themes identified—convenient to practice but requires adaptation; fast skill learning process; stress-free learning environment; environmentally friendly; lacks a sense of reality.
Basic life support	Ebrahim RM, Anwar K, El-Nagar S; 2024	RCT; The study compared VR-based BLS training with traditional instructor-led, manikin-based teaching for nursing students. The VR group practiced in interactive, immersive environments with real-time feedback, while the control group trained through demonstrations and manikin practice. Both groups learned identical BLS content and were assessed on skills such as cardiac arrest recognition, CPR, AED use, and adherence to BLS protocols.	Knowledge scores, practical BLS performance scores, and self-reported confidence before and after training.	VR group achieved significantly higher post-test knowledge and skill performance scores and reported greater confidence compared to the control group.
Basic surgical skills	Selvander M, Asman P; 2011	Quasi-experimental; A virtual reality-based intraocular surgical simulator configured for cataract surgery tasks was used to evaluate the effect of stereoacuity on surgical performance. Participants completed modules on navigation, forceps manipulation and capsulorhexis creation, receiving standardized instruction and real-time performance feedback.	Simulator scores, time with instruments in the eye and injury metrics. Performance was correlated with stereoacuity levels for each module.	Performance scores for navigation and forceps tasks correlated significantly with stereoacuity. However, there was no significant correlation for the capsulorhexis task, potentially due to the task’s complexity and other compensatory visual cues.
Basic surgical skills	Selvander M, Asman P; 2012	RCT; A virtual reality-based surgical simulator featuring cataract and vitreoretinal surgery modules was used to investigate learning curves and skill transferability between modules. Participants performed repeated tasks in cataract navigation and capsulorhexis, with performance assessed through virtual environment metrics.	Overall performance scores, procedure time, corneal damage and lens injury metrics.	Both groups showed significant improvement over the ten repetitions on each module. However, no skill transfer between modules was observed, as performance on the second module did not benefit from practice on the first.
Biopsy needle insertion	Chellali A, Dumas C, Milleville-Pennel I; 2012	Quasi-experimental; The study evaluated the impact of a virtual learning environment on performing a biopsy task, where participants were taught using verbal instructions, static images, visual strategies with expert demonstrations or expert-led demonstrations. After practicing the technique, participants performed three needle insertion scenarios in a virtual environment designed to teach biopsy skills with haptic feedback.	Task completion time, number of landmarks used, organ penetrations and contact with organs, length of the real insertion path, frequency of missed targets, final distance to the target and insertion segments.	The virtual environment showed to be a good means of learning technical skills such as positioning and the handling of the corresponding tools.
Bronchoscopy	Watson PWL, House S, Hart R, Abbas J, Asthana S, Gaudl SE; 2022	Quasi-experimental; The study evaluated whether virtual reality (VR) simulation could be used to prime procedural bronchoscopy knowledge before hands-on training. The VR group explored a simulated clinical environment to learn procedural steps, while the control group received a traditional pre-brief with printed materials and verbal instructions. Both groups then performed the same medical procedure in a simulated setting. Skills emphasized included procedural sequencing, correct equipment use, and clinical workflow familiarity.	VR preparation increased participant engagement, confidence, and recall of procedural steps, with indications of improved task performance metrics.	VR group showed better procedural recall, slightly faster performance, and fewer errors compared to the control group.
Bronchoscopy, colonoscopy, echocardiography	Omlor AJ, Schwärzel LS, Bewarder M, Casper M, Damm E, Danziger G, Mahfoud F, Rentz K, Sester U, Bals R, Lepper PM; 2022	Quasi-experimental; This study compared immersive and non-immersive virtual reality videos as substitutes for in-hospital teaching, focusing on clinical procedures like bronchoscopy, colonoscopy, echocardiography and dialysis. Participants observed 3D clinical procedure videos either through VR headsets or in 2D on standard devices, followed by knowledge and self-assessment tests.	Students’ scores on a learning-success test and their self-assessment of expertise and satisfaction with the course.	No significant difference in learning success between the immersive and non-immersive groups was found. However, students in the immersive virtual reality group rated the course more positively in terms of clarity, learning atmosphere and overall satisfaction. They were also more likely to recommend the course and felt that virtual reality improved their understanding of the procedures.
Cardiopulmonary resuscitation	Creutzfeldt J, Hedman L, Felländer-Tsai L; 2012	Quasi-experimental; The study examined students’ retention of knowledge and skill after training in cardiopulmonary resuscitation (CPR) using a multiplayer virtual world teaching format. Participants controlled avatars in a virtual environment, performed team tasks and were assessed through pre- and post-tests on CPR knowledge and skills using a full-scale simulator.	A basic life support knowledge test. Performance of cardiopulmonary resuscitation was recorded and analyzed along factors such as compression cycles frequencies, time for taking care of the cardiac arrest victim and adherence to guidelines.	The intervention group showed a greater knowledge than the control group. Adherence to guidelines was higher in the intervention group than in the control group. Participants in the intervention group also showed a lower number of incorrect frequencies for chest compression cycles.
Cardiopulmonary resuscitation	Creutzfeldt J, Hedmann L, Medin C, Heinrichs L, Felländer-Tsai Li; 2010	Quantitative, descriptive; The study aimed to determine whether a serious game environment using virtual world avatars affects medical students’ retention of CPR-related knowledge and their subjective experience. The intervention utilized a massively multiplayer virtual world designed for team-based training in cardiopulmonary resuscitation (CPR).	Self-efficacy, engagement modes, coping strategies, concentration and mental strain were measured as well as knowledge about the procedure.	The majority of subjects were satisfied with the training method and estimated their knowledge gain as high. Measured procedural knowledge retention decreased. Several students commented that the scenarios could be more difficult and complex and that the virtual environment could be richer. Self-efficacy and perceived concentration increased, while mental effort was found to be reduced.
Colonoscopy	Snyder CW, Vandromme MJ, Tyra SL, Hawn MT; 2010	RCT; Colonoscopy skills were practiced using a virtual reality simulator, with one group receiving expert proctoring and real-time feedback, while the other group trained independently with automated feedback. Skill retention was assessed after 4.5 months, following initial proficiency training and live surgery lab performance.	A proficiency score based on specific criteria (e.g. visualization, time, errors), retention of skills at a later time point and comparison of error frequency and skill components between groups.	Both groups retained their skills, with no significant difference between baseline and retention scores. Skills related to speed and efficiency were well-retained, while error avoidance skills were less reliably retained. The independent group showed slightly better performance at retention testing, though the difference was minor.
Encoscopy	Tanoue K, Ieiri S, Konishi K, Yasunaga T, Okazaki K, Yamaguchi S, Yoshida D, Kakeji Y, Hashizume M; 2008	RCT; Training on the virtual reality simulator, participants practiced endoscopic suturing and intracorporeal knot tying for two hours daily over two days. Watching only an educational video on suturing and knot tying, the control group did not participate in hands-on training.	Time taken to complete the suturing and knot tying tasks as well as the number of errors and efficiency in task execution.	Both the simulator and the box trainer group showed significant improvements in task completion time and accuracy compared to the control group. However, the box trainer group showed a higher rate of errors, while the simulator group demonstrated smoother and safer instrument handling.
Endovascular surgery	Maertens H, Aggarwal R, Desender L, Vermassen F, Van Herzeele I; 2015	Quasi-experimental; Participants completed e-learning modules and used a virtual reality device to practice endovascular procedures, with a focus on cognitive, technical and human factor skills. The performance of medical students was compared to expert vascular surgeons’ performance using the same training format, involving tasks related to treating iliac and superficial femoral artery stenoses.	Procedure time, use of fluoroscopy, contrast agent, number of catheters used and simulation-based metrics.	Expert surgeons consistently outperformed medical students in all assessed parameter. An improvement in students’ performance over time was found and students successfully completed the program within 69 to 77 days.
Infection control and respiratory care	Yu M, Yang MR; 2022	Quasi-experimental; The experimental group engaged in a virtual reality infection control simulation, which included prebriefing with theoretical instruction, a hands-on simulation of donning and doffing personal protective equipment, respiratory care for pediatric COVID-19 patients and a debriefing session for feedback. The control group followed their standard curriculum without simulation training and both groups completed pretests and posttests assessing knowledge, performance and self-efficacy.	Personal protective equipment knowledge, infection control performance, self-efficacy scores and realism and satisfaction scores.	The intervention group significantly outperformed the control group in personal protective equipment knowledge, infection control performance and self-efficacy. High scores were reported for realism and learner satisfaction in the intervention group.
Intramedullary nailing	Blumstein G, Zukotynski B, Cevallos N, Ishmael C, Zoller S, Burke Z, Clarkson S, Park H, Bernthal N, SooHoo NF; 2020	RCT; A virtual reality simulation tool was assessed for teaching the surgical technique of tibial shaft fracture intramedullary nailing. Participants trained using either the VR method or a standard guide and were observed performing the procedure in a simulated setting immediately after training.	Evaluation was done by a blinded attending surgeon using a procedure-specific checklist and a 5-point global assessment scale.	Assessment scores were higher for the VR compared to the control group.
Laparoscopy	Lesch H, Johnson E, Peter J, Cendan JC; 2020	RCT; This study involves both a virtual reality simulator with haptic feedback and a 3D rendering tool for laparoscopic surgery, as well as a video-based learning tool to teach laparoscopic procedures, specifically appendectomy and cholecystectomy.	Knowledge scores and students’ confidence in reproducing the procedural steps.	The virtual reality simulator was found to enhance students’ confidence in reproducing the procedural steps more than the video-based learning format, especially for the cholecystectomy. No significant differences between modalities was found regarding the knowledge acquisition on appendectomy. Students in the video-based learning format performed better regarding cholecystectomy.
Laparoscopy	Mackay S, Morgan P, Datta V, Chang A, Darzi A; 2002	RCT; This study investigated the effects of different practice methods—blocked and distributed—on the acquisition of laparoscopic surgical skills using a virtual reality-based training system. Participants practiced laparoscopic skills on the virtual reality simulator, performing the “transfer place” task, which involved grasping and transferring objects within a virtual environment under three different conditions.	Overall performance score, task completion time, errors and path length economy. Also, skill retention was assessed.	Distributed practice (group B) led to significantly better performance on the retention test than massed practice (group A). Group C, which had less total simulator time, did not show significant improvements compared to group A.
Laparoscopy	Nemani A, Ahn W, Cooper C, Schwaitzberg S, De S; 2018	RCT; This research examined the validation of a virtual reality-based pattern cutting simulator and evaluated the transfer of surgical skills from simulation environments to cadaveric models. Participants performed pattern cutting tasks on both virtual reality and conventional simulators, followed by retention trials and a transfer task on cadaveric tissue.	Task performance scores, task completion times and learning curves. Transfer of skills was assessed through task performance on cadaveric tissue.	Both conventional simulator and virtual reality-based simulator groups outperformed the control group. Both groups performed significantly better than the control group on the cadaveric tissue task.
Laparoscopy	Nickel F, Brzoska JA, Gondan M, Rangnick HM, Chu J, Kenngott HG, Linke GR, Kadmon M, Fischer L, Müller-Stich BP; 2015	RCT; This research compared the effectiveness of a virtual reality-driven simulator and a blended learning format (combining box trainers and e-learning) for teaching laparoscopic skills to medical students. Participants trained using either method before completing a post-test, which included a knowledge assessment and a simulated laparoscopic cholecystectomy on a cadaveric organ.	Overall performance scores were taken. Secondary outcomes included the general and specific technical skills subscores, operation time, completion rate of operations within 80 min and performance on a knowledge test.	Both groups showed equal performance on the overall performance scores. The virtual reality group completed the procedure faster and more frequently within 80 min. The blended learning group scored higher on the knowledge test.
Laparoscopy	Onishi S, Ikee T, Murakami M, Yano K, Harumatsu T, Baba T, Yamada K, Yamada W, Masuya R, Machigashira S, Nakame K, Mukai M, Kaji T, Ieiri S; 2019	RCT; A comparison was made between three short-term endoscopic surgical skill training methods—video-based, expert-led and virtual reality simulator training—for teaching laparoscopic suturing and knot-tying skills. Participants practiced these skills using a virtual reality-based simulator, while control groups received either video-based or expert-led instruction.	Time to complete the task, suturing balance, suturing intervals, total path length of the forceps, average velocity, acceleration and the number of tissue injuries during the task.	No significant differences in the primary evaluation parameters between the groups were found. The virtual group had significantly faster acceleration of the assistant forceps compared to the video group.
Laparoscopy	Salkini MW, Doarn CR, Kiehl N, Broderick TJ, Donovan JF, Gaitonde K; 2010	RCT; The study evaluates the impact of haptic feedback in a virtual reality simulation environment. Participants performed laparoscopic tasks using a VR simulator, with one group experiencing haptic feedback and the other without it.	Accuracy, economy of movement and speed of hand movement.	No significant differences in accuracy, economy of movement or speed were found between the two groups. Video gamers in both groups showed better hand economy and faster hand movements.
Laparoscopy	Sánchez-Peralta LF, Sánchez-Margallo FM, Moyano-Cuevas JL, Pagador JB, Enciso S, Gómez-Aguilera EJ, Usón-Gargallo J; 2012	Quantitative, descriptive; To evaluate how effectively a virtual reality simulator supports the acquisition of basic laparoscopic psychomotor skills, participants engaged in training on five tasks, including coordination, navigation and grasping, using the VR simulator.	Total time, partial time, fulfillment percentage, instrument efficiency and non-coordination moments.	Significant improvements were seen in the majority of the metrics by the final session.
Laparoscopy	Strandbygaard J, Bjerrum F, Maagard M, Winkel P, Larsen CR, Ringsted C, Gluud C, Grantcharov T, Ottesen B, Sorensen JL; 2013	RCT; Participants in the intervention group received standardized instructor feedback during virtual training sessions, with up to three feedback sessions provided. The control group practiced independently on the virtual reality laparoscopic simulator without instructor feedback, training until they achieved a predefined proficiency level.	Time and number of repetitions required to reach the expert proficiency level and performance scores provided by the simulator upon reaching proficiency.	The intervention group required significantly less time and fewer repetitions to reach the proficiency level than the no-feedback group. The no-feedback group achieved a higher final performance score.
Laparoscopy and cholecystectomy	Nomura T, Mamada Y, Nakamura Y, Matsutani T, Hagiwara N, Fujita I, Mizuguchi Y, Fujikura T, Miyashita M, Uchida E; 2015	Quasi-experimental; This research evaluated the improvement of laparoscopic skills in medical students after training with a virtual reality-driven simulator. Participants completed various laparoscopic tasks such as grasping, cutting, suturing and performing cholecystectomy, with pre- and post-tests conducted using an augmented reality-based object-positioning module.	Execution time, instrument path length and economy of movement.	Significant improvements in execution time, path length and economy of motion were found after virtual reality simulation training.
Laparoscopy and cholecystectomy	Yang C, Kalinitschenko U, Helmert JR, Weitz J, Reissfelder C, mees ST; 2018	RCT; Participants were divided into two groups: one first trained on laparoscopic appendectomy before transitioning to laparoscopic cholecystectomy, while the other completed the tasks in the reverse order. Both groups underwent standardized basic laparoscopic training before performing a full cholecystectomy on a virtual reality simulator, assessed based on time, safety and efficiency.	Time taken to complete tasks, safety parameters, economy parameters and subjective physical and mental load during tasks.	Group 1 demonstrated more economical movements during cholecystectomy compared to Group 2. No significant differences were found in time or safety parameters between the groups. No significant reduction in physical or mental load was observed.
Laparoscopy and endoscopy	Snyder CW, Vandromme MJ, Tyra SL, Porterfield Jr. JR, Clements RH, Hawn MT; 2011	RCT; Different training methods using a virtual reality simulator were compared, with some participants receiving expert feedback and others relying on automated feedback. Additionally, participants were exposed to either live or video-based observational learning before performing minimally invasive tasks on an animal model.	Time to complete each surgical task and the overall task completion rates in the live animal model.	No significant differences were found between proctored and independent training groups regarding task completion. Participants who observed the live procedure before performing it had a significantly higher likelihood of successful task completion compared to those who only watched the instructional video.
Mastoidectomy	Andersen SAW, Guldager M, Mikkelsen PT, Sorensen MS; 2019	Cohort study, multiple groups; Twenty-nine medical students trained for temporal bone surgery using the Visible Ear Simulator, a virtual reality simulator for anatomical mastoidectomy. One group performed structured self-assessments after each procedure, while the other group did not.	A modified version of the Welling Scale was used to evaluate performances. Furthermore, various measurements were recorded by the simulator, such as time used or outcome quality.	The VR-based simulator training group using structured self-assessment performed superiorly than the control cohort.
Mastoidectomy	Andersen SAW, Konge L, Cayé-Thomasen P, Sorensen MS ; 2016	Quasi-experimental; Forty-three medical students trained in mastoidectomy skills using a VR-driven temporal bone surgical simulator. One group practiced with the simulator on several occasions, while the other group practiced for a single day.	Surgical skill performance as assessed by a modified Welling Scale.	Learning curves were superior for the intervention group compared to the control group.
Mastoidectomy	Andersen SAW, Konge L, Cayé-Thomasen P, Sorensen MS; 2016	Cohort study, multiple groups; Medical students trained in mastoidectomy skills using the Visible Ear Simulator, a VR-driven surgical simulator. After completing 12 training repetitions, distributed over multiple sessions or in a single day, participants performed two virtual mastoidectomies to assess their skills.	Performance was rated by two masked experts using a modified version of the Welling Scale. Reaction time was also measured while performing a secondary task provided by the simulator.	Mastoidectomy skills were largely maintained after 3 months in both practice groups, but the one-day practice group took longer to complete the task. Their performance improved significantly between the first and second retention procedures, suggesting less skill consolidation compared to the distributed practice group.
Nasogastric intubation	Aluthge CLP, Weerasinghe TA, Imeshika KAS, Sandaruwan KD; 2022	Mixed-methods, descriptive; Nursing students learned nasogastric intubation using a virtual reality-based application. The intervention focused on enhancing practical nursing skills through virtual reality technology.	Satisfaction with and usability of the VR-based learning format	Participants rated the VR learning method as satisfactory in terms of ease of use, clarity of instructions, interactivity, adaptive feedback and learning support.
Neurosurgery	Reinhold M, Asal C, Driesen T, Roch J, Jäckle K, Borgmann S, Lehmann W; 2024	RCT; The study evaluated a VR-based 3D training platform for teaching complex spinal surgery skills and compared it to traditional teaching. Medical students in the VR group attended a 45-minute VR lecture with 3D anatomy models, imaging, and simulations, followed by 45 min of supervised VR practice in a virtual OR. The control group received the same lecture content face-to-face with written self-study. Both groups were tested on pedicle screw placement in a simulated OR using sawbone models and real instruments. Skills taught included lumbar spinal anatomy, procedural steps, and practical screw placement technique.	Immediate procedural knowledge retention measured by MC test, practical skill performance assessed via DOPS, and self-assessment questionnaires on satisfaction, confidence, and perceived preparedness.	Knowledge quiz scores were similar between groups (control: 69%, VR: 66%, *p* = 0.692); practical DOPS performance showed no statistically significant difference, though more VR participants achieved “good” to “very good” ratings (10 vs. 3 in control, *p* = 0.134); VR group rated innovation higher but reported less self-study time; some participants experienced simulator sickness.
Operating room preparation	Grunewald A, Schmidt R, Sayn L, Gießer C, Eiler TJ, Schmuecker V, Braun V, Brueck R; 2021	Quantitative, descriptive; The study developed and evaluated Tersus, a VR training application designed to reduce medical students’ anxiety and improve readiness for their first operating room experience. The application allowed students to practice OR preparation tasks including sterile gowning and gloving, surgical hand disinfection, avoiding contamination, preparatory washing of a surgical site, and correct OR behavior. Built in Unity with Leap Motion and HTC Vive controllers, it incorporated gamification and self-guided learning features. The VR training was integrated into a hybrid course, aiming to enhance confidence and procedural competence before real OR exposure.	Outcomes were student perceptions of immersion, familiarity with OR procedures, reduction of anxiety, and understanding of preparation steps, assessed via the Igroup Presence Questionnaire and additional items.	Results showed a highly positive evaluation regarding immersion, guidance, and realism; all students reported feeling better prepared for the OR, understood the sequence of surgical hand disinfection, and could transfer this knowledge to real-world practice; minor motion sickness was noted in some participants.
Ophthalmoscopy	Wilson AS, O’Connor J, Taylor L, Carruthers D; 2017	Quantitative, descriptive; Trainees engaged with a virtual reality-based ophthalmoscopy training application, completing tasks related to retinal examination, pathology identification and a knowledge assessment. The application was developed using Unity and optimized for smartphones and virtual reality headsets to enhance accessibility.	Students’ perceived improvement in understanding ophthalmoscopy, the ability to identify eye landmarks, recognition of abnormalities and their confidence in performing the tasks in real-life scenarios.	The virtual reality application was well-received, with students reporting improved understanding of ophthalmoscopy and confidence in performing eye examinations.
Pediatric respiratory assessment	Zackoff MW, Real FJ, Cruse B, Davis D, Klein M; 2019	Quantitative, descriptive; All participants completed the immersive virtual reality curriculum using Oculus Rift headsets to engage in simulated pediatric respiratory assessment scenarios. The VR system provided interactive feedback and students were guided by a virtual preceptor avatar while evaluating respiratory statuses such as no distress, respiratory distress and impending respiratory failure.	Students’ perceived effectiveness of the virtual reality training compared to other methods, levels of immersion and realism in the virtual reality environment and engagement and its impact on future clinical decision-making.	85% of students reported a strong sense of presence in the virtual reality environment. 97% rated the scenarios as clinically accurate and 100% felt the training reinforced key learning objectives. Virtual reality was perceived as more effective than reading, didactic teaching and low-fidelity mannequins and equally effective or superior to high-fidelity mannequins and standardized patients.
Sonography	Khoo C, Sharma S, Jefree RA, Chee D, Koh ZN, Lee EXY, Loh NHW, Ashokka B, Paranjothy S; 2025	RCT; The study compared self-directed VR training with traditional physician-led instruction for teaching cardiac POCUS to medical students. Both groups first completed an e-learning module, then either trained with DeepScope VR on Oculus Quest 2 or with physician-led demonstrations and supervised practice using a HeartWorks simulator. Outcomes included knowledge (MCQs) and practical skills assessed immediately after training and at 1-month follow-up. The VR system guided students through interactive modules to achieve > 90% accuracy in acquiring the four standard cardiac views, focusing on probe handling, image optimization, and anatomical identification.	Primary outcomes: total MCQ and practical skills scores post-training and at 1 month. Secondary outcomes: individual view scores and time to image acquisition.	Immediately post-training, PL group had significantly greater improvement in MCQ scores and higher practical skills scores. At 1 month, differences in knowledge and skills retention were not statistically significant, with VR group scoring higher in practical skills for some views.
Surgical suturing	Cardona-Rivera JL, Alvarez-Rodriguez F, Cardona-Reyes H, Muñoz-Arteaga; 2023	Mixed-methods, descriptive; The study developed and evaluated a VR environment for medical students to practice continuous simple suturing techniques. Using the Oculus Quest 2 with a Unity3D-based simulation, participants practiced in a virtual operating room with animated tools and tissue. The intervention aimed to assess the usefulness and effectiveness of VR for teaching this essential surgical skill.	User experience was evaluated using the User Experience Questionnaire (UEQ), which measured six dimensions: attractiveness, perspicuity, efficiency, dependability, stimulation, and novelty	All six UEQ dimensions had positive mean scores above 1. The environment was rated especially well in attractiveness (mean: 2.29), stimulation (2.07), and efficiency (2.07), indicating strong perceived quality and usefulness. Students found it realistic and valuable for practice
Surgical suturing	Peters P, Lemos M, Bönsch A, Ooms M, Ulbrich M, Rashad A, Krause F, Lipprandt M, Kuhlen TW, Röhrig R, Hölzle, Puladi B; 2023	RCT; The study compared an immersive VR-based suturing course with an e-learning course and a tutor-led course. Participants in the VR group watched a stereoscopic 180° first-person instructional video on Oculus Quest 2 before practicing suturing independently, while control groups used either e-learning or tutor-led instruction. All groups completed a theoretical session, practice time, and pre- and post-suturing assessments, evaluated by blinded raters. The study measured improvement in Global Rating Scores, self-assessed progress, number of sutures performed, and associations with visual-spatial ability.	Primary: improvement in Global Rating Score (ΔGRS) from pre- to posttest. Secondary: student self-assessment, number of sutures performed, correlations with visual-spatial ability and optical flow from videos.	All groups showed skill improvement. ΔGRS was similar across groups (HMD: 14.1, Tutor: 12.7, E-learning: 12.4). The number of sutures performed was a strong predictor of improvement; visual-spatial ability showed no correlation. The HMD group reported significantly better learning experience than the e-learning group.
Trabeculoplasty	Alwadani F, Morsi MS; 2012	RCT; Forty-seven medical students participated in a randomized controlled trial to evaluate a virtual reality training method for argon laser trabeculoplasty skills using the PixEye simulator. All students attended a detailed PowerPoint presentation, followed by either virtual reality or traditional training, before performing the procedure.	Missing the exact location with the laser, overtreatment, undertreatment and inadvertent laser shots to iris and cornea	Participants in the virtual reality-based intervention group showed significant improvements across all measurements

### Augmented reality tools

This category includes AR instructional systems, AR support for applied methods and AR for procedural skills training courses.

Researchers in the included studies have used AR to enhance training in areas such as basic practical nursing skills, intravenous cannulation and suturing. AR interventions involved providing real-time visual and audio feedback, interactive step-by-step guidance or augmented overlays using devices like smart glasses or the Microsoft HoloLens (Redmond, WA).

Studies have shown that AR can improve procedural accuracy, user satisfaction and learning efficiency, although technical issues and compatibility problems can be barriers to successful training.

Fields of application included basic practical nursing skills, suturing and intravenous cannulation. A summary of the information can be found in Table 2.

**Table 2:** Augmented Reality Tools

Area of application	Author(s); year	Intervention	Outcome(s)	Results
Basic practical nursing skills	Garrett BM, Jackson C, Wilson B; 2015	Mixed-methods, descriptive; Augmented reality technology was used to support nursing students’ learning of daily nursing skills such as equipment use, clinical hand-washing and respiratory auscultation. Participants used this technology to explore resources within the daily nursing environment.	Factors such as organization, technical functionality and usability of the augmented reality software.	Students expressed comfort with the technology and praised the usefulness of the software in supporting self-directed learning of nursing skills. Technical issues and incompatibility problems were barriers and were associated with a negative learning process.
Basic practical nursing skills	Kim SK, Lee Y, Yoon H, Choi J; 2021	Quantitative, descriptive; The study developed and evaluated a smart glass–based training program for undergraduate nursing students to support self-practice of clinical skills. Using Vuzix Blade smart glasses with extended reality image guides, students practiced blood transfusion and intradermal injection during a 2-hour lab session. The study assessed the usability and feasibility of smart glasses as an educational tool for independent nursing skill practice.	Usability, learning satisfaction, perceived competency in core nursing skills, performance accuracy, and time to completion.	Significant improvement in perceived competency for both skills (*P*<0.001), high usability and satisfaction ratings, correlations between frequency of smart glass use and both increased satisfaction and reduced performance time; reported device-related discomforts (small screen, heaviness, fogging, touch sensor issues).
Bladder Catheterization	Schoeb DS, Schwarz J, Hein S, Schlager D, Pohlmann PF, Frankenschmidt A, Gratzke C, Miernik A; 2020	RCT; The study evaluated a mixed reality guidance system (Microsoft HoloLens) for teaching bladder catheter placement compared to conventional instructor-led training. Students in the MR group practiced on a male catheterization model while receiving step-by-step visual and audio instructions through the headset, with no direct instructor guidance beyond technical support. The control group received the same standardized instructions from a human instructor. Both groups performed the procedure on identical models using standardized materials, focusing on sterile and accurate catheter insertion.	Primary outcome: OSCE performance score after 3 days; secondary outcomes: self-evaluations of skill, knowledge, and confidence; MR group also evaluated usability via SUS and NASA-TLX.	MR group scored significantly higher in OSCE (21.49 ± 2.27) than control group (19.96 ± 2.42, *p* = 0.00); self-evaluations improved in both groups without significant difference; MR system usability rated low (SUS score 56.6), with some technical challenges and higher reported emotional stress.
Intravenous cannulation	Wolf J, Wolfer V, Halbe M, Maisano F, Lohmeyer Q, Meboldt M; 2021	Quasi-experimental; Trainees followed step-by-step augmented reality-based instructions on the Microsoft HoloLens 2, which included text, images, videos and 3D models to guide extracorporeal membrane oxygenation (ECMO) cannulation. In contrast, the control group used conventional printed instructions supplemented with images and videos on a desktop computer before performing the procedure on a simulator.	Training time, error rates and user experience.	The augmented reality instructions resulted in slightly longer training times but significantly fewer errors in more complex tasks, especially knowledge-related errors. Participants rated augmented reality instructions higher on user experience dimensions like clarity, information speed and stimulation.
Physical examination	Moro C, Bu D, Gadgil A, Wright G, Jones CJ; 2023	Quantitative, descriptive; The study explored medical students’ perceptions of using augmented reality lung models as an alternative to physical pathology specimens. Participants interacted for about 10 min with a tablet-based AR lung model developed in 3ds Max before completing a survey. The AR tool was designed to support visualization of anatomical structures and pathological changes in pathology education, and the study assessed its perceived usefulness, learning impact, and student preferences.	Student perceptions of AR versus physical specimens, preferences for learning modalities, and perceived impact on learning and clinical preparedness.	Most students opposed replacing physical specimens with AR (58.15% disagreed), over half felt replacement would not be beneficial (55.4%), and 66.2% believed absence of physical specimens would negatively impact knowledge. Many valued AR for viewing structures from more angles and for remote access. 89.2% preferred having both modalities.
Surgical suturing	Neves Lopes V, Dantas I, Barbosa JP, Barbosa J; 2022	RCT; This research evaluated the effectiveness of a telestration teaching method using augmented reality via smart glasses, compared to traditional in-person teaching for basic surgical skills. Participants received real-time visual and audio feedback from a remote instructor through AR technology while practicing suturing techniques.	Time required to complete each suture during both mentored and independent performance. Quality of sutures assessed using a modified objective structured assessment of technical skills. Students’ evaluation of the course through a Likert scale questionnaire, including self-evaluation and satisfaction.	Students in the telestration group completed tasks independently faster than those in the traditional group, with significant differences for the cruciate mattress and simple continuous sutures. No statistically significant differences were found in objective structured assessment of technical skills scores or suture tension scores between the groups. Both groups rated the quality of teaching and materials highly.

### Simulators

This category includes high-fidelity simulators, low-fidelity simulators, laparoscopic, arthroscopic and endoscopic simulators as well as the use of virtual patients and intravenous simulators.

Researchers in the included studies have used simulators extensively to improve training across procedures, such as laparoscopic surgery and cardiopulmonary resuscitation. Simulators ranged from high-fidelity devices to low-cost, task-specific models, often assessed for skill acquisition, task completion time, accuracy and realism.

Studies generally reported improved learning outcomes, enhanced practical skills and increased confidence among participants, although some showed mixed results when comparing simulators to live demonstrations, small-group tutorials or hands-on skills lab sessions.

Fields of application included apicoectomy, arthroscopy, basic practical nursing skills, basic surgical skills, basic suturing skills, blood sample examination, bronchoscopy, cardiopulmonary resuscitation, catheter ablation, cholecystectomy, cricothyrotomy, endoscopy, endovascular surgery, intravenous cannulation, intravenous catheterization, laparoscopy, laparoscopic bead transfer and suturing, lumbar puncture, robot-assisted laparoscopy, mastoidectomy, medication administration, neonatal resuscitation, otolaryngologic surgery, otoscopy, physical examination, physical patient treatment, pulmonary artery catheterization, sonography, surgery preparation and venipuncture. Table 3 provides an overview.

**Table 3:** Simulators

Area of application	Author(s); year	Intervention	Outcome(s)	Results
Apicoectomy	Buchbender M, Maser M, Neukam FW, Kesting MR, Attia S, Schmitt CM; 2021	Quasi-experimental; The study evaluated the effectiveness of the oral surgery simulator “Kobra” in teaching dental surgery techniques, including an apicoectomy of an upper lateral incisor and the removal of an impacted lower wisdom tooth. Participants practiced both procedures using the Kobra simulator to assess their skill acquisition.	Subjective parameters (questionnaires with a scale from 1–5): improvement of practical skills, comparison between conventional training and Kobra simulation and implementation of simulation-based teaching. Objective parameters (measured while performing the Kobra simulation): removal of bone, removal of tooth substance and removal of soft tissue in mm³.	In comparison to the Kobra simulation, the conventional training method with plastic models was still favored by participants. Dentists performed better than students, though the difference was not significant.
Arthroscopy	Banaszek D, You D, Chang J, Pickell M, Hesse D, Hopman WM, Borschneck D, Bardana D; 2017	RCT; Arthroscopic skill acquisition was compared using high-fidelity virtual reality simulators and low-fidelity bench-top setups. After training, participants performed diagnostic arthroscopy on simulators and a cadaveric knee in a simulated intraoperative environment, while a control group received no training.	Participants were evaluated using the Global Rating Scale, the 14-point arthroscopic checklist, time required.	Both simulators resulted in an improvement over the measurements taken. The VR group showed a slightly better, but not statistically significantly different, performance.
Arthroscopy	Gandhi MJ, Anderton MJ, Funk L; 2015	Quantitative, descriptive; An online arthroscopic skills acquisition tool was used to assess participants’ learning of basic arthroscopic skills. They were trained using this tool and later evaluated using the same tool and a virtual reality shoulder arthroscopy simulator.	Overall performance based on factors such as time taken, distance traveled, locate-and-palpate.	Significant correlations between the performance on the online arthroscopic skills acquisition tool and the validated virtual reality simulator were found.
Arthroscopy	Gomoll AH, O’Toole RV, Czarnecki J, Warner JJP; 2007	Quantitative, descriptive; The effectiveness of a virtual reality arthroscopy simulator was assessed to determine whether performance on the simulator correlates with actual surgical experience. The study focused on skills related to shoulder arthroscopy.	Time to completion, distance traveled with the tip of the simulated probe compared with a computer-determined optimal distance, average probe velocity and number of probe collisions with the tissues.	Average time to completion decreased after training. Path length and hook collisions were more than halved. Average probe velocity doubled.
Arthroscopy	Henn RF, Shah N, Warner JJP, Gomoll AH; 2013	RCT; The research compares the benefits of shoulder arthroscopy simulator training by having students practice on a virtual reality simulator after completing a baseline arthroscopy on a cadaveric shoulder. It includes an intervention group using the simulator and a control group receiving no further training.	Time to task completion, assessment of technical performance and change over time within groups.	The intervention group improved significantly from baseline in time to completion and performance. Time to completion was significantly faster in the simulator group than in the control group. No difference was observed between the groups in scores at the final evaluation.
Arthroscopy	Ström P, Kjellin A, Hedman L, Wredmark T, Felländer-Tsai L; 2004	RCT; Participants in the intervention group trained for one hour on three different simulators before performing the test on the virtual arthroscopy knee simulator. The control group did not receive prior training on additional simulators and proceeded directly to testing on the virtual arthroscopy knee simulator.	Time to complete tasks, movement economy, collision rates with the scope and probe and total score in the virtual arthroscopy knee simulator.	No significant differences in the performance outcomes between the intervention and control group could be found.
Basic practical nursing skills	de Araujo DF, da Costa RRO, Nogueira LT, do Nascimento JSG, Rodrigues IDCV, de Sousa LA, de Carvalho EC; 2021	RCT; The study evaluated the impact of a virtual simulation strategy on nursing students’ clinical judgment in managing critically ill COVID-19 patients. The intervention group engaged in interactive simulation scenarios with decision-making and debriefing, while the control group received traditional online lectures covering the same material. The virtual simulation platform provided real-time patient management cases with feedback, targeting skills in assessment, prioritization, and intervention.	Primary outcome was clinical judgment performance measured by a validated assessment rubric; secondary outcome was student satisfaction.	The intervention group demonstrated significantly higher clinical judgment scores than the control group, along with higher satisfaction ratings.
Basic practical nursing skills	Geist R, Kiper V, Chapman R, Harris S, Caballero S, Opton L, Decker S; 2022	Quantitative, descriptive; The modular skills training box, which included an injection pad, wound, female genitalia and a face with openings in the nares, mouth and neck, was used for practical nursing skills training. Participants used the box for one week and provided feedback on skills such as wound care, ostomy care and nasogastric intubation.	Students were to rate use, the efficacy of skills performance, comfort with the learning format and understanding of each skill performed.	Student acceptance of the modular skills training box was high and its contribution to the effectiveness of student skills performance was rated as high. Students indicated a higher comfort in skill performance after training with the modular skills training box.
Basic practical nursing skills	Haerling KA; 2018	RCT; The study compared virtual simulation with mannequin-based simulation for training in the management of COPD exacerbations. Participants in the intervention group completed a screen-based virtual simulation, while controls engaged in the same case using a mannequin. All participants underwent pre- and post-simulation assessments, with a subsample evaluated via standardized patient performance. Skills emphasized included clinical reasoning, patient care management, and communication. The study also conducted a cost-utility analysis comparing the two simulation modalities.	Outcomes included quantitative measures of learning and performance, qualitative reflections, and a cost‑utility ratio for each simulation modality.	Results showed no significant differences in quantitative learning or performance outcomes between the two groups; qualitative feedback provided additional insight; virtual simulation had a more favorable cost‑utility ratio of US $1.08 compared to US $3.62 for mannequin‑based simulation.
Basic practical nursing skills	Koukourikos K, Tsaloglidou A, Kourkouta L, Papathanasiou IV, Fratzana A, Panagiotou A; 2021	RCT; The study assessed the effectiveness of virtual simulation in improving decision-making skills among nursing students. The intervention group participated in interactive virtual simulation scenarios requiring patient assessment, prioritization, and response, while the control group received traditional lectures and case studies. Both groups completed pre- and post-tests, but only the intervention group received simulation training. The program targeted skills in clinical assessment, critical thinking, prioritization of care, and decision-making.	The outcomes measured were clinical decision-making ability and satisfaction with the learning method.	The results showed a statistically significant improvement in clinical decision-making skills in the intervention group compared to the control group. The students in the experimental group also expressed higher satisfaction with the training approach.
Basic practical nursing skills	Li L, Song Y, Zhang H, Gao J, Wang J, Sun Y; 2023	RCT; The study evaluated the effectiveness of a virtual simulation program for undergraduate nursing students in recognizing and managing patient deterioration. The intervention group completed interactive online simulation scenarios, while the control group received traditional lectures and case-based teaching. Both groups had the same theoretical instruction and pre- and post-tests, but only the intervention group experienced virtual simulation. The training targeted skills in early recognition of deterioration, clinical decision-making, and appropriate nursing interventions.	The outcomes measured were theoretical knowledge, clinical decision-making ability, and satisfaction with the learning method.	The results showed that the experimental group had significantly higher post-test scores in knowledge and decision-making ability compared to the control group. They also reported higher satisfaction with the learning experience.
Basic practical nursing skills	Reilly A, Spratt C; 2007	Qualitative, descriptive; Nursing students and academic teachers participated in high-fidelity simulation sessions using mannequins designed to create realistic patient scenarios, focusing on patient care, communication and critical decision-making. The study explored their perceptions of how this simulation contributed to clinical practice preparation.	Students’ perceptions of learning through high-fidelity simulation, explored through focus group interviews.	Students reported that the high-fidelity simulation improved their confidence and prepared them well for clinical practice. They appreciated the active, hands-on learning and believed it enhanced their understanding of patient care. Academic staff praised the pedagogical benefits of simulation-based learning.
Basic surgical skills	Cornejo-Carrasco CE, Gonzales-Menéndez MJM, Hinostroza-Castillo R, Flores-Yábar JR, Carrasco-Rivera CE; 2023	Quasi-experimental; This study evaluated undergraduate medical students’ ability to perform open and laparoscopic appendectomy and purse-string sutures using a home-built, low-cost simulation model during virtual teaching sessions. Students constructed their own models from inexpensive materials, attended synchronous online sessions to learn surgical techniques, and practiced under remote instructor guidance and feedback. Outcomes included competency in appendectomy and suturing, student satisfaction, and cost assessment of the model.	Surgical performance scores for each procedure and student satisfaction levels; costs of model construction.	All students achieved the expected competencies, with high satisfaction reported and very low construction costs for the model.
Basic surgical skills	Farquharson AL, Cresswell AC, Beard JD, Chan P; 2013	RCT; This study assessed the use of video recordings as a tool to improve feedback in teaching basic surgical skills, specifically skin suturing. Participants who received video-assisted feedback performed the skill again the next day, while those in the control group received only standard verbal feedback.	A standard, validated assessment checklist was used to evaluate skill performance comprising choice of appropriate suture, knot quality and economy of movement.	Overall performance of the intervention group increased significantly between trials, while no improvement was seen in the control group. Significant improvements for the intervention group were found in specific domains (instrument familiarity, needle handling, skin handling and accurate apposition). Organization of task approach improved for both groups. Safety of knot tying worsened in the control group but not in the intervention group.
Basic surgical skills	Kazemi H, Rappel JK, Poston T, Lim BH, Burdet E, Teo CL; 2010	Quasi-experimental; The study evaluated a virtual reality microsurgical suturing simulator for its ability to differentiate expert from novice performance, measure technical skills, and test the effect of needle shapes on outcomes. Participants completed 30 standardized suturing tasks with randomized virtual needle shapes. Both intervention and control groups (medical staff vs. nonmedical students) used the same simulator under identical conditions. Skills assessed included needle handling, entry/exit angles, motion smoothness, and tissue handling.	Differences in motion smoothness, tissue trauma, penetration/exit angles, and orientation change were examined to distinguish trained from untrained subjects and evaluate needle shape effects.	Trained participants had significantly better penetration and exit angles, caused less tissue damage, and showed better orientation control than untrained participants; no significant difference in completion time or grasp number. Needle shape influenced tear size and time, with the J-shaped needle generally performing best.
Basic surgical skills	Sakamoto Y, Okamoto S, Shimizu K, Araki Y, Hirakawa A, Wakabayashi T; 2017	RCT; The study compared bench model hands-on learning with traditional video-based learning for teaching basic microsuturing skills to medical students. The intervention group practiced suturing on a bench model with woolen yarn before microsuturing gauze fibers under a microscope, while the control group watched an instructional video before the same microscope practice. Both groups performed microsuturing with 10 − 0 nylon and tied three knots in a timed test. Skills emphasized included microsuturing precision and knot-tying techniques.	Primary: Time to complete microsuturing task and quality of final knots (NUMSAS score); Secondary: Relationship between personality traits and performance.	Simulator group completed tasks significantly faster (mean 749.7 s) than video group (mean 1115.2 s, *p* = 0.001); quality scores slightly higher in simulator group but not statistically significant except for better suture length score; higher extraversion linked to faster completion; higher agreeableness linked to slightly lower quality scores; women tended to score higher than men, but difference not significant.
Basic suturing skills	McClinton TD; 2023	Quasi-experimental; Participants practiced basic suturing skills using a virtual suturing lab, which included pre-recorded instructional videos, live virtual sessions and practice kits. They received instructions, participated in small breakout groups and completed a skills check-off.	Students’ ability to demonstrate competency in basic suturing techniques.	Students expressed their confidence in their ability to perform suturing techniques and their readiness for clinical rotations.
Blood sample examination	Moreno-Ger P, Torrente J, Bustamante J, Fernández-Galaz C, Fernández-Manjón B, Comas-Rengifo MD; 2010	RCT; The study evaluated the impact of a low-cost, web-based simulation on improving medical students’ practical skills in blood sample examination, specifically measuring hematocrit. Participants practiced the procedure using the simulation before lab sessions, while the control group followed traditional learning methods without prior simulation.	Students’ perceived difficulty in performing the blood examination procedure, the variability of their measurements and their satisfaction with the simulation.	The intervention group reported significantly lower perceived difficulty in performing the procedure and had more consistent and precise measurements compared to the control group.
Bronchoscopy	Gopal M, Skobodzinski AA, Sterbling HM, Rao SR, LaChapelle C, Suzuki K, Litle VR; 2018	Quantitative, descriptive; Students participated in a series of self-directed training sessions using a high-fidelity, haptic feedback virtual reality bronchoscopy simulator to acquire anatomical knowledge and practical bronchoscopy skills. Post-training performance was measured after completing four 15-minute weekly sessions.	Bronchial anatomy knowledge and bronchoscope navigational skills as well as a total performance score.	Mean scores for bronchial anatomy and bronchoscopy navigational skills increase significantly. Mean total performance scores increased significantly from pre- to post-training sessions.
Cardiopulmonary resuscitation	Bonnetain E, Boucheix JM, Hamet M, Freysz M; 2010	Quasi-experimental; The effectiveness of a multimedia computer screen-based simulator for teaching cardiopulmonary resuscitation (CPR) skills in a cardiac arrest scenario was assessed. Participants in the simulator group were compared with a traditional class group, with both groups tested using a high-fidelity patient simulator in a different cardiac arrest scenario.	Cardiopulmonary resuscitation performance on the high-fidelity simulator was evaluated using a 23-item examination questionnaire.	The intervention group performed significantly better than the control group on the high-fidelity simulator.
Cardiopulmonary resuscitation	Jaskiewicz F, Kowalewski D, Starosta K, Cierniak M, Timler D; 2020	Quasi-experimental; The study evaluated whether using virtual reality in a sudden cardiac arrest scenario impacted the quality of chest compressions. Additionally, it assessed whether the use of extra equipment made performing chest compressions subjectively harder.	Quality of chest compressions as measured by heart rate, depth and full chest relaxation. In addition, the subjective difficulty of performing chest compressions with the virtual reality device was examined and students’ impressions of the effectiveness of the virtual reality training were assessed.	There was no significant difference in chest compression rate or depth between the traditional scenario and virtual reality scenario groups. Complete chest relaxation was significantly lower in the virtual reality group. Students generally found the virtual reality training to be effective. The majority of students found that virtual reality training made chest compressions more difficult to perform.
Catheter ablation	Wang H, Wu J; 2021	Quasi-experimental; Trainees conducted a simulated catheter ablation procedure within a virtual reality-based system, navigating catheters and interacting with a simulated beating heart. The simulator provided real-time haptic feedback to enhance procedural accuracy and technique.	Participant feedback on simulator realism, real-time responsiveness and perceived usefulness for skill training and assessment.	The simulator was positively rated in terms of realism. Real-time usage was rated positively, although haptic feedback realism received lower ratings due to limited force feedback capabilities. Overall, participants found the simulator to be a useful tool for training catheter ablation skills.
Cricothyrotomy	Nour MG, Moradi EY, Aljamal YN, Arghami A, Sadrzadeh SM, Assadi R; 2023	RCT; This research evaluated the educational value and student satisfaction with a low-cost, hand-made cricothyrotomy simulation model compared to a high-fidelity model. Participants performed emergency cricothyrotomy procedures using both models within simulated failed intubation scenarios.	Overall performance during the cricothyrotomy as well as students’ satisfaction with the learning format.	No significant differences between the two groups in terms of their performance was found. Students evaluated their experience with the low-cost model positively.
Endoscopy	Pereferrer FS, González MH, García AM, Vilanova AC, Déjardin DDC; 2013	RCT; The study evaluates the learning curve of surgical skills through a virtual endoscopic simulator. Participants completed three training sessions focused on basic endoscopic skills, while the control group did not undergo training but participated in baseline and post-training tests.	Students’ performance scores in each of the seven exercises.	All groups showed significant improvements after the training. Sixth-year students performed significantly better than second- and fourth-year students in tasks such as cutting and lifting.
Endovascular surgery	Aeckersberg G, Gkremoutis A, Schmitz-Rixen T, Kaiser E; 2019	RCT; Students attended a 1-day course on basic endovascular skills, followed by different training methods. Intervention groups used low-fidelity simulation training with tablet-based navigation (gesture-based or physical tool), while the comparison group learned through a video podcast.	Self-assessment items such as: -I think this course is relevant to my future, - I think endovascular procedures are interesting, - I can see myself working in vascular surgery in the future.	For most factors, no significant differences were found between the conventional learning group and the two simulation training groups. However, motivation was higher after simulator training.
Endovascular surgery	Van Herzeele I, D’Donoghue KGL, Aggarwal R, Vermassen F, Cheshire NJW; 2010	Quasi-experimental; Medical students underwent cognitive and endovascular skills training using a virtual reality simulator, repeatedly practicing a renal artery stenting procedure over ten sessions to measure skill development. Their visuospatial and psychomotor abilities were evaluated concerning endovascular navigation, tool handling and procedural efficiency.	Quantitative simulator metrics and external assessments by an experienced interventionalist using rating scales for generic and procedure-specific skills.	Students showed significant improvement in simulator metrics over ten sessions. Higher scores on certain aptitude tests correlated with better initial and final simulator performance.
Hysteroscopy	Gat I, Kedem A, Seidman DS, Yogev Y, Lavie O; 2022	RCT; The study evaluated whether a VR hysteroscopy simulator (VirtaMed HystSim) could effectively teach basic hysteroscopic skills to medical students. The intervention group received standard theoretical instruction plus simulator training with multiple practice procedures, while the control group received theory only. All students were assessed on the simulator before and after training to measure improvements. Skills targeted included safe hysteroscope insertion, uterine navigation, visualization of landmarks, and recognition of intrauterine findings.	Simulator-derived performance scores, procedure completion times, number of wall collisions, and proportion of uterine cavity visualized.	The intervention group showed significantly greater improvement in overall simulator performance scores, shorter procedure times, fewer wall collisions, and a higher percentage of uterine cavity visualized compared to the control group.
Intravenous cannulation	Reyes SD, Stillsmoking K, Chadwick-Hopkins D; 2008	RCT; Students practiced intravenous catheter insertion using a virtual simulator with haptic feedback after a one-hour orientation, while the control group trained with mannequins in a nursing lab. Both groups completed pre- and post-tests, including cognitive assessments and live initiation evaluations.	Cognitive gains from pre- to post-tests and skill acquisition through success rate of live attempts. Also, students’ satisfaction with the virtual simulator was measured.	Both groups showed cognitive improvement with no significant difference between them. However, the control group had a higher success rate in live intravenous cannulation compared to the simulation group.
Intravenous catheterization	Isaranuwatchai W, Brydges R, Carnaham H, Backstein D, Dubrowski A; 2013	Quasi-experimental; Participants in three intervention groups used different simulation modalities: a virtual reality trainer, a mannequin-based simulator or both. One week after simulation training, all subjects performed a transfer test on a hybrid simulation with a standardized patient.	Learning effectiveness as rated by two blinded expert raters using the Direct Observation of Procedural Skills (DOPS) tool. For the cost-effectiveness analysis, the DOPS score was used as the clinically relevant outcome measure.	The learning format of intervention group 3 had the highest total cost, while the format for intervention group 2 had the highest implementation cost. The program for intervention group 3 was generally the most educationally- and cost-effective.
Laparoscopy	Brinkmann C, Fritz M, Pankratius U, Bahde R, Neumann P, Schlueter S, Senninger N, Rijcken E; 2017	RCT; The study compared the effectiveness of a VR-driven trainer and a box trainer for teaching basic laparoscopic skills, including camera navigation, pattern cutting and knot binding. Participants practiced their skills using both training methods and then applied what they learned in an ex situ laparoscopic cholecystectomy on a pig liver.	Performance was evaluated by 4 blinded observers using the Global Operative Assessment of Laparoscopic Skills score. Also, learning curves were tracked and compared and the improvement in each exercise was analyzed.	Participants in both training conditions showed significant improvements compared to baseline scores. There was no difference between groups regarding learning curves. In respect to the simulated laparoscopic cholecystectomy, total assessment scores were significantly higher for the box-trained group than the VR-trained group.
Laparoscopy	Debees AJ, Aggarwal R, Balasundaram I, Jacobsen MB; 2010	RCT; This study evaluated the effectiveness of a virtual reality-based training format for acquiring basic laparoscopic skills, where participants were trained using the MIST-VR simulator and underwent pre- and post-assessments on both the simulator and box trainer. In comparison, participants in the control group practiced with a box trainer and were later assessed on the virtual reality simulator.	Outcomes included time to complete the task, number of hand movements and total path length. Additionally, for the virtual reality condition, economy of movement and a total score were measured.	The virtual reality group showed significantly shorter time, better economy of movements and a better total score. Regarding the assessment on the box trainer, no difference was found for time to complete the task, hand movements and total path length.
Laparoscopy	Diesen DL, Erhunmwunsee L, Bennett KM, Ben-David Kfir, Yurcisin B, Ceppa EP, Omotosho PA, Perez A, Pryor A; 2011	RCT; The study involves evaluating the effectiveness of laparoscopic training using a computer simulator compared to a box trainer, both of which focus on skills like camera movement, instrument handling and object positioning. The training is supervised and participants complete a series of laparoscopic exercises on a live porcine model at different time intervals.	Participants were tested on camera handling, needle transfer, scope tracking, clipping and cautery and knot tying.	An overall improvement in scores was found for both simulators. No difference was found between the box simulator participants versus the participants with the computer-based simulator.
Laparoscopy	Fu Y, Cavuoto L, Qi D, Panneerselvam K, Arikatla VS, Enquobahrie A, De S, Schwaitzberg SD; 2020	RCT; The VBLaST-SS virtual basic laparoscopic skill trainer was used to assess students’ acquisition of basic laparoscopic skills. The training focused on intracorporeal suturing, including knot tying, cutting and needle piercing.	Procedure deviations, incision gap and knot security were used to calculate an overall performance score. Also, completion time was measured.	In-group performance improved significantly after training. Failure rates were found to be reduced by the end of the training period.
Laparoscopy	Hagelsteen K, Langegard A, Lantz A, Ekelund M, Anderberg M, Bergenfelz A; 2017	Quasi-experimental; This study examines whether 3D vision and haptic feedback in a virtual reality environment enhance the efficiency of laparoscopic skills learning. Participants trained with a virtual reality trainer featuring these advanced technologies, while a control group trained without them and all participants completed a course in the virtual reality trainer.	Time taken for training course completion, number of attempts to reach proficiency and scores for each individual tasks.	The intervention group took significantly less time to complete the course and had significantly fewer attempts to reach proficiency. The study group showed significantly faster skill acquisition in three out of four of the individual tasks.
Laparoscopy	Hiemstra E, Terveer EM, Chmarra MK, Dankelman J, Jansen FW; 2011	RCT; Three intervention groups were tested with different setups: a conventional virtual reality laparoscopic trainer, one with additional kinematic interaction and a box trainer setup. Participants performed tasks involving cylinder handling and tissue handling on a virtual reality laparoscopy simulator.	Time taken to perform the task, total path length of the instrument while performing the task and motion in depth, i.e. the total distance travelled by right and left instrument along its axis.	While the control group did not improve significantly, the intervention group 1 improved regarding time taken, whereas the intervention group 2 and the box trainer groups improved in time, path length and motion in depth.
Laparoscopy	Kamat A, Makled B, Norfleet J, Intes X, Dutta A, De Suvranu; 2021	Quasi-experimental; This study investigates differences in brain connectivity between students using physical and virtual laparoscopic simulators. Both groups performed the Fundamental Laparoscopic Surgery pattern cutting task while their brain activity was measured using optode montages.	Effective brain connectivity between various brain regions and the task performance score of the students during the pattern cutting task.	A statistically significant difference in brain connectivity between the left and right primary motor cortices was found during the initial stages of the cutting task on the physical simulator compared to the virtual simulator. Alternating activity was found as the task progressed in both groups.
Laparoscopy	Laski D, Stefaniak TJ, Makarewicz W, Proczko M, Gruca Z, Sledzinski Z; 2012	Quasi-experimental; Participants trained on box trainers and completed three virtual tasks: “peg transfer,” “virtual cutting,” and “virtual clipping.” They focused on laparoscopic surgery-related skills like transferring objects, cutting and clipping with laparoscopic tools.	Task execution time and motion path of the right and left hands.	A statistically significant reduction in task completion time for all exercises and a significant shortening of the left-hand motion path was found after training.
Laparoscopy	Loukas C, Nikiteas N, Schizas D, Lahanas V, Georglou E; 2012	RCT; This study compared the effectiveness of virtual reality simulators and physical reality video trainers in teaching basic laparoscopic skills. Participants performed tasks like peg transfer and knot-tying using both types of trainers, switching after completing each task.	Performance metrics such as path length, time taken to complete tasks and a penalty score reflecting errors during the procedure.	Both training modalities significantly improved performance on the respective tasks. The skills learned on the virtual reality simulator were transferable to the video trainer and vice versa. For knot-tying, virtual reality training did not result in performance equivalent to the physical trainer.
Laparoscopy	Matzke J, Ziegler C, Martin K, Crawford S, Sutton E; 2017	Quasi-experimental; Participants completed a virtual reality-based training curriculum and were assessed on laparoscopic tasks using both a virtual reality simulator and a physical FLS trainer box. Their performance in both environments was compared, focusing on metrics like time limits, accuracy and errors.	Penalties for errors during the tasks and time taken to complete tasks.	The intervention group showed less penalties on circle cutting, ligating loop and peg transfer tasks. Proficiency was not universally achieved, particularly for knot-tying.
Laparoscopy	Mulla M, Sharma D, Moghul M, Kailani O, Dockery J, Ayis S, Grange P; 2012	RCT; This research compared different methods for learning basic laparoscopic skills, including a box trainer, a virtual reality simulator and mental training. Participants performed laparoscopic tasks like cutting and suturing, with groups receiving different types of training before being tested on both the box trainer and virtual reality simulator.	Time to completion, precision, accuracy and overall performance of the cutting process.	The box-trained group performed best on the box trainer, while the virtual reality-trained group performed best on the virtual reality simulator. Mental training alone was the least effective method. Skills learned on the box trainer were transferable to the virtual reality simulator but not all skills learned on the virtual reality simulator were transferable to the box trainer.
Laparoscopy	Lucas S, Tuncel A, Bensalah K, Zeltser I, Jenkins A, Pearle M, Cadeddu J; 2008	RCT; Medical students trained on the LAP Mentor VR simulator in six unsupervised 30-minute sessions over three weeks, while controls received no training; both groups performed identical baseline and post-training simulated cholecystectomies.	Performance was evaluated using OSATS total and category-specific scores assessing dexterity, tissue handling, procedural flow and overall operative ability.	VR-trained students showed markedly greater improvement and significantly higher final OSATS scores than controls, demonstrating the effectiveness of VR simulation for enhancing laparoscopic skills in novices.
Laparoscopy	Vitish-Sharma P, Knowles J, Patel B; 2011	Quasi-experimental; Medical trainees practiced laparoscopic skills using either a virtual reality simulator or a traditional box trainer. The virtual reality group completed tasks focused on hand-eye coordination, bimanual manipulation and instrument control, while the box trainer group performed physical exercises like ring transfers and suturing.	Total time taken, number of movements and path length for both right and left instruments during the laparoscopic task.	Both the virtual reality and box trainer groups showed improvement across all measured parameters after training. However, there was no statistically significant difference between the two groups in terms of skill acquisition.
Laparoscopic bead transfer and suturing	Ju R, Chang PL, Buckley AP, Wang KC; 2012	Quasi-experimental; This study compared the effectiveness of the Nintendo Wii and PlayStation 2 in enhancing laparoscopic skills. Participants either played the Nintendo Wii (game: Boomblox) or PlayStation 2 (game: Time Crisis 2) for 30 min and then performed bead transfer and suturing tasks using a box trainer before and after the training.	Overall performance scores for bead transfer and suturing tasks.	Both Wii and PlayStation 2 showed significant improvement in bead transfer scores. No improvements were found for suturing scores. Participants who used the Wii console improved more in bead transfer scores than participants who used the PlayStation 2, though the difference was not statistically significant.
Laparoscopy and cholecystectomy	da Cruz JAS, dos Reis ST, Frati RMC, Duarte RJ, Nguyen H, Srougi M, Passerotti CC; 2016	RCT; This study examined the added value of a preoperative warm-up session using a virtual reality surgical simulator (VRSS) before performing laparoscopic cholecystectomy in a porcine model. Participants in the control group directly performed the procedure without prior simulation training.	Quantitative: time for dissection of the gallblader pedicle, time for clipping the pedicle, time for cutting the pedicle, time for gallbladder removal, total operative time and aspirated blood loss. Qualitative: depth perception, bimanual dexterity, efficiency, tissue handling and autonomy.	The intervention group showed significantly superior results comprising time for dissection, time for clipping the pedicle, time for cutting the pedicle, aspirated blood loss, depth perception, bimanual dexterity, tissue handling and autonomy.
Laparoscopy and cholecystectomy	Kowalewski KF, Minassian A, Hendrie JD, Benner L, Preukschas AA, Kenngott HG, Fischer L, Müller-Stich BP, Nickel F; 2019	RCT; Individuals underwent a training curriculum that included e-learning modules on laparoscopic cholecystectomy (LC), box trainers and virtual reality trainers to practice basic and procedural laparoscopic skills. The training program involved virtual reality sessions using the LAP Mentor II, where participants performed LC procedures and received additional video content for learning.	Overall performance for general laparoscopic skills and specific procedural skills as well as overall score for time taken to complete the LC and performance on the virtual reality trainers.	No significant differences between the alone and dyad groups were found on performance scores. Dyad training reduced the time to completion regarding the LC procedure. Both intervention groups outperformed the control group on virtual reality training metrics.
Laparoscopy and endoscopy	Willis RE, Gomez PP, Ivatury SJ, Mitra HS, Van Sickle KR; 2014	Quasi-experimental; Trainees completed tasks on both a virtual reality simulator and a physical model simulator, followed by a video game designed to evaluate fine motor skills. Their performance across all three activities was analyzed to determine potential correlations between gaming proficiency and surgical skill acquisition.	Correlations between video game performance, virtual reality simulator performance and physical model simulator performance, specifically regarding time to completion and movement trajectory in laparoscopic and endoscopic tasks.	Virtual game performance correlated strongly with virtual reality simulator performance but not with physical model simulator performance.
Laparoscopy and robot-assisted laparoscopy	Moglia A, Sinceri S, Ferrari V, Ferrari M, Mosca F, Morelli L; 2018	Quasi-experimental; The study evaluated proficiency-based training for medical students using virtual reality simulators for both manual laparoscopic surgery and robot-assisted laparoscopic surgery. Participants attended lectures on surgical simulation and practiced skills like instrument handling, camera navigation and suturing on both types of simulators.	Number of attempts required to reach proficiency along with metrics like task completion time and errors made.	Students with previous experience on surgical simulators required fewer attempts to reach proficiency, although the differences were not statistically significant. Tasks like cutting and catheter insertion were challenging for students with no prior experience.
Mastoidectomy	Wijewickrema S, Zhou Y, Ionnou I, Copson B, Piromchai P, Yu C, Briggs R, Bailey J, Kennedy G, O’Leary S; 2018	RCT; Trainees completed a virtual mastoidectomy procedure using a virtual reality-based simulator, with one group receiving full procedural guidance and another following a step-by-step guided approach. A control group performed the procedure without automated guidance to assess the impact of different instructional modalities on skill acquisition.	Quality of dissection, assessed using the Welling scale and the percentage of time participants used guidance during the optional phase of the simulation.	It was found that both types of automated guidance significantly improved participants’ dissection quality. The step-by-step guidance method was used more frequently and for longer periods, indicating higher engagement compared to full guidance​.
Medication administration	Dubovi I, Levy ST, Dagan E; 2017	Quasi-experimental; Participants in the experimental group used a virtual reality simulation to learn medication administration procedures, while the control group used a lecture-based format. Both groups trained for a total of eight sessions.	A knowledge test was applied comprising conceptual and procedural questions regarding medication administration.	Significantly higher conceptual and procedural knowledge scores were found for the virtual reality-based group compared to the conventional lecture-based format.
Neonatal resuscitation	Barré J, Michelet D, Truchot J, Cabon P, Tesniere A; 2020	Quasi-experimental; A screen-based simulation training was examined for its effectiveness in teaching neonatal resuscitation skills. The study evaluated how this digital training method supports skill acquisition in a critical care setting.	A knowledge quiz on neonatal resuscitation, self-efficacy assessment, two experts’ evaluations of the Anesthetists’ Non-Technical Skills (ANTS) and Neonatal Resuscitation Performance Evaluation (NRPE).	Participants showed improvements regarding self-efficacy, knowledge and anesthetic non-technical skills (cognitive, social and personal resource skills). No differences were found in respect to neonatal resuscitation performance per se.
Otolaryngologic surgery	Hardcastle T, Wood A; 2018	Quantitative, descriptive; This study evaluates the effectiveness of a virtual reality surgical simulator for teaching surgical skills on the temporal bone in an otolaryngology curriculum. Students were pre-tested on their career interests before using the simulator.	A post-workshop questionnaire was used to assess the students’ perceived usefulness and enjoyment of the virtual reality surgical simulator. Changes in students’ career perspectives were also assessed.	Students rated the virtual reality surgical simulator useful for simulating thoughts around career plans, providing hands-on experience and teaching disease processes.
Otoscopy	Davies J, Djelic L, Campisi P, Forte V, Chiodo A; 2014	Quantitative, descriptive; The study assessed the effectiveness of an otoscopy simulator for teaching the primary principles of otoscopy. Participants underwent a teaching session using the otoscopy simulator to develop otoscopy-related skills.	A nine-question survey comprising, among other factors: the overall quality of the event, degree to which the event improved confidence in using an otoscope and degree to which the event stimulated interest in otolaryngology.	Quality of the training session was rated very good to excellent. 71% of respondents found the otoscope simulator training format to be effective. 70% found that the training session had stimulated their interest in the subject.
Otoscopy	Stepniak C, Wickens B, Husein M, Paradis J, Ladak HM, Fung K, Agrawal SK; 2016	RCT; Medical students used a web-based otoscopy simulator for one week to practice diagnostic otoscopy skills, in addition to standard otology lectures. The control group attended the same lectures but did not have access to the simulator.	Post-test scores assessing diagnostic accuracy, with metrics such as tympanic membrane visualization and final diagnosis, compared between pretest and post-test.	The intervention group improved their scores by 71% from pretest to post-test, whereas the control group improved by 31%. The simulator group scored 24% higher than the control group on the post-test, with significant improvements in specific areas like tympanic membrane visualization.
Otoscopy	Wu V, Beyea JA; 2016	RCT; Participants were divided into two intervention groups: one used an otoscopy simulation system with visual guidance, while the other completed a web-based module containing instructional slides and ear pathology images. The control group received traditional classroom instruction through a slide-based lecture by an otolaryngologist.	Diagnostic accuracy in identifying ear pathologies. The secondary outcome was otoscopy clinical skills, measured with a checklist of procedural steps in otoscopy.	All groups showed improvement regarding post-test accuracy, with the simulation and web-based format showing significantly higher scores and the standard learning format. For otoscopy clinical skills, only the simulation condition demonstrated significant improvement, which was retained at the 3-month follow-up.
Otoscopy	Xu J, Campisi P, Forte V, Carrillo B, Vescan A, Brydges R; 2018	RCT; Trainees were divided into two groups: one used the mobile otoscopy simulator independently before attending a lecture, while the other attended the lecture first and then used the simulator for a week. Both groups completed a pre-test, post-test and retention test to measure knowledge acquisition and retention.	Knowledge test scores at baseline, post-intervention and two-week retention points served as primary outcomes. Secondary outcomes were self-reported comfort with otoscopy, time spent using the simulator and learning preferences.	Both groups improved in knowledge acquisition and retention, with no significant difference between the groups. The first group showed significant improvement in self-reported comfort, whereas the instruction then discovery groups did not.
Physical examination	Kern DH, Mainous III AG, Carey M, Beddingfield; 2011	Quasi-experimental; The evaluation examines whether adding simulation to standardized patient training improves physical examination skills. Participants who received training with both a standardized patient and a cardiac simulator were compared to those who trained with a standardized patient alone.	Overall scores for the cardiac physical examination performance assessed by a standardized assessment checklist for cardiopulmonary examination.	Students who trained with the simulator performed significantly better than the control group.
Physical patient examination and treatment	Gordon JA, Wilkerson WM, Shaffer DW, Armstrong EG; 2001	Mixed-methods, descriptive; This research explored the subjective impressions of medical students and educators using a high-fidelity patient simulation tool to evaluate patient examination and treatment skills. Participants completed the simulation and provided feedback on the realism and educational value of the experience.	Subjective student and instructor ratings of their simulator impressions in terms of overall experience, realism and educational value.	Participants rated the simulator session as excellent or very good. Both groups felt that simulator-based training should be required for all medical students. The simulation was considered “very realistic” and a “broad educational tool” for acquiring patient assessment and treatment skills.
Pulmonary artery catheterization	Wise EM, McIvor WR, Mangione MP; 2016	Quasi-experimental; Trainees engaged with a web-based simulation designed to teach pulmonary artery catheter insertion, completing interactive modules at their own pace during the clerkship. The simulation provided real-time feedback on procedural steps and assessed their understanding of cardiac physiology through embedded quizzes.	Usage patterns of the web-based simulation (such as time of use and duration) as well as self-reported understanding of pulmonary artery catheter usage before and after using the simulation.	Most students found the web-based simulation beneficial, rating it higher than textbook learning and comparable to lectures and interactive discussions. Students’ self-rated understanding of pulmonary artery catheter usage improved significantly after using the simulation.
Sonography	Damewood S, Jeanmonod D, Cadigan B; 2011	Quasi-experimental; The study compared the effectiveness of a multimedia ultrasound simulator with conventional human models for teaching sonographic image acquisition. Participants performed the FAST exam, interpreting prerecorded ultrasound images in both training formats.	Image interpretation of prerecorded FAST exams, adequacy of image acquisition on a standardized normal patient, perceived confidence of image adequacy and time to image acquisition.	No differences could be found between groups regarding image interpretation, image acquisition, trainee’s confidence and time to acquire images.
Sonography	Meuwly JY, Mandralis K, Tenisch E, Gullo G, Frossard P, Morend L; 2021	Quasi-experimental; The study evaluated an online ultrasound simulator for teaching medical students basic psychomotor skills, such as probe navigation and reproducing diagnostic ultrasound images. Participants practiced ultrasound exercises using a virtual mannequin with real patient image data.	Psychomotor skill acquisition and students’ satisfaction with the learning format.	A significant improvement was found in student performance after using the simulator. A general satisfaction with the simulator was reported.
Sonography	Weimer JM, Sprengart FM, Vieth T, Göbel S, Dionysopoulou A, Krüger R, Beer J, Weimer AM, Buggenhagen H, Kloeckner R, Pillong L, Helfrich J, Waezsada E, Wand P, Weinmann-Menke J; 2025	RCT; The study compared simulator-based training with human-based training for teaching Focused Assessed Transthoracic Echocardiography (FATE) to undergraduate medical students. The simulator group trained exclusively with the Vimedix high-fidelity ultrasound simulator, while the control group practiced only on human subjects, both within a structured 420-minute workshop. Assessments included pre- and post-tests, self-assessment questionnaires, and practical DOPS exams on both simulator and human patients. Skills taught included performing FATE cross-sections, ultrasound device handling, examination procedures, patient guidance, and recognition of normal and pathological findings.	Both groups showed significant gains in theoretical knowledge and self-assessed competence; simulator group performed significantly worse than control group in human-based practical test; both groups supported simulator use as a supplement, not a replacement for human training.	Theory scores improved significantly in both groups, with simulator group showing greater theoretical gain; practical scores on simulator were similar between groups, but control group scored higher in human practical test; simulator group rated realism and overall learning experience lower than control group.
Sonography and lumbar puncture	Katz LM, Finch A, McKinnish T, Gilliland K, Tolleson-Rinehart S, Marks BL; 2017	Quasi-experimental; This study evaluated the impact of a multifaceted procedural skills lab on medical students’ confidence in performing various procedural skills, including FAST-sonography and lumbar puncture. The intervention group used the lab with instructional videos, spaced education and practice, while the control group did not and both groups completed pre- and post-surveys to assess confidence and experience.	Change in students’ confidence levels in performing procedural skills.	The intervention group showed a significant increase in confidence for all procedural skills after the intervention.
Surgery preparation	del Blanco Á, Torrente J, Fernández-Manjón B, Ruiz P, Giner M; 2017	RCT; This study developed and evaluated a video game aimed at introducing students to the operating room, focusing on practical skills such as getting equipped, interacting with patients and understanding how to move within the surgical environment. Participants played the game before their first real operating room experience and afterward, they completed a questionnaire on their subjective experience of the surgical study block.	The questionnaire comprised four scales, i.e. fear to make mistakes, perceived knowledge, perceived errors committed and adequate attitude.	Participants in the intervention group showed statistically significant higher scores on all four outcomes measured.
Venipuncture	Jung EY, Park DK, Lee YH, Jo HS, Lim YS, Park RW; 2012	RCT; The study evaluated the effectiveness of practical exercises using an intravenous simulator with virtual reality and haptics technologies. Participants in the intervention groups trained with either the virtual reality/haptics simulator alone or with both the simulator and a traditional arm model, while the control group used a traditional arm model for venipuncture training.	An overall score for performing the procedures, the success rate of venipuncture, the time taken to complete the task and satisfaction with training materials.	Intervention group 2 had the highest score for procedure performance and the shortest task time, while intervention group 1 had the highest venipuncture success rate. Intervention group 1 showed the highest satisfaction with the effectiveness of the training.

### Mobile applications

This category includes mobile applications for ultrasound imaging or sports injury diagnoses and mobile applications for virtual surgical procedures or task simulations.

Researchers in the included studies have used mobile applications to enhance training in domains such as surgical procedures and respiratory assessment. Mobile applications provided video-based learning, interactive modules, quizzes and step-by-step guidance to improve practical skills, procedural knowledge retention and user satisfaction.

Studies showed positive results, with participants using apps outperforming control groups in practical skill assessments, cognitive learning and satisfaction, although some studies reported no significant differences in knowledge acquisition. Studies showed that mobile applications facilitate learning by providing on-demand access to practical content regardless of location.

Fields of application included carpal tunnel release, cardiopulmonary resuscitation, cardiovascular examination, intramedullary femoral nailing, respiratory system physical assessment, sonography and palpation and urinary catheterization. An overview is presented in Table 4.

**Table 4:** Mobile Applications

Area of application	Author(s); year	Intervention	Outcome(s)	Results
Basic practical nursing skills	Kim H, Suh EE; 2018	RCT; The study evaluated the impact of an interactive mobile application (ICNS) on nursing students’ learning outcomes compared to a non-interactive video-based app. The ICNS group used the app for one week, engaging with modules on vital signs, IV injection, gastric gavage, and endotracheal suction, supported by quizzes and 3D simulations. The control group accessed the same content through a video-only app. Both groups completed pre- and post-tests of knowledge, self-efficacy, and OSCE-based skills assessments.	Knowledge, self-efficacy, and nursing skills performance.	The experimental group scored significantly higher than the control group in knowledge (*p* = 0.001) and nursing skills performance (*p* < 0.001); self-efficacy improved significantly within the experimental group (*p* = 0.017) but not significantly between groups.
Basic practical nursing skills	Yang X, Xie RH, Chen S, Yu W, Liao Y, Krewski D, Wen SW; 2019	Quasi-experimental; The study investigated the use of smartphone-based instant messaging video feedback for teaching fundamental nursing skills to undergraduate students. Nursing students recorded their practice sessions on manikin simulators and sent the videos to instructors via WeChat/QQ for evaluation and feedback, while the control group relied on traditional teaching without video submission. Both groups practiced the same skills, including bed making, aseptic procedures, vital signs measurement, oxygen therapy, catheterization, and enema administration, and completed skill-based assignments after class.	Higher scores in the video feedback group for final examination, bed making, aseptic procedure, vital signs measurement, and oxygen therapy; no significant difference for catheterization, enema, and self-efficacy; high satisfaction with the method.	Video feedback group outperformed control group in most skills, with statistically significant differences for 4 of 6 skills; no difference in GSE scores; over 98% of students were satisfied with the method.
Basic surgical skills	Green CA, Kim EH, O’Sullivan PS, Chern H; 2018	Quantitative, descriptive; The study evaluated a home-video–based surgical skills curriculum delivered via a mobile learning platform (Practice XYZ). Learners watched expert demonstration videos, practiced knot-tying and suturing, recorded and uploaded their performances, and engaged in peer review with structured rubrics. Feedback loops and self-reflection were central to the design. The curriculum included 16 exercises, covering skills such as square knots, instrument ties, mattress sutures, subcuticular sutures, and tying under tension. The focus was on learner satisfaction and feasibility of integrating deliberate practice with mobile-supported peer learning in surgical training.	Outcomes measured included learner satisfaction, reported practice time, frequency of rerecording before submission, and qualitative feedback on usability and educational benefits.	Results showed high satisfaction with all stages of the application, frequent deliberate practice (over half practiced at least 10–30 min per skill before filming), and repeated recording (over 80% rerecorded tasks at least twice). Peer review provided 2–3 reviews per submission, and learners valued the platform’s ease of use, feedback timeliness, and accessibility.
Carpal tunnel release	Amer KM, Mur T, Amer K, Ilyas AM; 2017	Quasi-experimental; Participants learned about carpal tunnel release surgery through either a simulation application on tablets or smartphones or a video lecture with slides. Both groups completed a standardized multiple-choice questionnaire on the procedure.	Performance test, overall app satisfaction	Test scores were higher for the group using the application. Students who used the application rated it highly for content validity, quality of graphics, ease of use and usefulness in preparing for surgery.
Cardiopulmonary resuscitation	Annamalai AAMR, Chandran P; 2022	RCT; Basic life support training for 150 medical students was evaluated using either a mobile application with video-based learning or traditional video-based learning alone. Both groups completed a questionnaire, pre-test, post-test and a practical skill assessment on cardiopulmonary resuscitation.	Compressions per cycle, average depth of compressions, number of compressions in two minutes.	The intervention group performed better than the control group in all parameters.
Cardiovascular examination	Bartlett AS, Smith N; 2019	RCT; A cardiopulmonary mobile application was evaluated for its effectiveness in teaching physical therapy skills, including assessing blood pressure, heart rate and pedal pulses. Participants used the app with integrated videos and descriptive information, with some also receiving demonstrations before practicing in a laboratory setting.	Competency examination conducted by three licensed physical therapists.	The mobile application group showed no improvement over the conventional learning group, but no deterioration either. The highest competency scores were shown by the group that combined conventional and mobile application learning methods.
Intramedullary femoral nailing	Sugand K, Mawkin M, Gupte C; 2016	Quasi-experimental; Participants used a mobile-based cognitive simulation app to complete modules on intramedullary femoral nailing, covering patient preparation, femoral canal preparation, nail insertion, proximal locking, distal locking and closure. They repeated the training modules six times to reinforce procedural knowledge and cognitive decision-making.	Overall performance scores on each module attempt, multiple choice quiz scores before and after training and the learning curve across multiple attempts.	Students showed significant improvement in performance scores across all modules. Multiple choice quiz scores also improved from pre-test to post-test, indicating an increase in procedural knowledge and decision-making competence.
Intramuscular injection	Kacaroğlu Vicdan A; 2020	RCT; The study evaluated the effectiveness of Instagram-based mobile learning for teaching nursing students intramuscular injection at the ventrogluteal site. Over five days, students accessed 64 teaching materials including photos, videos, handwritten notes, questionnaires, and live webcasts. The control group received the same content through traditional classroom instruction with demonstrations. Both groups practiced IM injections on models and were assessed by expert nurses. Skills included preparation, correct injection technique, and post-procedure steps.	No statistically significant difference in knowledge or skills between Instagram and classroom groups; both groups achieved high scores immediately and 15 days after training.	Knowledge and skills scores were similar between groups at both time points; for example, Instagram group total skill score 21.83 ± 4.57 vs. classroom group 22.29 ± 4.46 (*p* > 0.05).
Respiratory system physical assessment	Wu PH, Hwang GJ, Su LH, Huang YM; 2012	Quasi-experimental; Trainees used a mobile-supported system with context-aware guidance to conduct physical assessments in a simulated nursing lab, following step-by-step instructions and receiving immediate feedback. The control group performed the same assessments using traditional learning sheets and teaching assistant guidance, without mobile technology.	Learning achievement (pre- and post-tests), skill test scores for accuracy and smoothness of operations, learning attitudes, cognitive load and acceptance of the mobile learning system.	The intervention group scored significantly higher on learning achievement, skill accuracy and smoothness compared to the control group. They also reported lower cognitive load and had positive attitudes toward the mobile learning system.
Sonography and palpation	Fernández-Lao C, Cantarero-Villanueva I, Galiano-Castillo N, Caro-Morán E, Díaz-Rodríguez L, Arroyo-Morales M; 2016	RCT; Participants had free access to a mobile application designed for learning palpation and ultrasound imaging skills, supplementing traditional learning methods. The application included theoretical descriptions, images, drawings and a video demonstrating the palpation procedure.	Theoretical knowledge on palpation and ultrasound imaging as well as practical skills for ultrasound imaging (e.g. positioning the patient and handling the ultrasound probe) and palpation (e.g. positioning the extremity and precision of palpation).	No significant differences were found in the acquisition of theoretical knowledge. The intervention group had significantly higher scores in ultrasound assessment (e.g., positioning of the patient and ultrasound probe) and palpation skills (e.g., direction of palpation contact and patient positioning). In addition, global OSCE scores were higher for the intervention group in both ultrasound assessment and palpation skills.
Sonography	Lozano-Lozano M, Galiano-Castiollo N, Fernández-Lao C, Postigo-Martin P, Álvarez-Salvago F, Arroyo-Morales M, Cantarero-Villanueva I; 2020	RCT; This study assessed the effectiveness of a mobile app in teaching ultrasound imaging for diagnosing sports injuries among physical therapy students. Participants using the app were compared with those using traditional resources and both groups were evaluated on their theoretical and practical knowledge through a questionnaire and OSCE.	Students’ theoretical knowledge and practical competence in ultrasound imaging. Additionally, student satisfaction was measured.	The intervention group outperformed the control group significantly in all OSCE stations and the knowledge test. The mobile app group took more time to identify lesions than the control group.
Sonography	Situ-LaCasse, Acuna J, Huynh D, Amini R, Irving S, Samsel K, Patanwala AE, Biffar DE, Adhikari S; 2021	Quasi-experimental; Four online ultrasound modules were completed by participants, followed by hands-on practice of ultrasound examinations on standardized patients to apply the learned techniques. The learning format incorporated didactic modules, video demonstrations and quizzes on ultrasound methods and anatomy.	Performance scores, quiz scores on module content and correlations between these two factors.	Students who completed all modules and quizzes scored higher on the assessments. A significant positive correlation was found between quiz scores and performance scores, though not consistently across all individual modules.
Urinary catheterization	Lee NJ, Chae SM, Kim H, Lee JH, Min HJ, Park DE; 2016	RCT; Participants in the intervention group were provided with a mobile-based video clip on urinary catheterization, which they could download and watch repeatedly for one week. The study assessed the impact of this mobile-based content on nursing students’ motivation, competency and classroom satisfaction.	Learning motivation, fundamental nursing competence and class satisfaction.	Participants in the intervention group showed significantly higher scores for learning motivation, confidence in practice and class satisfaction compared to the control group. No significant differences in knowledge and skill performance were found between the two groups.

### E-Learning platforms

This category includes instructional video formats, flipped classroom video tutorials and video podcasts and demonstrations.

Researchers in the included studies have used e-learning platforms to support medical training across areas including venipuncture and physical examination. These platforms involved interactive modules, videos, quizzes and virtual patient cases, often compared against traditional learning methods such as seminars, face-to-face lectures or traditional video demonstrations shown in class (non-interactive, non-digital) or hybrid models. E-learning platforms included multiple digital learning methods in one platform, such as blended/hybrid and video-based learning.

Studies indicated improved performance, higher satisfaction and better procedural knowledge retention for participants using e-learning platforms, although some found no significant differences compared to conventional training.

Fields of application included basic practical endocrinology skills, basic practical medical skills, basic practical nursing skills, basic practical physiotherapy skills, basic surgical skills, cardiovascular and abdominal examination, chest tube insertion, laparoscopy, neonatal resuscitation, pediatric basic life support and physical examination. The main characteristics are summarized in Table 5.

**Table 5:** E-Learning Platforms

Area of application	Author(s); year	Intervention	Outcomes	Results
Abdominal examination	Kalet A, Song HS, Sarpel US, Schwartz R, Brenner J, Ark TK, Plass J; 2012	RCT; The study investigates the effect of varying levels of interactivity in a computer-based learning module on abdominal examination, assessing its impact on clinical knowledge and physical examination performance. Participants used one of three versions of the module, completed pre- and post-tests and performed an abdominal examination on a standardized patient.	Knowledge acquisition and performance on the abdominal examination.	No significant difference in knowledge acquisition between the groups could be found. Students in the click condition performed significantly better in the abdominal examination than those in the other conditions.
Basic dental surgical skills	Corrêa L, de Campos AC, Souza SCOM, Novelli MD; 2003	Quantitative, descriptive; The study evaluated a web-based practical course for teaching basic dental surgical skills, including anesthesia, incision and suture techniques. The course used a combination of videos, pictures and texts to instruct participants in these essential oral surgery skills.	Students’ actions during use were tracked and students’ subjective impressions were collected through a questionnaire.	Different approaches to navigating the website were observed and students showed different approaches to going through the course material. While all students were able to conclude the surgery, the quality of incisions and sutures varied, with 30% of students performing inadequately. Students evaluated the web-based format mostly positive, praising the clear user interface and individually selectable learning content. Difficulty to understand the text and the limited content were the most frequent negative remarks.
Basic practical medical skills	Dohle NJ, Machner M, Buchmann M; 2021	Quantitative, descriptive; This study focused on the development of eTutorial formats designed to teach practical skills to medical students. The eTutorials included instructional videos and virtual classrooms covering various medical fields such as internal medicine, surgery, orthopedics and radiology.	Subjective impression of the eTutorial format, quality of implementation, content of the eTutorials, communication with the tutor during the lessons, participation possibilities during virtual class and atmosphere among students regarding the digital learning process.	Students praised the digital hands-on training with direct feedback, the friendly atmosphere, the shared learning space, the flexibility of time and space and the easy integration of quizzes. On the negative side, participants criticized the difficulty of following hygiene guidelines in pandemic situations, the time required, the need for large classrooms, the difficulty of participation and motivation, the difficulty of practical skills training and the reduced interaction among students.
Basic practical medical skills	Heimbach M, Holzmann K, Stein P, Stief L, Berberat PO, Dirmeier M; 2022	Quantitative, descriptive; A Moodle-based digital tutoring learning format was developed and implemented to teach medical students clinical practical skills such as bladder catheter insertion, electrocardiogram evaluation and surgical suturing and knotting. After using the format, participants completed an online questionnaire-based evaluation.	The self-reported, questionnaire-based evaluation included self-assessments of students’ perceived competence, understanding and confidence in the practical skills they practiced.	The digital tutorial learning format was well received by my students. Students rated their own progress as good to very good. Participants also reported that they did not yet feel well prepared to perform the various skills independently.
Basic practical medical skills	Hosny S, Mishriky AM, Youssef M; 2009	Quasi-experimental; Participants engaged in a computer-assisted training session in a clinical skills lab and provided feedback through a questionnaire regarding their experience. The training focused on basic clinical skills.	Satisfaction with the computer-assisted training, quality regarding learning success and suitability for self-learning were explored. Percentage of student failures was compared to previous student cohorts.	Satisfaction with the computer-assisted training was high. Quality of the product regarding learning success was considered good to excellent. Suitability for self-learning was rated high among faculty members, while students considered the training suitable for self-learning after modification. Percentage of students’ failures was reduced after implementation of the computer-assisted training compared to previous three years.
Basic practical medical skills	Kwant KJ, Custers EJFM, Jongen-Hermus FJ, Kluijtmans M; 2015	Quasi-experimental; The aim was to assess whether interactive e-modules improve students’ performance on practical skills exams compared to traditional text-based preparation. Participants in the intervention group used e-modules with interactive content, while the control group relied on text-based preparation and both groups were assessed during OSCEs.	Scores on OSCE stations as assessed on a five-point scale.	Students who prepared using the e-modules scored significantly higher on the OSCE stations compared to those who prepared using text-based materials.
Basic practical medical skills	Potomkova J, Mihal V, Zapletalova J, Subova D; 2010	Quantitative, descriptive; Evidence-based practice was integrated into bedside teaching in pediatrics, supported by an e-learning platform that provided tutorials and interactive search skills sessions. Students engaged in bedside teaching with real clinical cases, complemented by online activities focused on clinical decision-making and literature interpretation.	Students’ knowledge and skills in evidence-based practice, assessed through feedback and evaluations of their clinical case presentations and search skills.	The study reported positive feedback from 85% of the students, with improvements in their ability to search and apply evidence in clinical practice. There was some criticism regarding the excessive workload, but the overall satisfaction with the program was high.
Basic practical midwifery skills	Ujoh F, Dzunic-Wachilonga A, Noor R, Gusa V, Ape-aii R, Ohene I, Bola R, Christilaw J, Hodgins S, Lett R; 2024	RCT; The study evaluated whether digital delivery of the FIRST midwifery training course via Moodle was as effective as conventional small-group teaching. In the digital arm, students used mobile-based e-learning modules with videos, gamified cases, and quizzes, while the control group received the same content in person. Using a crossover design, all students completed pre- and post-tests, modular quizzes, and an OSCE assessing practical skills, including perineal laceration repair and placenta removal. The digital program was designed to be interactive and accessible offline, supervised by clinical and IT instructors.	Main outcomes included improvement in knowledge and critical thinking (via test scores) and technical competence (via OSCE performance, including both clear pass rates and mean scores).	Both groups improved significantly from pre- to post-test with no significant difference between digital (75.3%) and small-group (75.0%) overall performance (*p* = 0.404). Small-group instruction yielded slightly higher mean OSCE scores for some modules, especially module 3, though the clear pass rates were statistically comparable.
Basic practical nursing skills	Bloomfield JG, Jones A; 2013	Mixed-methods, descriptive; Nursing students’ perceptions and experiences with e-learning as a supplement to traditional clinical skills training were evaluated. The e-learning format included access to current literature, explanatory narratives, video clips, photographs, online journal articles and formative quizzes to teach skills such as hand decontamination, oral medication administration and patient feeding.	Views about the e-learning format, students’ subjective view on the feasibility of the e-learning format regarding clinical skills development and previous experience and self-rated computer expertise.	Students viewed e-learning as a valuable learning format for acquiring clinical skills. The combination of e-learning and conventional teaching was most preferred. Video clips were found to be the most useful.
Basic practical nursing skills	Chevalier S, Paquay M, Krutzen S, Ghuysen A, Stipulante S; 2024	Mixed-methods, descriptive; The study compared the learning impact of shared simulation-based training for plaster and suture procedures among advanced practice nurse (APN) students and medical students. Both groups completed an e-learning module (SimZone Zero) covering equipment and procedural steps, then participated in prebriefing, physician-led demonstrations, and hands-on practice (suturing on pig’s trotters and below-knee plaster casting on peers), followed by debriefing. The training focused on wound suturing and plaster application, integrating digital preparation with supervised practical skill acquisition.	Outcomes measured included knowledge, self-efficacy, satisfaction, self-confidence, and performance in suturing and plastering, along with qualitative feedback on training experience.	No significant differences were found between APNs and MS in performance, satisfaction, knowledge, or self-confidence post-training. Medical students started with higher self-efficacy in some areas, but this gap narrowed after training. Both groups valued the small-group simulation approach and interdisciplinary aspect, though APNs felt less confident in plaster casting and desired more practice.
Basic practical physiotherapy skills	Dell’Isola A, Tschanz M, Wilke J, Brunner F, Bénard MR, van der Esch M, Steultjens MPM; 2023	RCT; The study examined the effectiveness of a self-directed, internet-delivered exercise program for people with knee osteoarthritis compared to an education-only control group. The intervention group accessed a web platform with individualized exercise plans, videos, written instructions, and progression schedules, while the control group received only educational materials on osteoarthritis and physical activity. Outcomes were assessed at baseline and 12 weeks through questionnaires and physical function tests, focusing on improvements in strength, mobility, and functional capacity.	Primary outcome was change in knee pain (measured by the Numeric Rating Scale); secondary outcomes included physical function, quality of life, and self-reported physical activity.	The intervention group showed significantly greater improvements in knee pain and physical function compared to the control group; quality of life also improved more in the intervention group.
Basic practical physiotherapy skills	Preston E, Ada L, Dean CM, Stanton R, Waddington G, Canning C; 2012	RCT; The study evaluated whether adding an e-learning training format with video simulations and explanatory texts to conventional teaching improves practical skills in physiotherapy students. Both groups attended lectures and tutorials, but only one group had access to the online resource, with practical skills assessed at the end of the semester.	Total performance score on a practical examination and scores for specific components such as effectiveness, rationale, explanation and progression of the practical skills.	The intervention group scored 1.6 points higher out of 25 than the control group on the practical exam. They also scored higher regarding effectiveness and rationale components. Additionally, students in the intervention group rated the online resource highly effective and satisfying for helping in exam preparation and improving practical skills.
Basic practical urology skills	Kidess M, Schmid SC, Pollak S, Gschwend JE, Berberat PO, Autenrieth ME; 2021	Quasi-experimental; Individuals took part in a virtual urology practical day by watching instructional videos on various urology topics, followed by answering multiple-choice questions to ensure learning. The curriculum focused on practical urology skills such as digital rectal examination, catheterization and ultrasound imaging.	Student satisfaction with the virtual course, knowledge acquisition and overall student feedback.	Overall feedback was highly positive. Students appreciated the clarity of the videos but noted that virtual teaching cannot completely replace face-to-face learning.
Basic surgical skills	Pelletier F, Torres A, Meloche-Dumas L, Guérard-Poirier N, Kaviana A, Kapralos B, Mercier F, Dubrowski A, Patocskai E; 2023	RCT; Participants practiced suturing skills by recording and uploading procedure videos to an online learning management system, where they received feedback from peers or experts in various formats, such as checklists or free text. The study compared the efficacy of different feedback types and sources on skill acquisition through pre- and post-tests.	Procedural knowledge and psychomotor skills, evaluated using a global rating scale and a checklist. Improvement was assessed from pretest to post-test.	All groups showed significant improvement from pretest to post-test. However, participants who received free-text feedback (from peers or experts) performed significantly better than those who received checklist-based feedback.
Basic surgical skills	Co M, Chu KM; 2020	Quantitative, descriptive; The study assessed the usefulness of a web-based surgical skills learning format that utilized online surgical demonstrations, video case sharing and online teaching. The format focused on basic surgical skills such as suturing, knot tying and simple linear incision.	A standardized questionnaire was used to assess student perceptions and satisfaction with the web-based format.	Students found WSSL easy to follow and just as difficult/easy as conventional teaching for learning basic surgical skills. 27 participants would recommend the web-based learning format to fellow students.
Basic surgical skills	Fehervari M, Das B, Soleimani-Nouri P, Ahmad M, Fadel MG, Deputy M, Morgan C, Burke JR, Mason JD, Nott D, Spalding D; 2022	Quasi-experimental; An online surgical skills course is compared with traditional face-to-face training. Both groups were assessed on skills such as suturing, knot tying and tendon repair, with evaluation by demonstrators and self-reported competency scores.	Competency was assessed for suturing, tendon repair and vascular anastomosis. Self-reported confidence ratings were obtained for suturing, scrubbing, surgical knot tying, tendon repair, instrument recognition and vascular anastomosis.	No significant difference was found between groups regarding the competency ratings. Post course self-rated competency scores for both groups on all technical skills.
Basic surgical skills	McGann KC, Melnyk R, Saba P, Joseph J, Glocker RJ, Ghazi A; 2021	Quasi-experimental; Participants completed e-learning modules and submitted videos of their knot-tying and suturing for peer evaluation as part of an online, hands-on surgical skills elective. The course focused on surgical instrument identification, knot-tying and suturing techniques.	Students’ confidence in performing the surgical skills, overall performance and feedback from both students and faculty members regarding the effectiveness of the elective.	A significant improvement in students’ confidence and skills was found. Students scored highly on knot-tying and suturing. The majority of students felt the elective met or exceeded their expectations.
Laparoscopy	Elrod J, Boettcher J, Vincent D, Schwarz D, Trautmann T, Reinshagen K, Boettcher M; 2022	RCT; Participants in the experimental group received telementoring for laparoscopic suturing, while the control group received in-person training from surgically trained residents. Both groups were trained for a total of 3 h.	Procedure time, knot quality, precision, knot strength and overall knotting performance. Also, the cognitive load was measured using the Surgery Task Load Index.	Both groups improved after the training. No significant differences in performance and cognitive load during the procedure between groups were found.
Laparoscopy	Frascio M, Siri A, Mandolfino F, Chirico M, Vercelli G; 2019	Quasi-experimental; The study presented and evaluated a fully online elective course on minimally invasive surgery for undergraduate medical students. Delivered via the Aulaweb (Moodle-based) platform, the course included four modules with lessons, multimedia content, quizzes, and collaborative activities such as discussion boards and forums. It aimed to improve knowledge of the history, indications, contraindications, risks, common laparoscopic procedures, and recent advances in minimally invasive surgery, while also assessing student satisfaction.	Change in perceived and measured competence, improvement in test scores, and student satisfaction with course design, delivery, and content.	All students showed marked improvement in perceived expertise across all six targeted competencies, with an average 28% increase in knowledge test scores from pre-test (mean 21.7) to post-test (mean 27.3). Pass rate in final assessment was 100%. Overall satisfaction was high (85%), though some students noted the need for clearer course planning and stronger links to practical training.
Ophthalmic procedures	Huang Z, Yang J, Wang H, Chen B, Zheng D, Chen H; 2022	Quasi-experimental; Participants in two intervention groups followed a massive open online course (MOOC) focused on either slit lamp microscope examination or visual acuity testing and used textbooks for previewing the respective skills. The course included lectures, videos and information on various ophthalmic procedures.	Overall performance on the corresponding skill was evaluated using the Direct Observation of Procedural Skills assessment.	Students who completed the MOOC for their skill outperformed students who completed the traditional format. MOOCs were preferred as an additional learning modality rather than a replacement for traditional learning.
Palliative care	Kasar KS; 2023	Quasi-experimental; The study examined the effects of a 14-week online elective course in palliative care on nursing students’ practices and self-efficacy. The program included weekly 30-minute online classes, midterm and final exams, and lectures on symptom management, end-of-life care, and communication. Delivered via the university’s Distance Education Center, the course targeted knowledge and practical competencies in palliative care, with emphasis on patient- and family-centered care.	Students’ self-efficacy and palliative care practices improved after completing the online course.	General Self-Efficacy Scale scores increased from 63.41 ± 11.85 to 68.60 ± 10.09 (*p* < 0.05) and Palliative Care Self-Reported Practices Scale scores increased from 69.43 ± 3.44 to 81.19 ± 6.79 (*p* < 0.05). There was no correlation between self-efficacy and practices before training, but a strong positive correlation afterward (*r* = 0.402, *p* < 0.05).
Pelvic examination	Ronn R, Smith W, Magee B, Hahn PM, Reid RL; 2012	Quantitative, descriptive; The study investigates whether a web-based learning module can effectively prepare first-year medical students for their first pelvic examination. The module included video demonstrations, personalized tutorials and reviews of key procedural steps.	Performance scores, self-reported preparedness using a 5-point Likert scale and volunteer patients’ evaluation of students’ communication, professionalism and confidence.	All participants scored above the passing grade of 50%. 53.3% achieved an honors grade. 88.9% of students reported feeling well-prepared after viewing the training module. No significant difference was found in scores based on gender or the number of times the module was viewed. Scores were slightly lower for students examining a nulliparous volunteer compared to those examining parous volunteers.
Physical examination	Averns H, Maraschiello M, van Melle E, Day A; 2009	RCT; The effectiveness of a web-based teaching module for acquiring clinical hand examination skills was examined. Participants used either the web-based module, a standard book-based learning format or a tutor-led method to develop these skills.	A validated objective structured clinical examination as well as a written knowledge test were used as measurements.	Average total OSCE score was significantly higher for students in the online module group compared to the textbook group. No significant difference was found between students in the online module and the tutor-led groups. The online module group had a significantly higher mean total knowledge score than both control groups.
Physical examination	Barnes ER, Vance BS; 2022	Quasi-experimental; A physical examination skills lab was transitioned from a face-to-face format to an online learning modality that included textbook readings, video demonstrations, recorded lessons and synchronous Q&A sessions. Students completed exam skill demonstrations, received faculty feedback and evaluated their satisfaction with the new learning format.	Physical examination skills performance, satisfaction with the learning modality.	Physical examination skills performance did not differ between learning modalities. Students showed high satisfaction with both formats.
Physical examination	Grundman JA, Wigton RS, Nicol D; 2000	Quantitative, descriptive; This study compares the effectiveness of a web-based multimedia program with a printed manual for teaching physical diagnostic skills of the eye and ear. Participants in the intervention group used the multimedia version with virtual reality movies and interactivity options, while the control group used the printed material, with testing before and after the course.	A knowledge test on physical examination skills of the eye and of the ear including general fact-based questions, images and case studies. Also, students’ acceptance of the format was surveyed.	Students who used the web-based multimedia version format showed higher scores than students who used the printed version of the course material. Students using the web-based format also spent more time on the material than students using the printed version. 78% of the students preferred the multimedia version over the printed version.
Physical examination	Lechner A, Haider SP, Escrihuela Branz P, Paul B, Kashani F, Canis M, Schrötzlmair F, Sharaf K; 2024	Cohort study, single group; The study investigated medical students’ performance in basic head and neck examination after completing an asynchronous online course. The course included compulsory and optional learning modules with instructional videos, image galleries, and supporting materials. Students’ skills were assessed in an OSCE, focusing on cervical lymph node exam, oral/oropharyngeal exam, otoscopy, Weber/Rinne hearing test, and anterior rhinoscopy, and the study also explored students’ self-assessment accuracy regarding their abilities.	Expert evaluation of theoretical and practical performance; comparison of self-assessed and expert-assessed skill levels; analysis of misjudgment patterns.	Students achieved good theoretical knowledge and good to medium practical skills; tasks with higher psychomotor demands showed lower performance; most students self-assessed accurately, with misjudgment mainly due to underestimation; practical skills were better in male students and in those who prepared thoroughly.
Physical examination	Rafai N, Lemos M, Kennes LN, Hawari A, Gerhardt-Szép S, Classen-Linke I; 2016	RCT; An interdisciplinary skills training on head and neck muscle palpation was implemented to enhance the clinical relevance of a preclinical anatomy course. Students attended a lecture, with some groups participating in hands-on skills training and others using an e-learning module to supplement their learning, followed by an OSCE assessment.	Students’ overall performance scores on an OSCE, which tested both their theoretical anatomical knowledge and their practical palpation skills. The evaluation also included student feedback on the perceived clinical relevance of the learning content.	Participation in the skills training significantly improved students’ OSCE scores compared to those who only attended the lecture. Combining the skills training with the e-module resulted in the highest learning outcomes, while the e-module alone had a smaller effect.
Psychomotor examination and treatment	van Duijn AJ, Swanick K, Donald EK; 2015	Quasi-experimental; Participants in the experimental group received online video instructions for psychomotor examination skills and face-to-face training for intervention skills. The control group received the opposite format, with face-to-face instruction for examination skills and video instruction for intervention skills.	Performance was assessed along the Sharp-Purser test, a craniovertebral Rotation Intervertebral motion test, down slide mobilization examination and natural apophyseal glide test.	No statistically significant differences between groups were found on three of four outcomes. The face-to-face group performed better on the natural apophyseal glide test.
Sonography and palpation	Arroyo-Morales, Canterero-Villanueva I, Fernández-Lao C, Guirao-Pineyro M, Castro-Martín, Díaz-Rodríguez L; 2012	RCT; The effectiveness of e-learning as a supplement to traditional training for knee palpation and ultrasound skills was evaluated. Participants accessed either an e-learning website or traditional materials, with both groups completing a structured clinical evaluation and a multiple-choice knowledge test.	Evaluation of skills in palpation such as positioning of patient, direction of palpation contact, localization of specific structure. Evaluation of skills in ultrasound imaging comprising positioning of patient, positioning of ultrasound probe, orientation of ultrasound probe and handling of ultrasound probe. Multiple-choice test on palpation and ultrasound imaging knowledge.	No significant differences between groups could be found on the knowledge acquisition part. Scores were significantly higher in the intervention group than in the control group regarding palpation skills and ultrasound assessment. Students in the intervention group needed less time to palpate but needed more time to obtain a sufficient ultrasound image.
Sonography and palpation	Cantarero-Villanueva I, Fernández-Lao C, Galiano-Castillo N, Castro-Martín E, Díaz-Rodríguez L, Arroyo-Morales M; 2012	RCT; The study assessed the effectiveness of an e-learning format for teaching palpation and ultrasound imaging skills, with participants having free access to a website on musculoskeletal palpation and ultrasound assessment. The e-learning group was compared to a traditional learning group that had access to documents and books on the same topic.	Objective structured clinical evaluation was used to evaluate the educational formats.	The e-learning group scored significantly higher than the control group regarding palpation ability and ultrasound assessment skills.
Surgical knot tying	Brandt MG, Davies ET; 2006	RCT; The study assessed a computer-based self-directed learning approach for teaching the tying of a one-handed surgical reef knot. Participants in the computer-based group used a self-paced presentation, while the conventional group attended a lecture with written and 2D image-based instructions.	Mental rotation test scores, number of knots tied in a training sequence and total number of knots tied across all training sequences.	No significant differences were found between groups on the mental rotation test scores and on the total number of knots tied. The computer-based group improved their knot-tying skills faster than the traditional lecture-based group.
Surgery preparation	Funke K, Bonrath E, Mardin WA, Becker JC, Haier J, Senninger N, Vowinkel T, Hoelzen JP, Mees ST; 2013	Quantitative, descriptive; Participants used a web-based e-learning system alongside traditional face-to-face learning to enhance their surgical knowledge and preparational skills. The system included six virtual patient cases to help develop skills such as history taking, physical examination, diagnosis and treatment planning.	The students’ subjective assessment of effectivity, applicability and acceptance. In addition, performance was measured along factors such as number of correct and complete diagnoses.	Participants showed a significant increase in correctly found diagnoses. Incomplete diagnoses were found less frequently. A significant overall improvement could be observed. Students were satisfied with the web-based e-learning system and preferred blended learning to conventional teaching.
Syringe pump use	Grundgeiger T, Ertle F, Diethei D, Mengelkamp C, Held V; 2023	RCT; This research compares the effectiveness of an e-learning format with hands-on training in syringe pump usage. Participants were either trained using the e-learning program only or with the addition of hands-on practice and their skills were assessed after training and again two weeks later.	Overall performance with the pump, knowledge about using the pump and self-reported confidence in using the pump.	No significant differences could be found between groups.
Urinary catheterization	Öztürk D, Dinc L; 2014	Quasi-experimental; An evaluation was conducted to determine the effect of a web-based education format on nursing students’ urinary catheterization skills. Participants completed an online course with instructional materials, videos and a knowledge test, followed by hands-on practice, while the control group received traditional classroom instruction.	Knowledge of urinary catheterization and skill performance.	No significant difference in knowledge scores between the experimental and control groups was found. The intervention group performed significantly better regarding the urinary catheterization skills test.
X-Ray	Ackermann O, Siemann H, Schwarting T, Ruchholtz S; 2010	RCT; The test group practiced radiologic diagnosis for 4–6 h using a newly developed educational software, while the comparison group used conventional educational materials. Both groups were assessed in a series of tests using 30 pathological and 200 normal radiographs.	The time required for evaluation, the number of correctly diagnosed radiographs and the frequency of false-positive pathology were measured. A subjective self-assessment of the ability to evaluate radiographs and completion of the radiology course was also obtained.	The intervention group performed better than the control group on all parameters. No significant correlation was found between performance and subjective self-assessment or passing the radiology course.
Venipuncture	Bodelle B, Sziegoleit A; 2009	RCT; The study assessed the effectiveness of a web-based training program for teaching venipuncture skills, where participants completed a self-assessment and forced-choice tests on venous blood collection. The web-based group was compared with a conventional teacher-led group and both groups performed a practical skills test by collecting blood samples from a test patient.	A self-assessment on performance, a knowledge test.	The intervention group performed better on the knowledge tests and scored higher on the skills test than the control group. Also, intervention group participants needed less time for the venipuncture procedure.

### Video-based learning tools

This category includes instructional video formats, flipped classroom video tutorials and video podcasts and demonstrations.

Researchers in the included studies have used video-based learning methods to teach a wide range of practical skills, including surgical techniques and laparoscopy. These systems involved instructional videos, sometimes with narration, interactive elements or feedback. They were frequently assessed against conventional teaching methods, such as paper-based learning materials, non-digital practical demonstrations using physical models or mannequins or peer-led training.

Studies generally showed that video-based learning can improve skill acquisition, confidence and satisfaction, though it may not always outperform live instruction or peer-led training, especially for complex tasks or long-term retention.

Fields of application included basic practical endocrinology skills, basic practical medical skills, basic practical nursing skills, basic practical physiotherapy skills, basic surgical skills, cardiovascular and abdominal examination, chest tube insertion, neonatal resuscitation, pediatric basic life support and physical examination. Table 6 gives an overview.

**Table 6:** Video-Based Learning Tools

Area of application	Author(s); year	Intervention	Outcome(s)	Results
Basic practical endocrinology skills	Hibbert EJ, Lambert T, Carter JN, Learoyd DL, Twigg S, Clarke S; 2013	RCT; The effectiveness of a supplemental on-demand video-based training program in teaching endocrinology clinical skills was evaluated. The program includes video-based training on history taking in diabetes, examination for lower limb complications and thyroid disease signs, compared to traditional live demonstrations.	An OSCE-format using performance scores was used to assess students’ skills on the practical skills.	For both diabetes related assessment tasks, students in the intervention group performed better than students in the control group. No significant group differences were found regarding the thyroid examination performance. Significantly more students in the intervention group were rated as globally competent in each skill compared to the control group.
Basic practical medical skills	Maloney S, Storr M, Morgan P, Ilic D; 2013	RCT; Participants supplemented their regular practical class tutoring by producing self-made videos demonstrating clinical skills, which they submitted online for tutor feedback. Both groups took part in an OSCE at the end of the semester, with one group completing the video task and the other not.	Skill performance during the OSCE and students’ perceptions of the self-video process.	The intervention group scored significantly higher in the OSCE compared to the control group Students identified that the self-video process improve their clinical performance and confidence.
Basic practical medical skills	Reed T, Pirotte M, McHugh M, Oh L, Lovett S, Hoyt AE, Quinones D, Adams W, Gruener G, McGaghie WC; 2016	Quasi-experimental; Medical students completed asynchronous video tutorials and quizzes on six core medicine clinical skills, followed by hands-on practice sessions with one-on-one instructor feedback. Their performance was assessed through pre- and post-tests until they achieved the minimum passing standard.	Students’ performance on pretests, post-tests and retention tests, based on skill-specific checklists. Skill retention was measured by re-evaluating a subset of students 1 to 9 months after the intervention.	Significant improvement in student performance across all six skills from pretest to post-test. In the retention test, 98% of students scored at or above minimum passing standards, demonstrating no significant decrease in performance over time.
Basic practical nursing skills	Ali NS, John B; 2019	Quasi-experimental; Three groups learned practical nursing skills (hand washing, surgical bed making, nasogastric tube feeding) using different methods: self-paced online interactive videos, in-class video demonstrations or a demonstration-only approach. The intervention used online interactive video recordings to teach these skills.	Competency assessment checklist for nursing practice skills, satisfaction questionnaire, subject feedback on teaching methods.	No differences in competency skills were found among the three instructional methods. Satisfaction scores were highest for the online, self-paced, interactive video learning method.
Basic practical nursing skills	Arslan GG, Ozden D, Goktuna G, Ayik C; 2018	RCT; The study examined how the timing of instructional videos affects student satisfaction in basic nursing skills training. One group watched the video before practicing in the skills lab, while the other group watched it afterward, with both groups covering skills like wound care.	The “Assessment of Satisfaction Questionnaire for the Interactive Video-Based Skills Learning” was used for measuring satisfaction-based data.	Mean Scores of both groups weren’t different from another. Both groups scored high on total satisfaction, indicating a high average satisfaction in both conditions.
Basic practical nursing skills	Barisone M, Bagnasco A, Aleo G, Catania G, Bona M, Scaglia SG, Zanini M, Timmins F, Sasso Loredana; 2019	Qualitative, descriptive; A web-based video learning format was evaluated for its feasibility and effectiveness in teaching clinical nursing skills, including urinary catheter insertion, nasogastric tube insertion, blood sampling and peripheral intravenous line placement. The study assessed students’ perceptions and the applicability of this digital approach in nursing education.	Demographics, knowledge of and attitudes toward video instructional methods, subjective evaluation of video instructional methods.	The digital service proved to be effective for the acquisition of the clinical nursing skills.
Basic practical physiotherapy skills	Hurst KM; 2016	Qualitative, descriptive; Students used the video podcast learning format alongside conventional courses in musculoskeletal and neurological domains. The video podcasts focused on physiotherapy clinical skills such as physical examination and history taking.	Focus groups were used to assess students’ perceptions of the video podcast learning format regarding the format’s impact on their subjective self-efficacy, procedural knowledge, technical ability to perform a skill and clinical reasoning.	Students found the video podcast format helpful for reviewing practical exams and praised the versatility and audio-visual nature of the video podcasts. The ability to watch and practice at the same time was considered convenient.
Basic surgical skills	Lu EL, Harris MK, Gao TZ, Good L, Harris DP, Renton D; 2022	Quantitative, descriptive; This study assessed the impact of combining near-peer teaching with flipped classroom methods to improve students’ confidence in basic surgical skills. Participants learned suturing and knot-tying techniques through publicly available YouTube videos and practiced them in live, supervised sessions.	Students’ self-perceived confidence in performing surgical techniques, interest in pursuing a career in surgery and preparedness to begin their surgical clerkship.	A significant increase in students’ confidence across all skills was found. All students expressed a stronger interest in pursuing surgical careers and reported feeling more prepared for their clerkship.
Basic surgical skills	Mayorga-Young D, LaGuardia J, Sweitzer K, Chikotix R, Butterfield J, Ali-Khan S, Ahmed A, Leckenby J; 2024	Quasi-experimental; The study assessed the feasibility and effectiveness of video-based education with individualized review for teaching medical students basic suturing skills. Participants watched 80 min of standardized instructional videos, practiced on synthetic skin, recorded their performance, and received synchronous feedback from a plastic surgery resident via Zoom before repeating the task. Practical skills included simple interrupted, running simple, and subcuticular suturing techniques, along with knot-tying methods.	Improvement in suturing steps completed, economy of time and motion, and proportion of students achieving proficiency; analysis also considered year in school, prior surgical clerkship, and interest in surgery.	Significant improvement in suturing steps (4.27 to 4.80, *p* = 0.028) and economy of time and motion (2.60 to 3.20, *p* = 0.029) overall; 5 of 8 previously non-proficient students achieved proficiency post-feedback; students interested in surgery showed higher post-feedback economy of motion scores.
Basic surgical skills	Nousiainen M, Brydges R, Backstein D, Dubrowski A; 2008	RCT; This research compared expert instruction with computer-based interactive and non-interactive video instruction formats for teaching suturing and knot-tying skills. Participants practiced these skills after watching instructional videos, with some receiving additional expert feedback before post-tests and retention assessments.	Motion efficiency including number of movements, total time for completion and total path distance.	All groups showed significant improvement in skill performance from pre- to post-test and retention test. No significant differences between groups were found.
Basic surgical skills	Xeroulis GJ, Park J, Moulton CA, Reznick RK, LeBlanc V, Dubrowski A; 2007	RCT; Trainees were assigned to three groups: one used a computer-based video instruction program for learning suturing and knot-tying skills, another received real-time expert feedback during practice and the third received only post-trial feedback. The control group viewed an instructional video but practiced without any additional guidance or feedback.	An expert-based assessment on various aspects of performance, using a global rating scale and a hand motion analysis, where a computer analyzed efficiency in hand movements and total time taken.	All intervention groups showed significantly better performance than the control group. At the one-month retention test, only the computer-based video instruction learning format retained their superior performance compared to the control.
Basic suturing skills	Raythatha J, Hameed A, Lee T, Yuen L, Nahm CB, Pang T, Pleass H; 2024	RCT; The study investigated whether supplementing face-to-face teaching with a narrated instructional video improved acquisition and retention of single-handed surgical knot-tying skills. The intervention group viewed the video before and after a 1.5-hour workshop with live demonstration and one-on-one tuition, while the control group only accessed the video after follow-up. Both groups attended the same workshop and were assessed on knot quality and quantity immediately after and four weeks later.	Number of knots tied in 1 min and global quality score of knots at initial and 4-week follow-up, plus self-reported confidence, stress, and perceived skill retention.	Intervention group tied significantly more knots initially, had better knot quality at follow-up, reported higher confidence in skill retention, and experienced less stress than control group; skill retention trend favored the intervention group but was not statistically significant for knot quantity at follow-up.
Cardiovascular and abdominal examination	O’Donovan J, Ahn R, Nelson BD, Kagan C, Burke TF; 2016	RCT; This study assessed the feasibility and impact of using low-cost Android tablets preloaded with instructional videos to teach cardiovascular and abdominal examination skills to third-year medical students in Kenya. Participants used the tablets for self-directed learning over three weeks, with performance evaluated through pre- and post-study OSCE assessments.	Students’ overall performance as assessed by OSCE scores.	The intervention group showed significantly higher improvements in OSCE scores compared to the control group in both cardiovascular and abdominal assessments.
Chest tube insertion	Saun TJ, Odorizzi S, Yeung C, Johnson M, Bandiera G, Dev SP; 2017	RCT; Participants viewed either a peer-reviewed instructional video or a recorded didactic lecture on chest tube insertion, covering procedure steps, equipment use and potential complications. Afterward, both groups performed chest tube insertions on cadavers in a structured OSCE format.	Procedural performance scores based on an OSCE, pre- and post-questionnaire scores on knowledge and self-reported confidence in performing the procedure.	Both groups performed similarly on the OSCE, with no significant differences. The intervention group showed a significantly higher improvement in knowledge on the post-intervention questionnaire compared to the control group.
Laparoscopy	Schmidt MW, Kowalewski KF, Trent SM, Benner L, Müller-Stich BP, Nickel F; 2020	RCT; First-person perspective instructional videos were compared to endoscopic-view-only videos to assess their effectiveness in teaching laparoscopic skills. Participants practiced suturing and knot tying on a box trainer after engaging with the assigned video format.	Time taken to reach proficiency, the number of attempts needed, quality of knot tying and participant feedback on training preferences.	Both groups showed significant improvements, achieving similar performance levels without a significant difference in time to reach proficiency. The first-person perspective did not lead to better performance but was positively perceived by participants.
Neonatal resuscitation	Yoosoof F, Liyanage I, de Silva R, Samaraweera S; 2022	Quasi-experimental; Trainees acquired procedural knowledge through either short, narrated demonstration videos or text-and-image-based PowerPoint slides before attending a skills lab on newborn resuscitation. Both groups received the same conceptual knowledge, participated in hands-on simulation training and completed pre- and post-tests to assess learning outcomes.	Knowledge acquisition, skill performance and students’ subjective perception.	The intervention group showed significantly better performance in procedural skills compared to the control group. Both groups demonstrated gains in post-training, but only the intervention group showed statistically significant gains. Students in the intervention group reported higher engagement and confidence as well as ease of understanding with the preparatory material.
Neuromuscular examination	Villar-Quiles RN, Bouaoud J, Foy JP, Behin A, Masingue M, Nguyen TM, Iniesto M, Stojkovic T, Idbaih A; 2025	Quasi-experimental; The study developed and evaluated an educational video for teaching medical students the neuromuscular examination, including pathological signs and diagnostic orientation. Students watched the 18-minute video during their neurology clerkship and completed pre- and post-video quizzes plus a usefulness survey. A control group viewed a different video (oral and maxillofacial examination) and completed the same quizzes. Skills taught included inspection, gait analysis, segmental muscle testing, joint contracture examination, recognition of pathological signs, and semiological reasoning.	Significant improvement in NM knowledge and skills in the intervention group (mean score increase from 6.36 to 9.12, *p* < 0.01), improved self-reported preparedness and confidence, and high perceived usefulness of the video.	Intervention group showed significant gains in NM quiz scores and in questions targeting both specific examination manoeuvres and etiological orientation, while the control group improved only on the OMS quiz; self-rated preparedness rose from 5.0 to 7.4/10 and perceived usefulness was 8.73/10
Pediatric basic life support	Stephan F, Groetschel H, Büschner AK, Serdar D, Groes KA, Büscher R; 2018	RCT; Medical students were trained in pediatric basic life support either through peer-led hands-on training with an infant manikin or by watching a 12-minute instructional video. Both groups were assessed immediately after training using an OSCE and re-evaluated at the end of the semester.	OSCE performance scores, including immediate and delayed assessments and a global performance rating.	The peer teaching group performed significantly better on the OSCE both immediately after the initial training and at the end of the semester. Students trained by peer instruction scored higher on practical skills, indicating better retention compared to the video demonstration group.
Physical examination	Behrends M, Stiller G, Dudzinska A, Schneidewind S; 2016	Quantitative, descriptive; The study examined whether instructional videos with or without narration are more effective for teaching physical examination skills to medical students. Different video formats were compared to assess their impact on skill acquisition and learning outcomes.	Quantitative use of the videos, students’ learning behavior, students’ self-rated learning success and estimation of the didactic quality of the videos.	More students used the uncommented instructional videos than the commented. Regarding the learning success, students preferred the uncommented over the commented videos. In respect to the didactic quality, the majority of the students found both video formats equally suitable.

### Serious games and gamified learning

This category includes gamified flipped classroom settings and serious virtual games (e.g. for blood transfusion procedures).

Researchers in the included studies have used serious games and gamified learning to teach various practical skills, such as alginate mixing and emergency room management. These approaches involved interactive elements like quizzes, leaderboards, simulations and virtual environments, aiming to increase learning motivation, engagement and familiarization with the learning method.

Studies generally showed positive results, with participants reporting higher knowledge gains, confidence, satisfaction and improved practical performance compared to established teaching methods such as traditional in-class video demonstrations or small-group tutorials.

Fields of application included alginate mixing, basic practical nursing skills, chest tube insertion, emergency room-related practical skill. A summary of the information can be found in Table 7.

**Table 7:** Serious Games and Gamified Learning

Area of application	Author(s); year	Intervention	Outcome(s)	Results
Alginate mixing	Hannig A, Lemos M, Spreckelsen C, Ohnesorge-Radtke U, Rafai N; 2013	RCT; This study evaluates a serious virtual game designed to train rhythmic and periodic motor skills for alginate mixing compared to a traditional workshop. All participants were pre- and post-tested on their alginate mixing skills.	Usability of the serious game, self-reported skills, knowledge of the process and subjective impressions of the virtual game.	A positive learning effect was found for both conditions. The intervention group showed a higher knowledge again from pre- to post-test. Students indicated high satisfaction with the serious game in terms of learning motivation and usability.
Basic practical nursing skills	Butt AL, Kardong-Edgren S, Ellertson A; 2018	Mixed-methods, descriptive; The study investigated the usability and effectiveness of a game-based VR system with haptic gloves for practicing urinary catheterization, compared with traditional task-trainer practice. The VR group trained for one hour using Oculus Rift and haptic gloves, while the control group practiced on a physical task trainer with supervision and feedback. Two weeks later, both groups demonstrated urinary catheterization on a task trainer, evaluated with a checklist by blinded reviewers. The VR system provided gamified features like goals, scoring, and feedback, while training emphasized sterile technique and correct procedural steps.	Usability score, engagement, enjoyment, practice duration, number of completed procedures, and follow-up skill performance.	VR group rated usability as acceptable (mean SUS score 72.5), found the system engaging and enjoyable, practiced significantly longer (mean 25.3 vs. 14.9 min, *p* = 0.001) and more frequently (mean 3.0 vs. 1.8 procedures, *p* < 0.001) than the control group. Follow-up pass rates for sterile technique were identical at 60% in both groups.
Basic practical nursing skills	Chang CY, Chung MH, Yang JC; 2022	Quasi-experimental; The study evaluated an online game-based learning format combined with a watch-summarized-question strategy for teaching clinical skills, such as in-patient intubation and sputum suction. The online game provided interactive learning materials, while the watch-summarized-question strategy involved watching lecture videos, summarizing key points and generating questions.	Pre- and post-tests were used to assess students’ learning achievement of sputum suction skills, self-efficacy, learning engagement and learning satisfaction.	The intervention group scored significantly higher on learning achievement, self-efficacy, learning engagement and learning satisfaction compared to the control group.
Basic practical nursing skills	Elzeky MEH, Elhabashy HMM, Ali WGM, Allam SME; 2022	RCT; This study compared the effectiveness of a gamified flipped classroom format and a routine flipped classroom approach for teaching nursing skills. It involved interactive elements like quizzes, badges and leaderboards in the gamified format, while the routine format consisted of instructional videos, peer evaluations and in-class demonstrations.	Students’ self-confidence, skills knowledge, intensity of preparation, motivation and general skills performance.	A significant difference between groups was found for self-confidence, knowledge, intensity of preparation and motivation. No difference was found between the groups on general skill performance.
Basic practical nursing skills	Koivisto J-M, Buure T, Engblom J, Rosqvist K, Haavisto E; 2024	Quasi-experimental; The study evaluated the effectiveness of a 3D simulation game for improving surgical nursing knowledge. Nursing students in the intervention group played through five interactive patient scenarios covering pre- and postoperative assessments, complication management, and interventions, while the control group studied the same content via self-study material. Both groups completed the Surgical Nursing Knowledge test before and after. Skills trained included patient assessment, pain management, blood transfusion, and application of NEWS criteria.	Surgical nursing knowledge improvement as measured by the SNK test; changes in specific subareas of knowledge.	Both groups improved, but the experimental group showed a greater overall increase in knowledge; significant improvements in several subareas for the experimental group, though knowledge decreased in some areas.
Basic practical nursing skills	Tan AJQ, Lee CCS, Lin PY, Cooper S, Lau LST, Chua WL, Liaw SY; 2017	RCT; Engaging with a serious game on blood transfusion, participants simulated pre-transfusion, transfusion and post-transfusion procedures within a virtual hospital setting. Receiving only traditional lectures and skills lab training, the control group did not participate in the serious game until after the study.	Knowledge and confidence scores and performance scores in a simulation-based assessment. Additionally, participants in the intervention group provided feedback on their perceptions of the serious game.	The intervention group showed significant improvement in both knowledge and confidence scores compared to the control group. Although the experimental group scored higher in the simulation performance test, the difference was not statistically significant. Participants positively rated the serious game.
Cardiopulmonary resuscitation	Boada I, Rodriguez-Benitez A, Garcia-Gonzalez JM, Olivet J, Carreras V, Sbert M; 2015	RCT; The intervention group used LISSA, a 3D serious-game environment designed to teach CPR, combined with standard self-directed theory learning. Both groups participated in laboratory sessions with a mannequin, but only the intervention group engaged with the LISSA game.	CPR performance scores, assessed with a structured checklist covering required procedural steps.	Students in the LISSA group achieved significantly higher CPR performance scores compared to the control group, indicating superior learning acquisition.
Chest tube insertion	Haubruck P, Nickel F, Ober J, Walker T, Bergdolt C, Friedrich M, Müller-Stich BP, Forchenheim F, Fischer C, Schmidmaier G, Tanner MC; 2018	RCT; This study tests the effectiveness of the Touch Surgery app in teaching emergency chest tube insertion to medical students, using a serious gaming approach. Students practiced with the chest tube insertion module of the app, compared to a control group practicing thoracocentesis.	Participants’ performance in chest tube insertion on a porcine model was rated on-site and via video recordings. Additionally, participants filled out an individual questionnaire for self-evaluation about their individual training level, previous experiences and subjective impressions of the procedure.	The intervention group performed significantly better than the control group and also demonstrated significantly improved time and motion economy, required significantly less assistance and were more confident in instrument handling.
Emergency room-related practical skills	Masoumian Hosseini M, Manzari ZS, Gazerani A, Masoumian Hosseini ST, Gazerani A, Rohaninasab M; 2023	Quasi-experimental; The study evaluated whether gamified surgical sets improve surgical technology students’ ability to recognize and arrange instruments and support performance retention. The intervention group used a computer-based drag-and-drop game to practice positioning six types of surgical sets on a virtual operating table, with scoring, leaderboards, and feedback. The control group used conventional self-directed training without gamification. Both groups completed OSCEs in non-clinical and clinical settings and a 3-month follow-up test. Skills targeted included instrument recognition, correct placement on the operating table, and understanding their surgical use.	Outcomes were performance in OSCE tests at three time points: non-clinical, clinical, and three months later, measuring accuracy, arrangement skills, and retention.	Game-based learning groups performed significantly better than traditional learning groups in all OSCEs, with retention maintained at three months, unlike the decline seen in the traditional group.
Emergency room-related practical skills	Rogers L, Miller C, Firmin S; 2012	Quantitative, descriptive; Students participated in the Critical Life simulation within Second Life, working in teams to manage six emergency room scenarios involving tasks like respiratory examinations, medication administration and patient assessment. The virtual simulation provided interactive features, including text, audio and tactile feedback, to enhance the learning experience.	Participants were asked if they felt the simulation was realistic and about their subjective learning experience.	Participants rated the learning experience with the clinical simulation platform as positive and realistic and stated that Critical Life would assist them in learning technical and non-technical skills.
Tracheostomy	Bayram S, Caliskan N; 2019	RCT; The study evaluated the effectiveness of a game-based VR mobile application for teaching tracheostomy care to nursing students. Both groups received standard theoretical and practical training, but the intervention group also used the VR app for seven days, practicing as often as they wished. The app, developed with Adobe Flash, guided students through interactive steps for suctioning, cannula cleaning, and skin care. All students completed a knowledge test and skill assessments (OSCE) before and after the intervention.	Primary outcomes were knowledge test scores and skill performance scores for the three practical skills. The time taken to complete each skill was also recorded.	Students in the experimental group showed significantly higher final scores in suctioning and peristomal skin care skills compared to the control group (*p* = 0.017 and *p* = 0.003, respectively). The knowledge scores increased in both groups but without statistically significant differences. The experimental group generally performed the procedures more efficiently.

### Telecommunication tools

This category includes videoconferencing tools for real-time feedback and telementoring as well as telesimulation platforms.

Researchers in the included studies have used telecommunication tools like Microsoft Teams (Redmond, WA), WebEx (San José, CA) and live-streamed sessions to teach practical skills remotely, including suturing and neurodynamic procedures. These tools involved real-time feedback, visual comparisons with experts and interactive Q&A, allowing participants to practice skills at home or in remote environments.

Studies generally showed significant improvements in practical skill performance and satisfaction across both remote and in-person groups, with some studies even showing the intervention group outperforming in-person learners in certain tasks.

Fields of application included basic surgical skills, basic suturing skills, endoscopy, liver exam, mirror laryngoscopy, physical examination and history taking, physiotherapeutic neurodynamic testing and surgical suturing. Table 8 is presenting an overview.

**Table 8:** Telecommunication Tools

Area of application	Author(s); year	Intervention	Outcome(s)	Results
Arthrocentesis	Thomson A, Larson G, Moeller J, Soucy Z, Zapata I, Mason NL; 2024	RCT; The study compared remote versus in-person instruction for teaching ultrasound-guided knee arthrocentesis on formalin-embalmed cadavers. All participants watched a 20-minute orientation video, after which the intervention group received 25 min of remote one-on-one instruction via Zoom with multi-camera feedback, while the control group received the same training in person. Both groups practiced and were assessed on cadaver knees. Skills taught included probe handling, identification of anatomical landmarks and effusions, and needle guidance for aspiration.	Primary outcome: change in self-reported confidence; secondary outcomes: procedural competency, knowledge, and satisfaction with training.	Both groups showed significant increases in self-confidence across all items, with no significant difference between groups except for “knowledge of instruments,” which was higher in the in-person group. Skills performance, time to aspiration, and number of successful aspirations were otherwise similar.
Basic practical medical skills	Gamble C, Oatham A, Parikh R; 2023	Quantitative, descriptive; The study evaluated the Virtual OSCE Buddy Scheme (VOBS), a national near-peer online teaching program for final-year medical students during COVID-19. Small groups were paired with junior doctor buddies and attended weekly online sessions over 3–5 months, covering OSCE-related skills such as history-taking, communication, clinical reasoning, examination, and procedures. Delivered via platforms like Zoom and MS Teams, the program aimed to improve skills, confidence, and explore whether virtual OSCE teaching could replace or complement face-to-face formats in the future.	Self-reported improvement in OSCE-related knowledge, skills, and confidence for students, and in teaching skills, communication, lesson planning, and teaching confidence for buddies.	In 2020/21, 96% of students reported increased confidence, with high improvement in communication (90%), diagnosis/clinical reasoning (89%), and history-taking (86%), but lower improvement in examination (31%) and procedural skills (15%). In 2021/22, 90% felt more prepared for OSCEs, with improvement in communication (87%), diagnosis/clinical reasoning (84%), history-taking (83%), examination (40%), and procedural skills (24%). Buddies in both years reported high gains in teaching skills (87–90%), lesson planning (88–92%), and teaching confidence (91–93%).
Basic practical nursing skills	Karabacak Ü, Çelik HY; 2025	Mixed-methods, descriptive; The study evaluated the effectiveness of remote learning for teaching blood pressure measurement to nursing students. The experimental group practiced at home with a family member as a simulated patient under real-time Zoom guidance from an educator, while the control group learned from an evidence-based prerecorded video and practiced independently. Both groups first attended a theoretical session and later underwent online and face-to-face assessments using the same checklist. The training targeted accurate execution of all procedural steps, including identification of Korotkoff sounds.	Skill performance scores from the Blood Pressure Measurement Skill Checklist and qualitative feedback on emotions, learning, and self-confidence regarding the remote learning experience.	No significant difference between groups in the first assessment (*p* = 0.440), but in the second assessment the experimental group scored significantly higher than the control group (*p* = 0.001). Qualitative data showed higher engagement, better perceived learning, and greater confidence in the experimental group.
Basic practical osteopathic skills	Berenbeim G, Metzler I, Lewis D, Jie C; 2022	Mixed-methods, descriptive; The study evaluated the effectiveness of a virtual practical exam (vPE) in osteopathic manipulative medicine as an alternative to traditional in-person exams during the COVID-19 pandemic. Students completed the vPE via Zoom, using props to simulate physical exams and treatments while verbalizing steps, identifying landmarks, and demonstrating techniques. The intervention also included preparatory instructional videos and Q&A sessions. Skills assessed included procedural OMM techniques, psychomotor performance, communication, and diagnostic reasoning.	The outcomes were student perceptions of fairness, effectiveness, skill assessment, and satisfaction with feedback.	The results showed that over 90% of students agreed that the vPE was a fair assessment of their knowledge and over 86% felt it adequately assessed their procedural skills. Most also preferred it over multiple-choice exams and appreciated the feedback received.
Basic surgical skills	Halim F, Candrawinata VS, Widysanto A, Budihardja AS, Wahjoepramono POP, Irawan A, Sudirman T, Christina N, Koerniawan HS, Tobing JFL, Sungono V, Marlina M, Wahjoepramono EJ; 2023	RCT; This study compared first-person-view live-streaming using a head-mounted GoPro Hero 8 with traditional face-to-face demonstrations for teaching simple wound suturing to medical students during their surgical clerkship. In the streaming group, students watched the procedure remotely and asked questions via speakerphone, while the control group observed in person. Both groups received the same demonstration by the same instructor with opportunities for clarification. The digital intervention enabled remote, interactive surgical teaching, focusing on skin closure with simple interrupted sutures.	Pre- and post-intervention DOPS scores, self-reported training frequency, subjective evaluations of teaching quality, skill improvement, and confidence.	Both groups improved significantly; no significant difference in score improvement (delta) between groups, but face-to-face group had significantly higher mean post-test score (86.4 vs. 78.9, *p* = 0.02). Live-streaming group trained more often (*p* = 0.048) and reported higher confidence but rated teaching quality and skill improvement lower.
Basic surgical skills	Nathan A, Fricker M, Georgi M, Patel S, Hang MK, Asif A, Sinha A, Mullins W, Shea J, Hanna N, Monks M, Peprah D, Sharma A, Ninkovic-Hall G, Lamb BW, Kelly J, Sridhar A, Collins J; 2021	RCT; The study compared virtual classroom training (VCT) with face-to-face training (FFT) and computer-based learning (CBL) for teaching medical students basic surgical skills. All groups viewed the same pre-recorded instructional video beforehand. The VCT group participated in a 90-minute interactive live session with expert guidance via BARCO weConnect, while FFT students trained in person and CBL students practiced independently with non-interactive video content. Skills assessed included interrupted suturing and hand-tied knot tying through pre- and post-intervention performance evaluations.	Post-intervention proficiency in suturing and knot tying measured by OSAT, self-reported confidence, perceptions of training quality, and cost/resource use.	VCT was non-inferior to FFT and superior to CBL, FFT was superior to CBL, all groups improved proficiency and confidence, VCT had lower costs and greater instructor time efficiency than FFT.
Basic surgical skills	Pinter ZB, Maróti P, Kopjár E, Gasz B, Duga Z, Rendeki S, Nagy B, Füzesi Z, Schlégl AT; 2022	RCT; A comparison was made between distance learning via Microsoft Teams, where students followed instructional videos and practiced suturing and knotting at home and traditional in-person classes with guided hands-on sessions. Both groups completed pre- and post-tests, followed a standardized curriculum and had their skills evaluated through video assessments.	Students’ improvement in knot tying and suturing scores.	Both distance learning and in-person teaching groups showed significant improvements in their scores. While there was no significant difference in knot tying scores, the distance learning group showed better improvement in suturing scores, particularly in vertical mattress suturing.
Basic surgical skills	Co M, Chung PHY, Chu KM; 2021	Case control; The study compared a web-based surgical skills learning session (WSSL) with traditional face-to-face instruction for medical students. The WSSL group trained via Zoom with live demonstrations and real-time feedback, while the control group received the same training in person. Both groups were later assessed using a modified OSATS on skills such as skin incision, suturing, and surgical knot tying.	The primary outcome was surgical competency, measured using a modified OSATS global rating scale (scored out of 5).	Both groups performed similarly. The mean OSATS score was 4.8/5 for the face-to-face group and 4.7/5 for the WSSL group (*p* = 1), indicating no significant difference. All students were able to perform proper surgical knots.
Basic suturing skills	Zaghal A, Marley C, Rahhal S, Hassanieh J, Saadeh R, El-Rifai A, Qaraqe T, ElBejjani M, Jaafar R, Hoballah JJ; 2022	RCT; The distance learning group participated in interactive telesimulation sessions via WebEx, where an instructor demonstrated suturing steps live, shared instructional videos and provided real-time feedback as students practiced remotely. The face-to-face group received traditional in-person training, including live demonstrations and supervised practice sessions with immediate onsite feedback.	Performance was evaluated using a validated checklist and the objective structured assessment of technical skills global rating tool. Secondary outcomes included students’ satisfaction and confidence.	No significant differences in performance scores between the face-to-face and distance learning groups. Both groups expressed high levels of satisfaction and confidence. However, 28.5% of distance learning participants preferred face-to-face learning due to challenges with remote visualization and feedback.
Endoscopy	Kumagai T, Yamashita J, Morikawa O, Yokoyama K, Fujimaki S, Konishi T; 2008	Quasi-experimental; Trainees participated in remote instruction via a telecommunication interface that enabled real-time visual comparisons between their movements and those of experts. Additionally, they watched instructional videos on surgical instrument use and anatomical structures relevant to endoscopic procedures.	Students’ ability to complete the tasks, subjective evaluations, improvement in posture, instrument handling and insertion angle/depth.	Most students improved their manual skills and were able to complete the tasks effectively post-training. The telecommunication system enabled effective correction of posture and instrument handling through visual comparisons with trainers. The aspiration task was more challenging than the probing task, leading to slightly lower performance scores.
Liver exam	Teitelbaum D, Xie M, issa M, Nelms M, Wintraub L, Leung FH, Nyhof-Young J, Otremba M, Sirianni G, Prucnal K; 2022	Quasi-experimental; Observing a tutor perform a liver exam through a live-streamed, first-person perspective, the intervention group received real-time narration and interacted by asking questions. Watching a pre-recorded third-person video of the same exam, the control group engaged in a group discussion with a preceptor afterward.	Student engagement, comfort with interacting with the tutor, ability to visualize the examination maneuvers, perceived improvement in clinical skills and satisfaction with the session.	Students in the point-of-view group reported higher levels of engagement, comfort with tutor interaction and satisfaction with the session. They felt better able to visualize exam maneuvers and believed their knowledge of clinical skills had improved more significantly than those in the pre-recorded video group.
Mirror laryngoscopy	Krauss F, Giesler M, Offergeld C; 2022	Quasi-experimental; The digital training program involved videoconferencing for live demonstrations, practice sessions and assessments, where students learned mirror examination techniques for otolaryngology. They practiced using instruments at home, applying the skills learned during the online sessions.	Performance scores regarding mirror examination techniques and competency improvement.	A significant increase in performance was found from pre- to post-test. Students in the intervention group reached 90% of maximum score for most examinations by training day four.
Neonatal resuscitation	Mileder LP, Bereiter M, Wegscheider T; 2021	Quantitative, descriptive; The study evaluated the feasibility and effectiveness of telesimulation for neonatal resuscitation training, comparing medical students and neonatal nurses. Participants received a low-fidelity mannequin and neonatal equipment, then joined a one-hour Cisco Webex session covering the resuscitation algorithm with guided practice and feedback. Skills included drying and stimulation, airway opening, bag-valve-mask ventilation, and chest compressions. The study also assessed participants’ knowledge, attitudes, and perceptions of telesimulation.	Feasibility of telesimulation, participants’ perception of its suitability and learning effect, and change in neonatal resuscitation knowledge scores pre- and post-intervention.	Knowledge scores increased significantly from a median of 16/20 to 20/20 overall, with similar improvements in both medical students and nurses; participants rated the session positively but noted potential drawbacks such as technical/logistical challenges and reduced feedback quality compared to in-person training.
Physical examination	Schleicher I, Davids L, Latta N, Kreiß AF, Kreuder J; 2021	Quantitative, descriptive; Students participated in a video conference-based course where tutors demonstrated musculoskeletal examination techniques and students practiced on individuals in their home environment while receiving real-time feedback. The course focused on techniques related to the shoulder, knee, spine, hip, ankle joints and basic neurological exams.	Qualitative feedback from students and tutors on the course’s effectiveness, student participation and the technical feasibility of online teaching for physical examination techniques.	Feedback from both students and tutors was predominantly positive. Some students reported difficulties finding a person to practice on and there were concerns about the limitations of online learning compared to in-person practical sessions.
Physical examination and history taking	Visuddho V, Nugraha D, Melbiarta RR, Rimbun R, Purba AKR, Syafa’ah I, Bakhtiar A, Rejeki PS, Romdhoni AC; 2023	Case control; Medical students engaged in either an online or offline course format, covering theory lectures and skill demonstration sessions on history taking, lung physical examination and heart physical examination. The online group participated via video conferencing platforms, while the offline group received the same training in person, completing practical demonstrations accordingly.	Theory exam scores, skill exam scores, attitude scores and satisfaction ratings across both groups.	Participants in the intervention condition showed significantly higher scores in knowledge and practical skills compared to the offline group. However, the offline group scored higher in attitude and satisfaction.
Physiotherapeutic neurodynamic testing	Villagran I, Rammsy F, Del Valle J, de las Heras SG, Pozo L, Garcia P, Torres G, Varas J, Mandrusiak A, Corvetto M, Fuentes-Cimma J; 2023	Quasi-experimental; Physiotherapy students participated in remote, asynchronous training by watching instructional tutorials, practicing neurodynamic skills independently and submitting recorded performances for instructor feedback. Their learning followed a structured four-step process, including tutorial viewing, video submission, feedback reception and repeated practice across four sessions.	Performance scores from a checklist and rubric, evaluating students’ adherence to technique and overall skill quality.	By the second session, most students met the target standard on the checklist, with quality improvements noted by the third session. Performance leveled off in subsequent sessions, indicating that students achieved competency early in the training.
Sonography	Höhne E, Recker F, Brossart P, Schäfer VS; 2024	RCT; The study compared teledidactic ultrasound teaching with traditional face-to-face instruction for medical students learning abdominal, thoracic, and thyroid ultrasound. The intervention group participated in seven online modules using portable Butterfly iQ probes with lectures, guided scanning, cloud-based image uploads, and annotated tutor feedback. The control group attended equivalent in-person sessions with live demonstrations and supervised practice. Both groups followed the same curriculum and were assessed with OSAUS exams and the B-QUIET scale. Skills taught included probe handling, image acquisition, systematic scanning, anatomical identification, and measurement techniques.	Learning success measured by OSAUS scores and image quality ratings via B-QUIET.	No significant differences between groups in final OSAUS exam scores; teledidactic group performed significantly better in FAST and aorta modules in B-QUIET image ratings.
Surgical suturing	Feeley A, Feeley Iain, Healy Eibhlin, Sheehan E, Hehir DJ; 2022	RCT; Participants underwent remote suturing training via live stream with teleproctoring, while another group received conventional in-person training. Both groups were assessed on their suturing skills before and after the training in a clinical skills lab.	General performance was evaluated by means of a validated suturing Global Rating Scale.	Performance improved significantly for both the intervention and the control group. Both groups reported equally high levels of satisfaction and engagement.

### Interactive learning systems

This category includes interactive multimedia modules and case-based virtual patient learning formats with and without feedback.

Researchers in the included studies have used interactive learning systems like Moodle-based (Perth, WA) platforms and virtual patient modules to teach practical skills, including chronic wound care and patient examination. These systems incorporated multimedia content, case-based scenarios, peer interactions, expert feedback and quizzes to support learning and skill acquisition.

Studies reported high satisfaction, improved theoretical knowledge and practical skills, with participants appreciating the efficiency and accessibility of interactive systems compared to traditional methods (e.g. face-to-face lectures, seminars or classroom-based discussion sessions and live demonstrations).

Fields of application included basic practical medical skills, basic practical pediatric skills, basic suturing skills and physical examination and history taking. Table 9 provides an overview of the data.

**Table 9:** Interactive Learning Systems

Area of application	Author(s); year	Intervention	Outcome(s)	Results
Basic practical medical skills	Schlupeck M, Stubner B, Erfurt-Berge C; 2021	Quantitative, descriptive; Participants engaged in a case-based online learning module focused on chronic wound care, which included instructional videos and interactive case studies to enhance skill acquisition. The course covered techniques such as measuring the ankle-brachial pressure index and applying compression bandages.	Students’ self-perceived competence in practical skills, their performance in a multiple-choice test and overall satisfaction with the course.	Results indicated a high level of satisfaction with the course content and structure. The majority of students felt they learned more from the online course than from a traditional lecture. Students also stated that the content was helpful for their future work as doctors.
Basic practical medical skills	Sałacińska I, Trojnar P, Gebriné KE, Törő V, Sárváry A, Więch P; 2025	Quasi-experimental; The study compared traditional manikin-based high-fidelity simulation with virtual patient simulation (Body Interact) for medical education. The intervention group completed acute care scenarios using Body Interact, while the control group used the Susie S2000 manikin for the same scenario. Both groups underwent identical preparation, simulation, and debriefing sessions. Outcomes measured included clinical decision-making, teamwork, and student perceptions of simulation quality, assessed with standardized tools (SSCL, SDS, EPQ) and a questionnaire.	Effectiveness and perception of simulation assessed via standardized tools—SSCL (satisfaction and self-confidence), SDS (simulation design), EPQ (educational practices), and a custom questionnaire.	No significant difference in overall effectiveness or perception between virtual and manikin-based simulations. Women in the virtual group rated active learning higher. Students aged 24–33 and those in higher years rated satisfaction, confidence, and effectiveness more positively.
Basic practical pediatric skills	Lehmann R, Bosse HM, Simon A, Nikendei C, Huwendiek S; 2013	Mixed-methods, descriptive; Participants worked with virtual patients that were designed to prepare them for pediatric procedural skills, including lumbar puncture and bladder puncture. The study aimed to investigate how both students and tutors perceive a blended learning approach that utilizes virtual patients for skills laboratory training.	Students’ perceptions of their preparedness for the skills laboratory, the efficiency of training time and overall satisfaction with the blended learning approach.	Overall acceptance of the blended learning approach was high. Students reported feeling well prepared for the skills lab and both students and tutors felt that the virtual patients helped to make more efficient use of training time by reducing the need for in-lab instruction.
Basic suturing skills	Rojas D, Cheung JJH, Weber B, Kapralos B, Carnahan H, Bägli DJ, Dubrowski A; 2012	RCT; The study examines the effect of expert facilitation versus independent peer learning on suturing and knot-tying skills within an online educational networking environment. Students uploaded videos of their performances to a Moodle-based platform, receiving either expert feedback or peer interactions without expert input.	Number of log-ins, comments posted and feedback provided by the expert. The study also included an exit survey where students reported on their perceived learning experience and skill retention.	No significant differences between the expert-facilitated group and the independent group in terms of the number of log-ins or comments posted has been found. The exit survey indicated that students in both groups felt they could learn the skills independently and there were no differences in perceived retention of skills.
Cardiopulmonary resuscitation	Creutzfeldt J, Hedman L, Heinrichs L, Youngblood P, Felländer-Tsai L; 2013	RCT; The study investigated whether multiplayer online 3D virtual world team training could improve medical students’ communication and teamwork in acute care scenarios. The intervention group practiced repeatedly in avatar-based simulations, while the control group received no additional training. Both groups were assessed before and after training using high-fidelity physical simulations. The virtual world platform enabled real-time team interactions under pressure, targeting structured communication and teamwork in acute care.	Primary outcome was improvement in team communication performance measured during full-scale simulations; secondary outcomes included participants’ perceived realism and usefulness of the training.	The intervention group showed significant improvement in team communication scores compared to the control group, while the control group’s scores did not change significantly.
Physical examination and history taking	Seifert L, Manap A, Sterz J, Gerlach F, Sader R; 2020	RCT; A Moodle-based virtual patient learning format with interactive case-based scenarios, multimedia content and feedback was compared to a peer-assisted learning format using case-based seminars with patient actors. Both groups completed pre-tests, attended separate training sessions and participated in post-tests and OSCEs to evaluate medical history taking, physical examination and patient feedback skills.	Short-term theoretical knowledge, long-term theoretical knowledge and practical skills assessed through OSCE performance. Also, student satisfaction and perceived learning effectiveness were measured.	Both groups showed significant improvement in theoretical knowledge, with no significant difference in OSCE performances between the groups. Participants in the intervention group performed slightly better on short-term tests for certain topics. Both groups rated their learning experiences positively.

### Robotic simulators

This category includes robotic surgery simulators (e.g. da Vinci Skills (Sunnyvale, CA) Simulator).

Researchers in the included studies have used robotic simulators like the da Vinci Skills Simulator and Mimic dV-Trainer (Göteborg, VG) to teach robotic surgical skills and cannulation. Training involved structured tasks focused on improving hand-eye coordination, dexterity and motion efficiency, with some studies comparing expert feedback, video-based guidance and independent practice.

Studies generally showed significant improvements in performance metrics, with repeated practice leading to proficiency, though expert feedback and guided training sometimes result in faster learning curves and better skill retention.

Fields of application included basic robotic-assisted surgery skills, bimanual carrying and peg transfer, robotic-assisted laparoscopy, robotic-assisted suturing and robotic-assisted vesicourethral anastomosis. Table 10 is summarizing the main characteristics.

**Table 10:** Robotic Simulators

Area of application	Author(s); year	Intervention	Outcome(s)	Results
Basic robotic-assisted surgery skills	Jiang L, Chen G, Li L, Chen Z, Yang K, Wang X; 2023	RCT; The study evaluated the feasibility and effectiveness of remote robotic surgery skills training using the ReTeRoS system, which integrates multi-channel video, real-time voice communication, and annotated feedback. Medical trainees in the intervention group practiced robotic simulator exercises remotely, while the control group received traditional in-person guidance. Both groups completed the same simulator tasks (“Peg Board 2,” “Match Board 2,” “Thread the Rings 1”) after a warm-up exercise. The program focused on two-handed coordination, suturing, instrument handling, motion efficiency, and collision reduction, showing the potential of remote robotic training.	Primary outcome: total simulator scores in the final skills tests. Secondary outcomes: sub-scores for seven performance metrics on the simulator and trainer fatigue load measured with NASA-TLX.	The remote group achieved significantly higher scores in “Match Board 2” and “Thread the Rings 1” than the control group and showed better economy of motion and fewer instrument collisions in certain tasks. Trainer fatigue load scores were lower in the remote group.
Basic robotic-assisted surgery skills	Kim JS, Jonas N, Rizvi TZ, Lin Z, Plewa D, Ricard C, Cheah YL, Simon CJ, Wright V; 2023	Quasi-experimental; The study evaluated whether completing a standardized VR robotic surgery curriculum improved medical trainees’ robotic suturing performance. Participants first performed a baseline 10-minute knot-tying task, then completed a 23-exercise VR curriculum on the Da Vinci Skills Simulator until reaching ≥ 90% proficiency, followed by a repeat knot-tying task. Performance was assessed using GEARS and modified OSATS scores, as well as knot quantity and efficiency. Skills targeted included robotic suturing, knot-tying, needle handling, and efficient console operation.	Changes in GEARS and modified OSATS scores, number of knots tied, and time to complete first knot.	Significant improvement in mean GEARS score (+ 2.43 points, *p* < 0.01), median modified OSATS score (+ 3.0 points, *p* < 0.01), average of three additional knots in 10 min (*p* < 0.01), and 114-second faster completion of first knot (*p* < 0.01).
Basic robotic-assisted surgery skills	Moglia A, Ferrari V, Morelli L, Melfi F, Ferrari M, Mosca F, Cuschieri A; 2014	Quantitative, descriptive; The study assessed medical students’ innate surgical abilities using the da Vinci Skills Simulator, a virtual reality system for practicing psychomotor and manipulative skills. Participants completed 26 exercises focusing on hand-eye coordination, bimanual dexterity and precise manipulation, with expert robotic surgeons performing the same tasks to validate the assessment metrics.	Overall scores for performance, completion times, economy of motion and applied force during the exercises.	Eight students significantly outperformed the others, achieving scores comparable to the expert surgeons. In contrast, 14 students performed significantly worse. No significant correlation was found between video game experience and performance on the simulator.
Basic robotic-assisted surgery skills	Vargas MV, Moawad G, Denny K, Happ L, Misa NY, Margulies S, Opoku-Anane J, Khalil EA, Marfori C; 2017	RCT; Participants trained on the da Vinci Skills Simulator, completing structured robotic suturing tasks until they reached proficiency or completed a maximum of ten attempts per task. Their performance was assessed through a final robotic suturing task on a live porcine model, with expert surgeons evaluating their skills based on video recordings.	Overall performance scores assessing surgical skill in multiple domains and the total task completion time for the suturing procedure on the live model.	No statistically significant difference in overall scores or task completion time between the intervention and control groups during the final suturing task on the porcine model was found.
Bimanual carrying and peg transfer	Chien JH, Suh IH, Park SH, Mukherjee M, Oleynikov D, Siu Ka-Chun; 2013	Quasi-experimental; The study assessed the effectiveness of a virtual reality (VR) simulator for training bimanual carrying and peg transfer skills, where participants used VR to learn and practice these tasks. Learning effects were measured pre- and post-training using the daVinci Surgical System to evaluate the participants’ skill improvement.	Kinematics of the surgical robotic arms were compared. These comprised time to task completion, total distance traveled and differences in both variables between pretraining and post training.	Participants in the intervention group scored significantly better than participants in the control group in both bimanual carrying and peg transfer tasks.
Robotic-assisted laparoscopy	Bric J, Connolly M, Kastenmeier A, Goldblatt M, Gould JC; 2014	Quasi-experimental; The study evaluated a robotic surgical training curriculum using the daVinci Surgical System and the daVinci Skills Simulator. Participants performed laparoscopic surgery tasks on the daVinci Surgical System, practiced on the VR simulator and then completed the tasks again on the system to assess their skills.	Task performance for the fundamental laparoscopic surgery tasks were measured (seconds to complete knot, penalty for a loose or slip knot, dropped pegs, seconds to transfer pegs back and forth).	There was a significant improvement in performance on the robotic fundamental laparoscopic surgery tasks after training with the daVinci VR simulator.
Robotic-assisted laparoscopy	Feifer A, Al-Ammari A, Kovac E, Delisle J, Carrier S, Anidjar M; 2011	RCT; Participants received training on either a hybrid or a virtual reality laparoscopic simulator, with assessments before and after training on the DaVinci console. Robotic surgical skills such as peg transfer, suturing, cutting and cannulation were tested.	Time, task specific accuracy, smoothness and pathfinding were assessed. In addition, a mean performance score was taken.	Statistically significant performance scores were found for all robotic tasks (within-group performance). Students who received no training showed no performance enhancement.
Robotic-assisted suturing	Kang SG, Ryu BJ, Yang KS, Ko YH, Cho S, Kang SH, Patel VR, Cheon J; 2015	Quasi-experimental; This study investigates the most effective repetitive training schedules for achieving proficiency in robotic suturing using a robotic virtual reality simulator. Participants were divided into groups with different training schedules and performed the “Tube 2” task on the Mimic dV-Trainer.	Time to completion and number of task repetitions required to reach a stable performance level.	Group 1 showed the most consistent improvement and the shortest time to task completion. Group 2 reached the performance plateau the fastest.
Robotic-assisted vesicourethral anastomosis	Kang SG, Yang KS, Ko YH, Kang SH, Park HS, Lee JG, Kim JJ, Cheon J; 2012	Quasi-experimental; The research examines how long and how many repetitions are necessary to achieve proficiency in performing a vesicourethral anastomosis with a robotic virtual reality simulator. Participants repeated the “Tube 2” task on the dV-Trainer, which simulates the da Vinci Surgical System, to observe when the learning curve plateaued.	Task time, economy of motion, master workspace range, instrument collisions, critical errors and overall performance score. The primary focus was on the number of repetitions required to reach the learning curve plateau.	The number of repetitions required to reach proficiency was 74, with an average task completion time of 138 s at the plateau. Every parameter measured improved between initial and final session.
Robotic-assisted vesicourethral anastomosis	Shim JS, Kim JY, Pyun JH, Cho S, Oh MM, Kang SH, Lee JG, Kim JJ, Cheon J, Kang SG; 2018	RCT; Participants were divided into three groups: one received expert proctoring with real-time feedback, another viewed an educational video with re-watch options and the third trained independently without guidance. After a mini-lecture and warm-up, all groups performed a complex robotic virtual reality task multiple times.	Time taken to reach proficiency, task completion time, economy of motion and errors. The learning curve was evaluated using a cumulative sum chart.	Groups 1 and 2 required 45 and 42 repetitions, respectively, to reach proficiency, compared to 37 repetitions in Group 3. However, group 3 took significantly longer to complete tasks post-proficiency, indicating inferior skill acquisition compared to groups 1 and 2.

### Blended/hybrid formats

This category includes combinations of online and hands-on simulation (e.g. skills labs, OSCE formats) as well as combinations of e-modules and in-person practice.

Researchers in the included studies have used blended and hybrid learning formats to teach various practical skills, combining online modules, instructional videos, case-based learning and hands-on practice. Commonly studied areas included anesthesia application and point-of-care assessments.

Studies generally showed that blended learning approaches improve knowledge acquisition, practical skills and self-confidence more effectively than conventional teaching methods such as instructor-led procedural training, supervised bedside teaching and non-digital hands-on skills lab training, with many participants reporting higher satisfaction and better preparation for practical examinations.

Fields of application included anesthesia application, basic practical anesthesiology skills, basic practical medical skills, basic practical nursing skills, basic practical pediatric skills, basic surgical skills, basic suturing skills, cardiorespiratory assessment, cardiac life support, endoscopy, intramuscular injections and nasal swabs, laparoscopy, neonatal resuscitation, newborn physical examination, pediatric suprapubic aspiration, physical examination and history taking, post-mortem examination, respiratory assessment and sonography. Table 11 is presenting an overview.

**Table 11:** Blended/Hybrid Formats

Area of application	Author(s); year	Intervention	Outcome(s)	Results
Airway clearance techniques	Alter IL, Overdevest J, Born H, Liao D, Michalowski A, Au V, Lin A, Baer NK, DiLisio C, Regenbogen E; 2024	Quasi-experimental; The study assessed a flipped classroom-based simulation workshop for teaching airway management to medical students. Participants watched 71 min of faculty-selected YouTube videos before a three-hour hands-on simulation session, which included two skills stations and a complex clinical scenario using high-fidelity manikins. Skills taught included bag valve mask ventilation, laryngoscopy, intubation, cricothyroidotomy, tracheotomy, tracheostomy care, as well as teamwork, delegation, and communication.	The outcomes included changes in students’ self-reported knowledge and confidence regarding the six airway management skills, as well as attitudes toward the workshop and the flipped classroom format.	Students reported statistically significant increases in knowledge and confidence for all skills except knowledge of tracheotomy, which showed improvement but did not reach significance. The average knowledge rating increased from 2.9 to 5.8 and confidence from 1.1 to 5.7, both with *p* < 0.001. Participants also reported high satisfaction with the workshop and the pre-learning materials.
Airway clearance techniques	Bellini R, Rossettini G, Letafatkar A, Dell’Isola A, Battista S; 2024	Case control; The study investigated physiotherapy students’ performance and satisfaction in a hybrid course on airway clearance techniques during COVID-19. The remote group joined via videoconference with digital slides and Moodle testing, while the control group attended in person with live demonstrations. All students were assessed through a knowledge test, a practical skills checklist, and a satisfaction questionnaire, focusing on techniques using devices such as PEP masks, Acapella, and Ez-PAP.	Primary outcomes were scores on a theoretical multiple-choice quiz (0–20), a practical skills checklist (0–10), and a combined total score (0–30). The secondary outcome was student satisfaction with the hybrid format.	Face-to-face students outperformed remote students in the total score (median difference = 6 points), and especially in practical skills (difference = 4 points). There was no clear difference in theoretical knowledge. Overall satisfaction was high (80%), with slightly more dissatisfaction among remote students.
Anesthesia application	Bock A, Kniha K, Goloborodko E, Lemos M, Rittich AB, Möhlhenrich SC, Rafai N, Hölzle F, Modabber A; 2021	RCT; The study compared the effectiveness of face-to-face, blended and e-learning methods for teaching the application of local anesthesia. Participants were assigned to an e-learning group with video demonstrations and reading materials, a blended learning group that combined e-learning with lectures or a conventional lecture-only group.	A knowledge test to assess knowledge gain, a practical skill small-group seminar and students’ attitudes towards the learning methods.	The blended group showed the best results in theoretical knowledge gain. No differences were found between groups on the hands-on assessment level.
Basic life support	Lehmann R, Thiessen C, Frick B, Bosse HM, Nikendei C, Hoffmann GF, Tönshoff B, Huwendiek S; 2015	RCT; The study evaluated the impact of a blended learning approach combining web-based virtual patient cases with standard manikin-based PBLS training. The intervention group worked through two interactive VP cases with videos and graphics in addition to paper handouts, while the control group prepared only with the handouts. Both groups then participated in standardized simulation-based PBLS training with structured feedback. Outcomes measured included procedural knowledge, adherence to algorithms and time requirements, procedural quality, and accuracy of self-assessment.	Procedural knowledge, adherence to the algorithm, adherence to temporal demands, procedural quality, and self-assessment.	The intervention group had significantly better procedural knowledge, algorithm adherence, temporal performance, and procedural quality after preparation and maintained advantages in some aspects after training; self-assessment was higher after preparation but similar after training.
Basic practical anesthesiology skills	Wulf H, Plöger B, Bepler S, Gockel A, Schmitt M, Kill C; 2014	Quantitative, descriptive; Trainees participated in a blended learning anesthesiology course that integrated lecture-based instruction, web-based self-study, simulator training and bedside teaching. The web-based component included interactive materials such as animations, videos and quizzes, allowing students to prepare independently before practical sessions.	Student satisfaction, course rankings and feedback scores.	The course consistently ranked among the top clinical courses, with students rating it highly on its structure and effectiveness in teaching practical skills.
Basic pratical medical skills	Agostino S, Cherasco GM, Papotti G, Milan A, Abate Daga F, Abate Daga M, Veglio F; 2025	Quasi-experimental; The study evaluated the impact of a curricular internship combining flipped classroom and simulation-based learning on medical students’ perceived clinical competence. Students first watched online preparatory videos, then participated in morning skill stations covering history taking, vital signs, and physical examinations, followed by afternoon high-fidelity simulation scenarios on common clinical conditions. Training emphasized physical exam techniques, recognition of clinical signs, and teamwork/communication in clinical practice.	Change in self-perceived clinical competence across 17 items, measured with a Likert-scale survey administered before and after the intervention.	There were statistically significant improvements (all *p* < 0.001) in self-perceived clinical skills across all domains. The greatest improvements were observed among third-year students and those who had not yet passed the Clinical Methodology course.
Basic practical medical skills	Ayandeh A, Zhang XC, Diamond JF, Michael SH, Rougas S, 2020	Quantitative, descriptive; A 10-week extracurricular procedural skills course for pre-clerkship medical students was developed using a hands-on, flipped classroom model with instructional videos to teach skills such as basic suturing, lumbar puncture and endotracheal intubation. The course provided practical training beyond the standard curriculum, emphasizing active practice and skill retention.	Knowledge and skill acquisition were assessed using a multiple-choice exam and a practical exam. Online surveys were used to assess subjective confidence of the students.	Students who took part in the course reported increased levels of self-reported confidence and performed better regarding the practical exam and multiple-choice exam than students who only participated in the standard curriculum.
Basic practical medical skills	Chan E, Botelho MG, Wong GTC; 2021	Quantitative, descriptive; The study assessed the effectiveness of a flipped classroom format for acquiring clinical skills, where participants watched a demonstration video before class and practiced as triads. Students recorded their performances of bag mask ventilation and intravenous cannulation skills during the self-directed practice session.	A questionnaire on knowledge of bag mask ventilation and intravenous cannulation, two blinded assessors evaluated the self-recorded performances.	Participants who watched the demonstration videos scored higher on skill performance than participants who indicated they did not watch the videos beforehand. No significant differences could be found on intravenous cannulation. Questionnaire results showed that most participants preferred the new approach of clinical skill teaching and rated it as useful for the acquisition of bag mask ventilation and intravenous cannulation skills.
Basic practical medical skills	Enoch LC, Abraham RM, Singaram VS; 2024	Mixed-methods, descriptive; The study explored students’ and tutors’ perceptions of transferring online pre-clinical skills training to hands-on practice and proposed a blended teaching model. Students participated in an adapted online curriculum with recorded lectures, quizzes, Zoom sessions, simulations, and role-play, with some also joining an optional onsite “Readiness Programme” for practicing procedural skills on simulated patients. Assessments included an online OSCE for cognitive and affective domains and observed performance during the readiness programme. The program targeted history-taking, physical and obstetric examination, Pap smears, and procedural competencies in early medical education.	Outcomes included students’ and tutors’ perceptions of the effectiveness of the adapted teaching model in transferring and retaining clinical skills, student preparedness for skills laboratory practice, and the acceptability of a blended learning approach.	Results showed high acceptability (86% positive) for using online platforms in clinical skills training. Students and tutors reported that online simulations with targeted onsite practice effectively prepared learners. Five themes emerged: asynchronous online materials aided preparation; synchronous online teaching improved comprehension; synchronous onsite learning consolidated skills; asynchronous onsite practice reinforced skills; and continuous assessment and feedback enhanced engagement. Tutors initially doubted skill transfer from online to hands-on settings but were positively surprised by students’ performance in the RP.
Basic practical medical skills	Fu XT, Hu Y, Yan BC, Jiao YG, Zheng SJ, Wang YG, Zhang JY, Wang ZB; 2022	RCT; The blended teaching format used a Massive Open Online Course, problem-based learning, case-based learning and supplementary tools such as video conferences and messaging groups. The course focused on developing skills in neurological examination and lumbar puncture.	General performance scores for both theoretical and practical skill performances.	The intervention group outperformed the control group on both theoretical and practical skills.
Basic practical medical skills	Heitmann H, Fischer E, Wagner P, Pötter D, Gartmeier M, Schmidt-Graf F; 2023	Quasi-experimental; A flipped classroom online preparation learning format was evaluated for teaching neurological exam knowledge and practical skills, including examination techniques, information transfer and documentation. The format included a script, video and screencasts on neurological examination and background information, followed by bedside courses.	Self-reported ratings of knowledge and skills on numerical rating scales. Additionally, time needed to recapitulate theoretical contents during the in-person teaching session was assessed.	Theoretical knowledge was rated significantly higher in the intervention group compared to the control group. Practical skills of students that received the flipped classroom approach were rated significantly higher compared to the control group. Students in the intervention group needed less time to recapitulate theoretical contents.
Basic practical medical skills	He Z, Li H, Lu L, Wang Q, Wu Q, Lu L; 2024	RCT; The study evaluated the effectiveness of blended teaching in an undergraduate clinical skills training course compared with traditional offline teaching. The blended group completed 25% of class hours online via the University Open Online Courses platform (lectures, demonstration videos, PPTs, exercises, tests) and 75% offline with practical sessions, group evaluations, and feedback. The control group received the same distribution of theoretical and practical teaching but delivered entirely offline. Both groups followed the same 4-month syllabus and were assessed through an OSCE covering CPR, physical examination, puncture procedures, disinfection & draping, gowning & gloving, and incision & suturing.	Primary outcome: OSCE scores at the end of the course. Secondary outcome: student feedback on blended teaching via questionnaire.	The intervention group scored significantly higher than the control group in total OSCE score and all individual skill stations (*p* < 0.05). The majority rated blended teaching positively, considered it better than traditional offline teaching, and expressed willingness to apply it in other courses. Emergency medicine, internal medicine, and surgery were the most popular modules.
Basic practical medical skills	Meshram K, Srivastava T, Meshram A, Meshram H, Singh A, Mishra P; 2023	Quasi-experimental; The study compared a seven-pronged blended learning approach with traditional teaching for Indian medical undergraduates. The blended model integrated virtual classrooms, online demonstrations, webinars, mannequin-based simulations, animated videos, face-to-face training, query resolution, and mentoring, while the control group received standard lectures and demonstrations. Both groups attended scheduled lectures and practical sessions before being assessed through theory (MCQ, OSVV) and practical (OSPE) exams. The program emphasized psychomotor, communication, and attitude skills in addition to theoretical knowledge.	Theory exam scores, viva performance, and practical exam scores.	Intervention group had significantly higher theory scores (MCQ) in both phases; no significant difference in practical scores between groups; viva scores were significantly higher only in the second phase.
Basic practical nursing skills	Cao W, Hu L, Li X, Li X, Chen C, Zhang Q, Cao S; 2021	RCT; The study evaluated the effectiveness of MOOC-based blended learning versus traditional classroom teaching in the Fundamental Nursing II course for undergraduate nursing students. The blended group accessed online video lectures, surveys, and assignments via a university MOOC platform, supplemented with in-class discussions and case-based activities. The control group received conventional lecture-based teaching with clinical cases. Both groups followed the same curriculum with identical objectives and assessments, covering injection techniques, disease observation, pain and hospice care, illness assessment, nursing operations, health guidance, and communication skills.	Primary outcome was test scores (daily performance, operational performance, theoretical achievements, final grades); secondary outcomes were changes in critical thinking abilities and student feedback.	MOOC-based blended group had higher operational performance, theoretical achievements, and final grades, but lower daily performance. Most critical thinking ability measures improved more in the MOOC group except for open-mindedness and systemic capacity, which improved more in the control group. Student satisfaction and perceived learning effects were higher in the MOOC group.
Basic practical nursing skills	Joseph MA, Natarajan J, Labrague L, Al Omari O; 2025	Quasi-experimental; The study examined the effects of a flipped classroom model on undergraduate nursing students in a Fundamentals of Nursing course. The intervention group accessed pre-class materials, including instructional videos, infographics, and quizzes, then engaged in case discussions and hands-on practice during lab sessions. The control group received traditional demonstration-based instruction followed by practice. Both groups completed knowledge quizzes, skill performance assessments, and surveys. Outcomes included procedural knowledge retention, skill performance, self-directed and collaborative learning, critical thinking, work engagement, learning ability, and satisfaction.	Procedural knowledge retention (pre-quiz/post-quiz scores), skill performance, self-directed learning, collaborative learning, critical thinking, work engagement, learning ability, and satisfaction.	Flipped classroom group had significantly higher pre-quiz scores but no significant differences in post-quiz, skill performance, self-directed learning, collaborative learning, critical thinking, or work engagement compared to the control group. Learning ability and satisfaction were significantly lower in the flipped classroom group.
Basic practical nursing skills	Kim SY, Kim SS, Yu M; 2023	RCT; The study examined the impact of a flipped learning approach combined with high-fidelity simulation on nursing students’ clinical judgment, self-confidence, and satisfaction. The intervention group prepared with online pre-class video lectures before participating in obstetric nursing simulation scenarios, while the control group received the same simulation training without flipped learning. Both groups used high-fidelity mannequins and completed pre- and post-tests. Outcomes measured included clinical judgment, self-confidence, and learning satisfaction.	The outcomes measured were clinical judgment, self-confidence, and learning satisfaction.	The results showed that the experimental group had significantly higher clinical judgment and self-confidence scores compared to the control group. Satisfaction with the learning method was also higher in the experimental group.
Basic practical nursing skills	Li YR, Zhang ZH, Li W, Wang P, Li SW, Su D, Zhang T; 2023	Quasi-experimental; The study evaluated the effectiveness of a blended learning program in a surgical nursing skills course compared to traditional face-to-face teaching. The blended group used an LMS with pre-class videos, quizzes, online discussions, and case scenarios, combined with classroom sessions and assignments, while the control group received only in-person teaching. Both groups studied the same content and took identical final exams. The training emphasized surgical nursing procedures such as closed chest drainage, with a focus on theoretical understanding, standardized procedural steps, and application in clinical case scenarios.	Skill performance scores and learning engagement levels (measured via UWES-S scale); qualitative insights on student attitudes, experiences, and suggestions.	Blended learning group achieved higher skill performance (81.19 ± 5.86 vs. 76.29 ± 8.06) and higher learning engagement scores (79.67 ± 14.99 vs. 48.73 ± 18.18) than the traditional group; qualitative data highlighted perceived flexibility, improved understanding, and convenience, alongside concerns about technological requirements and self-discipline.
Basic practical nursing skills	Meng XH, Xu XY, Chen HL, Zhang L; 2022	RCT; The study evaluated the effectiveness of combining e-learning, peer teaching and flipped classroom methods for a physiology lab course. Participants completed e-learning modules (PowerPoint slides, recorded lectures, procedural videos, virtual experiments), engaged in peer teaching and practiced procedural skills, compared to a control group with traditional instruction.	Students’ knowledge and proficiency scores regarding procedural skills.	The intervention group significantly outperformed the control group across knowledge and proficiency scores.
Basic practical nursing skills	Smith TS, Holland AC, White T, Combs B, Watts P, Moss J; 2021	Quasi-experimental; Instructional videos on suturing and abscess drainage were reviewed by participants, who practiced using mailed simulation kits and engaged in virtual sessions with live demonstrations, feedback and skill validation from facilitators. The educational model combined prerecorded videos with interactive virtual training.	Participant’s satisfaction, self-confidence and competency validation.	Participants expressed high satisfaction with the learning format and materials. All participants who completed the validation phase met competency for both skills.
Basic practical nursing skills	Yeom GJ, Yang J, Kim J, Kim HS; 2021	Quasi-experimental; Participants engaged in a blended learning approach that combined online materials, hands-on simulation training, peer collaboration and self-reflection exercises to develop ventilator nursing skills. The control group followed a self-directed learning approach with only online materials, without simulation-based instruction or peer interaction.	Knowledge of mechanical ventilation, self-assessed self-confidence and ventilator nursing skills performance.	The intervention group showed significantly higher scores in knowledge, self-confidence and ventilator nursing skills performance compared to the control group. Students in the intervention group particularly valued hands-on practice and instructional videos.
Basic practical pediatric skills	Uther P, Van Munster KA, Briggs N, O’Neill S, Kennedy S; 2019	Quasi-experimental; Participants in the flipped-simulation group completed an online module with tutorials and videos before attending a three-hour simulation session focused on pediatric clinical skills. The alternative group participated in a play-based session interacting with a preschool child and parent in a supervised setting.	Knowledge scores from pre- and post-tests, procedural knowledge retention scores in the follow-up quiz and self-reported confidence and satisfaction scores from a post-course survey.	The intervention group showed significant improvements in both short-term and long-term knowledge retention compared to the play-based group. The intervention group demonstrated a higher mean score on the post-test and maintained better knowledge retention in the follow-up assessment. They also reported high confidence in clinical skills.
Basic surgical skills	Hecker A, Nischwitz SP, Petritsch J, Holzer-Geissler JCJ, Draschl A, Wegscheider T, Lumenta DB; 2023	Quasi-experimental; This study compared a hybrid online surgical skills training format with a traditional in-person session for teaching medical students a mandatory single interrupted suture (SIS) class during the COVID-19 pandemic. The hybrid group watched a 4-minute instructional video and participated in a 20-minute one-on-one tutoring session with a trained student tutor before completing an OSCE. The control group received a 45-minute in-person class led by surgical residents or attendings with demonstrations and supervised practice. Both groups completed the same knowledge and skill assessments. The intervention combined digital video instruction and individualized peer tutoring, focusing on surgical handling and SIS performance.	Self-assessed theoretical and practical knowledge of SIS before and after the class.	Both formats significantly improved self-assessed theoretical and practical knowledge. Pre-class ratings were higher in the hybrid group, but post-class differences between groups were not statistically significant. In-person training showed larger effect sizes.
Basic surgical skills	Kurz S, Buggenhagen H, Wachter N, Penzkofer L, Dietz SO, König TT, Heinemann MK, Neulen A, Hanke LI, Huber T; 2022	Quasi-experimental; This study evaluated the feasibility and effectiveness of a hybrid OSCE format for teaching surgical skills. Participants underwent a surgical block course followed by a hybrid OSCE, where they demonstrated practical surgical skills in a physical room while being observed remotely via videoconferencing.	Overall performance in the hybrid OSCE and students’ feedback on their experience with the format.	92% of the students rated the hybrid OSCE format positively. Skill acquisition was rated highly.
Basic surgical skills	Liebert CA, Mazer L, Bereknyei Merrell S, Lin DT, Lau JN; 2016	Mixed-methods, descriptive; The study explored medical students’ perceptions of a simulation-based flipped classroom in the surgery clerkship and identified best practices for its use. Students prepared with online videos on Open edX before sessions, then engaged in case-based reasoning, simulation training, and the SICKO surgical decision-making game during weekly 4-hour sessions. Skills covered included suturing, knot tying, chest tube placement, central line insertion, endoscopy, laparoscopic skills, trauma team simulations, and surgical decision-making.	Students’ ratings of the overall curriculum, specific components, and simulation sessions; perceived benefits; ease of use of platforms; and suggested improvements.	90% rated the overall curriculum as excellent or outstanding; simulation-based skill sessions were most favored; Khan Academy-style videos rated highest for effectiveness; most students wanted the curriculum continued (95%) and applied to other clerkships (84%).
Basic surgical skills	Lim KT, Koh BY; 2022	Mixed-methods, descriptive; This study evaluated a basic surgical skills workshop combining digital and in-person training. Participants completed online modules, watched videos, answered questions and participated in a hands-on workshop with live demonstrations and supervised practice in skills like knot tying, suturing and laparoscopic procedures.	Knowledge acquisition, practical surgical skills scores and attitudes related to safe surgical practices.	The workshop was evaluated positively by participants and all students passed the post-workshop test and achieved satisfactory grades in the practical skills assessment.
Basic surgical skills	Shen AH, Alfonso AR, Cuccolo NG, Johnson AR, Lee BT, Lin SJ; 2022	Mixed-methods, descriptive; A 4-week virtual curriculum based on a flipped classroom model was implemented, incorporating case discussions, video-assisted surgical workshops and peer-to-peer interactions. Participants engaged in structured learning activities designed to enhance their knowledge of plastic and reconstructive surgery.	Self-reported confidence in knowledge of anatomy, surgical approaches, wound care, suturing and knot-tying techniques as well as community connectedness and preparedness for plastic surgery subinternships.	Significant improvements in confidence were found across all knowledge and skill areas, especially in discussing anatomy, surgical approaches and specific topics within plastic surgery. Those who attended the workshops felt more competent in suturing and knot tying and applicants felt better prepared for subinternships and connected to the community.
Basic suturing skills	Rieger UM, Pierer K, Farhadi J, Lehmann T, Röers B, Pierer G; 2009	Mixed-methods, descriptive; Students in the blended learning group used multimedia CD-ROMs with video demonstrations and interactive materials for self-directed learning before attending practical suturing sessions. Both the blended and traditional groups participated in hands-on training and were assessed through an OSCE on basic surgical suturing techniques.	Student satisfaction, skill acquisition and subjective feedback on utility of the blended learning approach. OSCE results were used to compare the success rates of students.	The intervention group showed significantly higher evaluations in terms of teaching materials, subjective success and course preparation compared to the traditional format. Additionally, there was a 10% improvement in the pass rate for OSCE exams in the intervention group.
Bedside cardiac assessment	Meisel JL, Chen DCR, Cohen GM, Bernard SA, Carmona H, Petrusa ER, Opole IO, Navedo D, Valtchinov VI, Nahas AH, Eiduson CM, Papps N; 2023	Quasi-experimental; The study developed and evaluated a flipped-classroom curriculum for teaching bedside cardiac assessment (BCA) to medical clerkship students. The intervention group completed 1.5–2 h of pre-class assignments, including short videos and cognitive exercises, followed by two interactive sessions with case discussions, jugular venous pressure exercises, heart sound identification games, and simulated patient encounters emphasizing urgent communication and trust-building. The control group received standard clerkship BCA instruction. Practical skills taught included systematic BCA (history-taking, JVP measurement, heart sound recognition), diagnostic reasoning, linking pathophysiology to findings, and patient-centered communication.	Outcomes measured were changes in self-reported confidence, BCA knowledge scores, thematic feedback on curriculum design, and perceived professional identity formation.	Results showed significantly higher proportions of positive confidence changes in key BCA abilities in the intervention group; knowledge scores improved similarly in both groups. Thematic analysis indicated meaningful engagement, effective instructional design, and evidence of skill transfer (e.g., more frequent JVP assessments).
Cardiorespiratory assessment	Balaraman T, Sliwinski Z; 2021	Quasi-experimental; The feasibility of a case-based combined online video and written assignment was examined for teaching cardiorespiratory assessment skills. The approach integrated video demonstrations and written tasks to enhance practical skill development in physiotherapy.	Physiotherapy assessment for cardiorespiratory conditions was assessed by means of two standardized OSCE-performances.	Students’ performance in the second OSCEs improved after carrying out the combined online video and written assignment.
Cardiopulmonary Life Support	Beom JH, Kim JH, Chung HS, Kim SM, Ko DR, Cho J;2018	RCT; The study investigated the impact of a flipped-classroom approach on teaching ACLS to senior medical students. The intervention group received narrated PowerPoint slides before class and analyzed an ACLS scenario video in small groups during class, followed by simulation training. The control group had a traditional lecture before the same simulation training. Both groups underwent two hours of ACLS simulation with performance assessments in standardized scenarios. Skills assessed included defibrillation, compressions, airway management, drug use, and identifying cardiac arrest causes.	Primary outcome was performance in a simulation-based ACLS assessment. Secondary outcome was student satisfaction with the class format.	Students in the flipped classroom had slightly higher ACLS simulation scores (70.9 vs. 67.1) and slightly higher satisfaction scores, but neither difference was statistically significant. Some individual checklist items showed better performance in the flipped group.
Cardiopulmonary resuscitation	Liu H, Huang H, Li M, Mao P, Zhang A, Sun Y, Liu Z, Tao H, Zhao S, Xia Y, Zhou J, Liu J; 2025	Quasi-experimental; The study evaluated a three-step “online–simulation–bedside” teaching method for emergency and critical care nursing interns using the Kirkpatrick model. The intervention included an online CPR theory module, followed by high-fidelity simulation training with CPR scenarios, and finally a 30-day bedside clinical practice phase. Skills trained included CPR in a team setting, clinical reasoning, operational competence, and collaborative emergency response.	The outcomes measured were satisfaction (reaction), teamwork and clinical thinking (learning), confidence and active participation (behavior), and CPR skills test scores (result).	The results showed significant improvements in all outcomes. Satisfaction was very high (98.8%), teamwork and clinical thinking improved, confidence and participation increased, and all participants scored at least “good” in CPR skills assessment, with a mean score of 90.09.
Cardiac life support	Boysen-Osborn M, Anderson CL, Navarro R, Yanuck J, Strom S, McCoy CE, Youm J, Ypma-Wong MF, Langdorf MI; 2016	Quasi-experimental; The study compared flipped classroom/team-based learning with lecture-based training for teaching advanced cardiac life support (ACLS) skills. Participants in the flipped classroom group engaged in 10.5 h of team-based learning, 9 h of podcast learning and simulation training, while the lecture-based group had traditional lecture and simulation training.	Multiple-choice test on cardiac life support knowledge, cardiac rhythm test and clinical management test.	Written test scores on the multiple-choice test and the cardiac rhythm test were higher for the intervention group. More students of the lecture-based learning group failed 1 of the three tests compared to the intervention group.
Cardiopulmonary resuscitation	Ohlenburg, H, Arnemann PH, Hessler M, Görlich D, Zarbock A, Friederichs H; 2024	RCT; The study investigated whether a flipped classroom approach using the interactive e-learning tool iMuVi improves resuscitation training. One group completed iMuVi before two simulator-based ALS scenarios, while the other used iMuVi between the two scenarios. The tool provided learner-paced, multi-perspective audiovisual simulations of a resuscitation case. Outcomes measured included team performance (leadership, communication, task management) and CPR quality (compression depth and rate).	Primary: team performance using the TEAM score. Secondary: objective CPR metrics from the simulator (e.g., compression rate, fraction, and no-flow time).	Intervention teams performed significantly better in the first scenario than control teams (e.g., TEAM global rating 7.5 vs. 5.6; *p* < 0.01). These differences disappeared in the second scenario, when both groups had used iMuVi.
Cephalometric analysis	Karandish M, Karimian Z, Parastar M; 2024	Quasi-experimental; The study evaluated a flipped classroom blended learning model for improving dental students’ knowledge and skills in lateral cephalogram tracing. Students studied online materials in advance via the LMS and then engaged in short lectures, case discussions, Q&A, and quizzes during class, supported by forum-based homework. Their results were compared with those of a previous cohort taught through traditional lectures. Skills included landmark identification, interpretation of cephalometric lines and angles, and radiographic analysis, alongside student satisfaction and exam performance.	Outcomes included students’ attitudes toward e-learning, satisfaction with course quality, perceived effectiveness of blended learning tools, self-assessed knowledge and skills, and final exam scores.	Significant improvements in attitudes toward e-learning, self-assessed theoretical and practical skills (all *p* < 0.001), and higher final exam scores compared to the previous semester’s traditional group (*p* < 0.001).
Endoscopy	Yoshida Y, Uno T, Tanaka H, Hakosaki I, Shigeta K, Yano R; 2022	Qualitative, descriptive; Nursing students engaged in a blended learning program that combined face-to-face lectures, self-study audiovisual materials, independent practice in a skills lab and optional instructor-led feedback sessions before their skills test. The digital intervention included videos demonstrating correct and incorrect techniques, as well as silent demonstrations of the full procedure.	Students’ cognitive changes as they progressed through different phases of skill acquisition. Themes derived from interviews and video observations, such as their ability to self-evaluate and adjust techniques.	Six cognitive changes were identified: Feeling that the skill is easy to acquire, practicing without much thought, realizing the difficulty of translating thoughts into practice, experiencing a sense of purpose in each technique, gaining a perspective to evaluate one’s skills and developing one’s unique approach. Additionally, students’ metacognitive skills, including self-evaluation and independent adjustments, were crucial for skill development.
Intramuscular injections and nasal swabs	Bieri J, Tuor C, Nendaz M, Savoldelli GL, Blondon K, Schiffer E, Zamberg I; 2023	Mixed-methods, descriptive; The effectiveness and perceived satisfaction of a student-teacher-designed educational activity were assessed for teaching intramuscular injection and nasopharyngeal swab skills. The training followed a blended learning format, combining e-learning and video-based self-directed assignments with face-to-face small-group practice using simulators.	Students’ confidence in performing both skills, knowledge about both skills and satisfaction with the format.	Students’ confidence in performing these skills increased significantly. The same applied for knowledge acquisition. Students reported a high level of satisfaction with the learning format.
Intravenous cannulation	Carter H, Hanks S, Johnson R, Gale T; 2024	Quasi-experimental; The study investigated the impact of a blended intravenous cannulation course on final-year medical students’ preparedness for clinical practice. Students first undertook a high-fidelity simulation of a difficult cannulation case, then completed a four-module e-learning package (“Prepare, Procedure, Preserve, Communication & Human Factors”) with videos and quizzes, and finally performed a second simulation. The program targeted both technical skills (cannulation, sterility) and non-technical skills (communication, teamwork, adaptability).	Development of learner capability and preparedness for practice, increased confidence, improved integration of technical and non-technical skills, and enhanced patient-focused approaches.	Participants reported that after the e-learning and second simulation, they were better able to adapt to complex scenarios, integrate communication and teamwork into their technical practice, and felt more confident and prepared for real-world cannulation tasks. The simulations were identified as the most valuable component for skill development, with e-learning providing a structural framework.
Laparoscopy	Chiu HY, Kang YN, Wang WL, Huang HC, Wu CC, Hsu W, Tong YS, Wei PL; 2018	RCT; The study assessed the effectiveness of a simulation-based flipped classroom approach for teaching laparoscopic suturing and intracorporeal knot-tying skills, with participants engaging in video-based learning. The flipped classroom group was compared to a conventional classroom group and performance was evaluated by instructors after training.	An assessment checklist based on a modified Objective Structured Assessment of Technical Skills tool comprising, among others: place needle at 90° angle to tissue, drive needle with supination and pull suture through to establish short free end and additional surgery-related factors.	Students in the intervention group showed significant improvements in all categories of performance (e.g. number of stitches, stitch quality, safe needle removal).
Nasogastric tube insertion	Merrou S, Baslam A, Idrissi Jouicha A, Ouhaz Z, El Adib AR; 2023	Quasi-experimental; The study assessed the impact of blended training on nursing students’ ability to perform nasogastric tube insertion. The blended group viewed pre-recorded video capsules on Moodle before attending simulation sessions, while one control group had only theoretical lectures and the other had lectures plus simulation. Both the blended and simulation groups practiced nasogastric tube insertion under supervision. Skills emphasized included the procedural and psychomotor steps of correctly inserting a nasogastric tube.	Declarative knowledge (MCQ scores), procedural knowledge (practical performance scores), technique completion time, and student satisfaction.	Both simulation and blended groups outperformed the traditional group in declarative and procedural knowledge. The blended group achieved the best procedural scores and shortest completion times. Significant correlations were found between declarative and procedural knowledge, and procedural knowledge and completion time. Student satisfaction with simulation was high (> 70% very satisfied).
Neonatal resuscitation	Johnson C, Shen E, Winn K, Digiacobbe G, Akinola M; 2020	Quasi-experimental; The study developed and evaluated a blended learning approach for neonatal resuscitation. Participants completed a web-based module, watched a demonstration video and participated in simulation exercises.	Knowledge about neonatal resuscitation, neonatal resuscitation performance and perceptions of interprofessional skills.	Students showed significant improvements from pre- to posttest scores regarding knowledge and performance. Learners reported increased skills in communication, leadership and teamwork.
Neonatal resuscitation	Kudreviciene A, Nadisauskiene RJ, Tameliene R, Tamelis A, Nedzelskiene I, Dobozinskas P, Vaitkaitis D; 2019	Cohort study, single group; The study evaluated the retention of neonatal resuscitation skills among medical students after they used a hybrid learning system. The skills were assessed at baseline and again at 6 and 12 months, with training including distance learning, hands-on simulation and peer-to-peer teaching.	Retention of neonatal resuscitation skills.	Students showed a significantly smaller decrease in skill retention performance after using the hybrid learning system.
Newborn physical examination	Stewart A, Inglis G, Jardine L, Koorts P, Davies MW; 2013	RCT; Participants in the intervention group received traditional bedside teaching supplemented by an online learning module with multimedia components, including videos, clinical images and text. The control group followed the standard neonatology educational program with weekly lectures and bedside tutorials but did not have access to the online module.	Cumulative checklist scores for newborn examination, completion rates of essential items and participant satisfaction with the learning experience.	Participants in the intervention group scored significantly higher on the newborn examination than participants in the control group. However, there was no significant difference in the completion of essential checklist items or in overall learning satisfaction between the two groups.
Practical Otorhinolaryngology skills	Wehling J, Dombrowski T, Johannsen K, Volkenstein S, Dazert S, Weiss NM; 2024	Quasi-experimental; The study evaluated a flipped-classroom ENT teaching program supported by a SkillsLab to enhance practical skills training. Students in the intervention group prepared with a digital module on Moodle and then practiced in structured SkillsLab workstations, while the control group learned the same skills through conventional in-person teaching without a SkillsLab. Both groups practiced oral inspection, anterior rhinoscopy, otoscopy/microscopy, rigid and flexible endoscopy, audiometry, nasal packing, nasogastric tube insertion, and cricothyrotomy. The program measured effects on motivation and self-assessed competence in ENT education.	Both groups had high baseline motivation and competence after the digital module; a statistically significant increase in motivation and self-assessed competence occurred only in the SkillsLab group (*p* < 0.001).	The SkillsLab group started with lower self-assessment but surpassed the control group in subjective competence after the face-to-face day; motivation levels became similar, with the SkillsLab group rating SkillsLab-related items more positively.
Paramedic services	Kaplan A, Özdemir C, Kaplan Ö; 2023	RCT; The study examined the impact of a flipped classroom model on paramedic students’ acquisition of clinical skills. The intervention group received instructional videos before class and then engaged in interactive sessions and practical training, while the control group was taught via traditional lectures and demonstrations. Both groups practiced the same clinical skills on simulation models and were evaluated with standardized checklists, focusing on procedures such as IV catheterization, blood collection, blood pressure measurement, IM injection, and urinary catheterization.	Outcomes measured were checklist-based skill performance scores (primary), time spent on practice vs. theory (secondary), and subjective student evaluation of the FCM (tertiary).	The intervention group showed slightly higher skill scores and practiced longer, but differences were not statistically significant. However, students in the FCM group reported higher motivation, less anxiety, and better learning perception.
Paediatric emergency training	Thomson NM, Campbell DE, O’Leary FM	Quasi-experimental; The study developed and evaluated a blended learning program for teaching paediatric resuscitation to medical students. Participants first completed an e-learning module covering theoretical aspects of paediatric BLS, ALS, bag-mask ventilation, rhythm recognition, and defibrillation, then attended a 90-minute practical session using low-fidelity simulation with a “pause and discuss” format. Skills trained included paediatric BLS and ALS procedures, intraosseous access, safe defibrillation, rhythm recognition, and teamwork and communication in resuscitation scenarios.	Primary outcome: significant improvement in knowledge scores from pre-course to post e-learning and at 8-month follow-up; Secondary outcomes: self-reported increases in knowledge, confidence, and ability in BLS and ALS maintained at follow-up.	MCQ median score increased from 12/23 pre-course to 21/23 post e-learning, remaining at 17/23 after 8 months; significant self-rated improvements in knowledge, confidence, and ability in both BLS and ALS; majority rated simulation as most useful.
Pediatric suprapubic aspiration	Bosse HM, Martin A, Ling K, Memili S, Patalong S, Rings V, Jasper ED, Luczak K, Liesenjohann S, Witsch A, Wengel C; 2015	Quasi-experimental; The study assessed a blended-learning scenario that included virtual patients and a toddler simulation manikin for teaching suprapubic aspiration in a pediatric setting. Students prepared for the training by watching short video clips on the procedure, then practiced the aspiration while interacting with virtual patients or parents.	Students’ acceptance and subjective view on aspects of the manikin for learning effectiveness.	Ratings of learning success were high for the blended-learning simulation scenario.
Physical examination	Uchida S, Shikino K, Ishizuka K, Yamauchi Y, Yanagita Y, Yokokawa D, Tsukamoto T, Noda K, Uehara T, Ikusaka M; 2022	Mixed-methods, descriptive; Medical students in the flipped classroom group first watched a seven-minute e-learning video on deep tendon reflex concepts before engaging in a procedural teaching session. The control group attended a traditional lecture before receiving the same procedural instruction.	Self-confidence scores, pre- and post-procedural teaching and mastery scores for technical ability.	The intervention group showed significantly higher pre-test self-confidence compared to the control group. Post-test self-confidence and mastery scores were similar between the groups.
Physical examination and history taking	Turk B, Ertl S, Wong G, Wadowski PP, Löffler-Stastka H; 2019	Quasi-experimental; Medical students in the blended learning group engaged with case-based textbook resources, interactive e-learning modules and supervised simulated patient sessions to reinforce clinical skills. The comparison group consisted of a previous cohort that did not participate in the structured case-based blended learning program.	OSCE performance scores, which measured clinical skills, procedural knowledge and clinical reasoning.	A significant improvement in OSCE scores was found.
Post-mortem examination	Heide S, Lessig R, Diers V, Rönsch M, Stoevesandt D; 2016	Quantitative, descriptive; The evaluation focuses on a blended learning format that combines a skills lab station with an e-learning module to teach post-mortem examination skills. Participants underwent the new learning format at the beginning and end of the semester to assess their skill acquisition.	Students’ self-reported competence and confidence in performing post-mortem examinations.	Participants showed improved competence and confidence scores at the end of the semester.
Respiratory assessment	Choi JY, Lee SE, Bae J, Kang S, Choi S, Tate JD, Yang YL; 2021	Mixed-methods, descriptive; The study investigated the effectiveness of a flipped-classroom learning approach for acquiring knowledge and skills related to respiratory system assessment. Participants engaged in self-directed pre-class activities, such as online lecture videos and reading materials, followed by interactive team-based learning activities inside the classroom.	Perceptions of students regarding student-centeredness and the quality of the learning environment in terms of teaching, social and cognitive factors were examined.	Students’ perceptions of student-centeredness increased significantly. The quality of the learning environment in terms of teaching and social factors increased, too, although not significantly. FC was seen as an acceptable format to support active learning in a corresponding environment.
Sonography	Everad F, Seifert L, Mansour N, Hofauer B, Knopf A, Offergeld C; 2024	RCT; The study developed and tested a blended-learning approach for teaching paranasal sinus ultrasound to medical students. The intervention group studied theory with videos, practiced at home using a dummy probe, and trained via Zoom with the Peyton method, while the control group trained in person on ultrasound devices. Both groups attended the same introductory seminar and completed a practical exam on head and neck ultrasound with six standardized sweeps.	Outcomes were checklist-based practical exam scores.	No significant difference in total scores (online: 91%, in-person: 92%); minor differences in specific areas.
Sonography	Heiberg J, Hansen LS, Wemmelund K, Sorensen AH, Ilkjaer C, Cloete E, Nolte D, Roodt F, Dyer R, Swanevelder J, Sloth E; 2015	Quasi-experimental; This study evaluates a blended learning approach that combines e-learning and hands-on training to teach point-of-care ultrasound. Participants completed an e-learning course followed by four hours of practical training, covering ultrasound skills such as transthoracic echocardiography, sonography, lung ultrasound and dynamic needle tip positioning.	Knowledge of the procedures used and adequacy of ultrasound image interpretations.	Knowledge and practical ultrasound-related skills improved after e-learning as well as after hands-on training.
Sonography	Khoury M, Fotsing S, Jalali A, Chagnon N, Malherbe S, Youssef N; 2020	Quasi-experimental; Participants attended a hands-on workshop on echocardiography, where they practiced scanning techniques to generate four cardiac views after studying a peer-designed, expert-validated ultrasound manual. The program aimed to evaluate the impact of point-of-care ultrasound training on students’ anatomical sonographic knowledge and ability to generate ultrasound images.	Improvements in sonographic anatomical knowledge and skill retention.	A statistically significant improvement in sonographic anatomical knowledge was found at post-test assessment. Students were able to generate all four cardiac views im comparison to only one and three views at pre-test assessment.
Sonography	Weimer A, Recker F, Vieth T, Buggenhagen H, Schamberger C, Berthold R, Berthold S, Stein S, Schmidmaier G, Kloeckner R, Neubauer R, Müller L, Weinmann-Menke J, Weimer J; 2024	Quasi-experimental; The study evaluated whether a certified postgraduate MSUS course could be adapted for undergraduate medical students using a blended-learning format. The program included pre-course e-learning (lecture notes, Moodle videos), two days of face-to-face theoretical and practical training, and post-course resources, followed by a written exam and a DOPS assessment. Two control groups were included: medical students without training and certified doctors with MSUS training. Skills taught included ultrasound equipment setup, probe handling, functional joint exams, identification of normal anatomy (shoulder, elbow, hip, knee, ankle), documentation, and interpretation of results.	Significant subjective improvement in self-perceived MSUS competencies, high satisfaction with course design, teaching materials, and tutors; theoretical and practical test results of undergraduates comparable to trained doctors and superior to untrained students.	Study group scored ≥ 80% in theory and ≥ 86% in practical exams, comparable to control group 2 and significantly better than control group 1; performed better than doctors in “communication” and “overall impression” but worse in “pathology detection”.
Sonography	Weimer JM, Recker F, Horn L, Kuenzel J, Dirks K, Ille C, Buggenhagen H, Börner N, Weimer AM, Vieth T, Lorenz L, Rink M, Merkel D, Dionysopoulou A, Ludwig M, Kloeckner R, Weinmann-Menke J, Müller L; 2024	Quasi-experimental; The study compared digital versus analog preparation methods within a flipped classroom approach to undergraduate ultrasound education. The digital group used an e-learning platform with interactive modules, multimedia, quizzes, and self-assessment tools, while the control group studied printed lecture notes with the same content. Both groups then attended a three-day structured ultrasound course including demonstrations, small-group practice, peer-tutoring, and reviews. Skills taught included transducer handling, patient guidance, systematic abdominal scanning, image interpretation, pathology recognition, and anatomical knowledge.	Both groups improved significantly in theoretical and practical skills; the digital group scored significantly higher in intermediate and final theoretical and practical assessments and rated their learning materials and training concept more positively.	The study group outperformed the control group in Theoryinter, Practiceinter, and Practicepost scores, showed higher motivation, and evaluated their preparation phase more positively; both groups achieved similar final theoretical scores but practical skills remained higher in the digital group.

### Specialized tools

This category includes tools for specific procedures (e.g. otoscopy, bronchoscopy) and digital toolkits with QR-code-accessed instructional videos. Specialized digital tools relate to specific health care procedures and are aimed directly at the acquisition of practical skills related to those.

Researchers in the included studies have developed and evaluated specialized tools such as dermatology toolkits and ear simulators to enhance practical skills training. These tools were sometimes paired with instructional videos, online modules or supervised practice sessions.

Studies consistently showed improved diagnostic accuracy, confidence, skill acquisition and higher student satisfaction, especially when combining specialized tools with blended or hands-on training methods.

Fields of application included specific dermatological surgical skills, cardiopulmonary resuscitation, infusion pump operation and otoscopy. A summary of the information can be found in Table 12.

**Table 12:** Specialized Tools

Area of application	Author(s); year	Intervention	Outcome(s)	Results
Basic surgical skills	Wittbecker LM, Pham C, Wohlgemuth LK, Hoang MA, Bandholz TC, Schuh S, Gihl J, Erfurt-Berge C, Gläser R, Welzel J; 2022	Quantitative, descriptive; Students utilized a dermatology skills toolkit that provided materials for practicing minor surgical procedures at home, guided by instructional videos accessed via QR codes. They later had the opportunity to reinforce their skills in supervised, in-person practice sessions.	Students’ self-reported improvements in practical skills, their motivation for further study or clinical internships in dermatology and their evaluation of the toolkit’s utility and impact on learning.	The majority of students indicated that the toolkit helped them improve their practical skills and a significant percentage expressed increased interest in dermatology internships. The toolkit received positive feedback from 82.2% of the students.
Cardiopulmonary resuscitation	Jang HW, Kim KJ; 2014	Mixed-methods, descriptive; Students were given access to OSCE videos and completed an online survey, followed by a semi-structured interview to gain deeper insights into their perceptions.	Participants’ perceptions of and satisfaction with the effectiveness of the online clinical video learning format was assessed. Furthermore, barriers were explored.	Students found the video-based format effective for their learning of clinical skills and in preparing for an OSCE. The number of videos students watched was associated with their self-efficacy and perceived preparedness for an OSCE performance. Lack of interaction possibilities was reported as a barrier.
Infusion pump operation	Terry VR, Moloney C, Bowtell L, Terry PC; 2016	Quasi-experimental; Nursing students in the intervention group trained exclusively with an online infusion pump emulator, simulating infusion preparation and administration. The control group received face-to-face training with a physical infusion pump, while a third group combined both approaches.	Assessment scores regarding infusion pump competence, completion time for assessment tasks and participants feedback on their learning experiences using the online infusion pump emulator.	No significant differences in competency scores between the online-only and on-campus-only groups were found. However, the blended group (online plus on-campus) achieved higher competence scores and completed tasks faster than the other two groups. Overall, students rated the online infusion pump emulator positively, particularly for its accessibility and realistic features.
Otoscopy	Hakimi AA, Lalehzarian AS, Lalehzarian SP, Azhdam AM, Nedjat-Haiem S, Boodaie BD; 2019	RCT; This study evaluates the effectiveness of a smartphone-driven otoscope for teaching otoscopy skills compared to a conventional otoscope. All participants were trained on their assigned device and had time to practice on a colleague’s ear.	Students filled out a questionnaire regarding their ability to visualize anatomical landmarks of the middle ear. Also, participants had to indicate their confidence in performing a middle ear examination using their device.	Significantly more students in the intervention group identified anatomical landmarks of the middle ear correctly. Participants who used the smartphone-enabled otoscope reported significantly higher confidence compared to those who used a conventional otoscope.
Otoscopy	Lee DJ, Fu TS, Carrillo B, Campisi P, Forte V, Chiodo A; 2015	Quasi-experimental; Participants practiced otoscopy on a model ear, reviewing 30 high-resolution images of normal and abnormal tympanic membranes and ear pathologies. The focus was on teaching otoscopy and identifying normal and abnormal middle ear anatomy.	Students’ confidence in using an otoscope and their learning of middle ear anatomy.	93% of participants reported increased confidence in using an otoscope and all rated the event’s quality as very good or excellent.
Otoscopy	Morris E, Kesser BW, Peirce-Cottler S, Keeley M; 2012	Cohort study, single group; The study developed and validated a novel ear simulator for teaching pneumatic otoscopy to medical students. Participants practiced otoscopy with the simulator, received instructor feedback and were assessed on their ability to diagnose middle ear effusions and apply appropriate pneumatic pressure.	Diagnostic accuracy in detecting middle ear effusion and the pneumatic pressure applied during otoscopy.	The intervention group diagnosed effusion more accurately than the control group. They also applied pressures within the proper range more consistently compared to the control group. Experts achieved 100% diagnostic accuracy and applied pressure within the appropriate range across all trials.

## Discussion

### Principal findings

The main objective of this review was to provide a holistic perspective on the areas of application, the types of digital interventions and the outcomes and results associated with their use in teaching practical skills in the academic health professions. While a few included studies addressed other academic health care professions, the vast majority of included studies represented the field of medicine. When it comes to introducing a digital intervention into the teaching of practical skills, the modality of the digital technology used varied greatly depending on the type of field in which it is used. Digital interventions and tools comprised VR and AR tools, various kinds of simulators as well as mobile applications, video-based learning formats and different sorts of blended and hybrid learning setups. A common conclusion of the publications reviewed was that digital tools have considerable potential for teaching practical skills in the field. The interventions were largely judged to work as expected.

Overall, the included studies indicate that digital teaching methods can meaningfully support the acquisition of practical skills when they provide structured practice opportunities and some form of guidance or feedback. Interventions that allowed repeated, self-paced engagement with realistic scenarios tended to produce the most consistent improvements. Digital approaches also demonstrated added value in expanding access to training situations that are difficult to reproduce in conventional settings. At the same time, outcomes varied considerably across studies, reflecting differences in instructional design, the precision of the digital tools and the complexity of the targeted skills. Studies employing interactive or task-focused components, rather than passive formats, generally reported stronger effects. Remote and mobile solutions broadened learning opportunities but relied heavily on clear instructional structures to be effective. Taken together, the findings point to meaningful but context-dependent benefits of digital methods, highlighting the importance of aligning the choice of technology with the specific skill and its learning objectives.

### Digital versus traditional approaches

Based on the synthesized evidence, several explicit recommendations can be made regarding the integration of digital didactic instruments into the teaching of practical skills in health professions education. Virtual and augmented reality, simulators, mobile applications, e-learning platforms as well as blended approaches consistently demonstrate advantages in learner engagement, confidence and in many cases, procedural accuracy and procedural knowledge retention [7, 29–32]. Compared with traditional teaching methods, these tools offer unique benefits such as safe, repeatable practice environments, flexible access to content, and opportunities for independent learning, which are particularly valuable for high-stakes or resource-intensive skills [7, 29–32]. However, digital methods should not be regarded as outright replacements but rather as complementary to established instructional strategies [33, 34]. While many studies show superior or at least equivalent outcomes, limitations such as cybersickness, limited haptic realism, technical constraints and mixed findings on long-term transfer of skills highlight the importance of thoughtful integration [35–40]. Consequently, digital instruments are best implemented in a blended model, combining their strengths in accessibility, engagement and scalability with the tactile and interpersonal elements of traditional teaching [7, 30–32, 39]. This hybrid strategy appears most promising to optimize skill acquisition, learner satisfaction and preparedness for clinical practice [31, 32, 35–37].

In our review, studies reporting no significant benefits of mobile applications or e-learning platforms over conventional training shared some common characteristics. For mobile applications, interventions relying mainly on static or video-based content tended to show no measurable advantage [41–43]. For example, an Instagram-based mobile learning tool for nursing injections did not improve knowledge or skills compared to classroom teaching, as both groups received the same content and hands-on practice [41]. Similarly, a mobile-based video for urinary catheterization improved motivation but not objective skills [42]. Likewise, a cardiopulmonary resuscitation app performed no better than traditional training, while a blended approach yielded superior results [43]. In contrast, studies using interactive apps with structured feedback loops, such as modules for surgical simulation or ultrasound imaging, demonstrated stronger gains in performance and satisfaction [44–47].

A similar pattern was observed for e-learning platforms. Studies where digital instruction merely replicated existing teaching formats often found no significant differences compared to lectures or group-based teaching. For instance, teaching students practical oral-surgery skills by means of web-based courses did not demonstrate clear effectiveness in procedural skill improvements [48] and e-learning for abdominal examination improved performance only when interactive elements were added [49]. E-learning without a practical component, such as syringe pump training, showed no additional benefit compared to hands-on practice [50]. Likewise, in midwifery training, digital instruction was found equivalent to small-group teaching, though the latter yielded slightly higher OSCE scores in some modules [51]. By contrast, blended and interactive platforms that integrated digital modules with supervised skills practice consistently demonstrated higher effectiveness [52–54].

In summary, these findings suggest that the effectiveness of digital tools depends less on the medium itself and more on the didactic design [55]. Interactivity, structured feedback and integration with supervised practice emerge as critical factors [42, 48–54]. Purely content-heavy or passive digital formats appear insufficient to enhance skill acquisition, whereas blended approaches and active digital designs show clear advantages [51–53, 55].

### How can digital interventions support teaching and learning?

Digital interventions can support the teaching and learning of practical skills through different approaches, all of which have different characteristics, strengths and weaknesses.

VR tools emerged as particularly effective for high-risk and complex procedures, such as surgical interventions [38, 56–60]. While these tools create fully computer-generated environments, our findings show that Augmented Reality (AR) systems are more frequently used for immediate, scenario-based support, offering step-by-step guidance for practical skills [35–37].

Unlike VR, AR enhances the existing real-world setting rather than replacing it, which, makes AR especially suitable for specific skill-based training contexts in real-life environments [29, 35, 59, 60].

Simulators are among the most established digital tools for practical skills training, likely because they offer controlled, repeatable and realistic environments for practice [7, 30–64]. The simulators in the included studies also varied in terms of realism, difficulty and fidelity, making them an excellent tool for practicing surgical procedures in a risk-free environment [65–68].

Mobile applications also stood out in our analysis for their ability to support location-independent learning, enabling users to acquire and refine skills without being tied to a specific training setting [].

E-learning platforms, as identified in our included literature, provide structured modules that often integrate videos, animations and quizzes to teach practical skills effectively [32]. These platforms frequently include Massive Open Online Courses (MOOCs), which extend access to educational resources to large audiences online and often combine multiple digital tools, such as video-based formats and telecommunication tools, to enrich the learning experience [53, 69].

Video-based learning formats support the acquisition of practical skills by providing visual demonstrations, process-oriented walkthroughs and recorded lectures, which learners can review at their own pace [70, 71]. These asynchronous teaching models, as identified in our analysis, offer a flexible alternative to traditional methods and promote self-directed learning [72, 73].

In the included studies, serious games and gamified learning formats utilize features such as challenges, rewards and quizzes to create interactive learning scenarios that boost learner engagement and motivation [74, 75]. These approaches contribute to the development of decision-making skills and procedural knowledge in a safe, virtual environment, with notable examples including games focused on blood transfusion protocols or emergency response scenarios designed to enhance critical thinking and practical competencies [76, 77].

Our data indicate that telecommunication tools—including videoconferencing platforms, telementoring and telesimulation systems—enable synchronous interaction between instructors and learners, supporting location-independent mentoring and training [78]. These tools are especially valuable for delivering expert instruction in geographically remote settings [79, 80]. Furthermore, such tools allow learners to practice a range of skills, such as suturing and neurodynamic techniques, while benefiting from real-time feedback, visual comparisons with expert demonstrations and interactive Q&A—making it possible to engage in skills training both at home and in remote areas [81].

Interactive learning systems employ multimedia elements, virtual patient scenarios and adaptive feedback mechanisms to create engaging and dynamic educational settings [34, 82]. These systems frequently feature case-based simulations that allow learners to interact with virtual patients and receive immediate feedback on their clinical decisions and actions [83]. Our findings also show that the ability to adjust difficulty levels and tailor content to individual learner needs supports the effective acquisition of practical skills [84].

Robotic simulators, including systems like the da Vinci Skills Simulator, offer trainees a highly realistic environment for practicing robotic-assisted surgical procedures [85, 86]. These simulations play a critical role in skill development prior to operating on live patients, enhancing patient safety and providing a practical, application-oriented training context [87, 88].

Blended learning formats—which integrate digital and in-person instructional methods—enhance flexibility and support better procedural knowledge retention by linking theoretical learning with hands-on application [55, 89]. Our findings also indicate that completing e-learning modules prior to attending face-to-face training sessions can significantly improve the retention of practical skills [90–93].

Specialized tools—such as otoscopy and bronchoscopy simulators, along with digital toolkits that include instructional videos accessible via QR codes, are frequently designed to support the development of specific clinical skills in a structured and interactive manner [94–96].

### COVID-19

Almost a third of all included articles were published within the last five years, covering the period of the COVID-19 pandemic. This is comprehensible, as the pandemic boosted the development and adoption of digital tools to maintain the delivery of practical skills when face-to-face meetings were not possible [97]. In line with this reasoning, the use of digital tools for teaching in the academic health professions has received broad political support [98]. This and the general advance of digitalization are mutually reinforcing in the ongoing process beyond the COVID-19 pandemic [99]. While digital teaching methods have proven valuable during the pandemic - especially for theoretical knowledge and self-directed learning - limitations have become apparent in the acquisition of practical and social skills [100]. As a result, many institutions have since returned to face-to-face formats for hands-on training while adopting a more deliberate, blended approach to digital teaching methods [101, 102]. In this context, this scoping review can be used to assist in the decision-making process about an appropriate digital teaching method, leading to a more conscious use of digital methodology.

### Comparison with prior work

This scoping review provides a holistic perspective on the use of digital tools for the acquisition of practical skills for the academic health professions. Previous studies have tended to focus on specific professions, such as physiotherapy, nursing or medicine [103–106]. Other works focused on a single aspect of improving teaching and learning, such as developing a curriculum to promote job-specific digital skills or establishing a digital competence scale for teachers [107, 108]. While it is important to implement various concepts to concretely improve digital skills instruction, previous research lacks a general overview of how digital interventions and tools can improve the application-oriented teaching of procedural skills. In this way, this review is intended to support lecturers and trainers in selecting a suitable digital tool for teaching practical skills. Findings from previous studies indicate that both students and teachers have largely positive views of digital tools [109]. These findings are supported by the studies included in our review. Benefits identified included increased motivation and engagement, higher flexibility as well as improved learning outcomes [109].

### Ethical considerations in the application of digital methods

While the integration of digital tools into the education of academic health professionals brings benefits, it also raises concerns about the ethical considerations that teachers and lecturers need to address to ensure responsible use [110]. These considerations apply to privacy and data security issues, such as the collection and storage of student data through educational technologies [111]. Another form of ethical consideration is imposed by equity and access. Not all students have equal access to digital tools and resources, which can lead to learners being excluded from education, for example due to financial constraints or geographical remoteness [112]. In addition, the use of AI and ML may introduce biases in the form of questionable comprehensibility and accountability of AI- or ML-based technology [113, 114]. A significant obstacle to the implementation of AI-based systems is the use of opaque algorithms—often referred to as black boxes—which are widely considered unacceptable [115]. These ethical concerns may be addressed by fostering critical thinking about AI transparency and accountability [116]. Students are to be encouraged to question how AI-systems make decisions and introduce concepts like explainable AI to promote informed and reflective use of such technologies [117, 118]. In the context of education, setting an example of the responsible use of AI as a teacher creates the basis for ethical digital competence [118, 119].

### Limitations

In the existing literature, other terms or synonyms may be used to describe digital tools and their application in healthcare education. Therefore, there may be relevant studies that could not be identified with our search strategy and were therefore not included in this review. Another constraint arises from the fact that this review specifically addresses undergraduate healthcare education. Consequently, any studies related to postgraduate healthcare education were not included. Nevertheless, the digital tools and interventions used in these included studies could potentially be adapted to the level of postgraduate education. In addition, the literature search was limited to works written in English and German, which means that regions where other languages are spoken may be underrepresented in this review.

Another limitation is the exclusion of additional databases, such as PsycINFO, EMBASE, or ERIC. Although substantial overlap exists with the databases included in our search strategy, the absence of these sources may have led to the omission of some relevant studies.

There also is the potential for publication bias, as studies with positive findings may be more likely to be published than those reporting neutral or negative results. This may lead to an overrepresentation of favorable outcomes in the available evidence.

Lastly, another limitation concerns the categorization of studies with multi-component digital interventions. Some studies combined several tools (e.g., simulators with video-based learning or blended digital elements), yet for reasons of clarity each study was assigned to a single primary intervention category based on its predominant focus. This approach inevitably simplifies the complexity of the intervention landscape and should be considered when interpreting the findings.

### Future works

While the included studies describe the benefits, outcomes and results of digital tools in teaching practical skills for health professions, current research tends to focus on the field of medicine. Future research efforts could address this and provide a more general view of the benefits and challenges of digital interventions and tools for student education that can be applied to other health professions. There is also a lack of longitudinal studies that examine the retention of procedural skills over a longer period of time. A detailed investigation of how well learners retain skills over time using different digital tools could be of great interest to support decision-making when choosing which digital tool to use in education. Lastly, research on the cost-effectiveness and scalability of digital tools remains limited [120]. When introducing new digital interventions and tools in the education sector, economic feasibility is an important factor for stakeholders [121]. Only one study in our review specifically addressed cost-benefit aspects: Haerling conducted a cost-utility analysis comparing virtual and mannequin-based simulation [122]. The study found that, while learning outcomes did not differ significantly between the two modalities, virtual simulation offered a more cost-effective alternative due to lower resource requirements. This highlights the important role economic considerations play in choosing between different simulation approaches [122]. The development and implementation of digital interventions can be highly resource-intensive, as illustrated by the example of a virtual operating room tour in the study by Dubinski et al. [123]. According to the researchers’ example, production costs for this single digital tool amounted to over $50,000, including personnel, equipment and software expenses. However, such cost estimates are rarely included in the academic literature. Future research should report detailed cost breakdowns to support practical planning and scalability. Additionally, the integration of these technologies into curricula requires careful instructional design, which also demands time and financial investment [123, 124]. Accordingly, future studies should include this in their investigations and present and evaluate factors such as resource allocation, costs and required technological infrastructure [124]. Future research may benefit from more multidimensional approaches that link digital methods to the skills they support. This could offer deeper insight into their role in practical skills training.

## Conclusions

Our scoping review analyzed 292 research articles and identified 12 major forms of digital interventions currently used in the education of practical skills in the academic health professions. These include various forms of digital reality such as VR and AR, simulation-based learning systems, mobile applications and different forms of blended learning approaches. Across intervention types, digital methods demonstrated added value primarily by enabling structured, repeatable practice opportunities and by providing immediate, individualized feedback—two elements consistently associated with improved procedural performance. These cross-cutting benefits illustrate why digital tools can meaningfully complement conventional instruction in practical skills training. The included studies highlight the benefits, distinct fields of application and results of implemented digital tools. The variety of digital interventions and their unique strengths highlight their potential to enhance practical skills training in academic education. It has become clear that the type of digital tool used is closely tied to the area of application. Teachers need to make an informed decision about which digital teaching method to use for their particular subject area. The scoping review presented here is intended to support this process and provide guidance for comprehensive decision-making.

## Supplementary Information

Supplementary Material 1. PRISMA-ScR (Preferred Reporting Items for Systematic Reviews and Meta-Analyses extension for Scoping Reviews) checklist

Supplementary Material 2. Complete search strategy

## Data Availability

The datasets used during the current study are available from the corresponding author on reasonable request.
