# Correction: The role of digital teaching methods in supporting practical skills training in the academic training of health professions –a scoping review

**DOI:** 10.1186/s12909-026-09310-8

**Published:** 2026-04-28

**Authors:** Tjorven Stamer, Nora Machinek, Jost Steinhäuser, Mathilde Goujard, Kristina Flägel

**Affiliations:** Institute of Family Medicine, University Hospital Schleswig-Holstein, Campus Luebeck, Ratzeburger Allee 160, 23562 Luebeck, Germany

**Correction: BMC Med Educ 26, 361 (2026)**

**https://doi.org/10.1186/s12909-026-08785-9**

Following publication of the original article [1], the authors would like to transpose given and family names of authors.

The author group has been updated above and the original article [1] has been corrected.
